# Enantioselective synthesis of 2-oxazolidinones by ruthenium(ii)–NHC-catalysed asymmetric hydrogenation of 2-oxazolones†
†Electronic supplementary information (ESI) available. CCDC 1584965. For ESI and crystallographic data in CIF or other electronic format see DOI: 10.1039/c8sc01869c


**DOI:** 10.1039/c8sc01869c

**Published:** 2018-06-28

**Authors:** Wei Li, Marco Wollenburg, Frank Glorius

**Affiliations:** a Organisch-Chemisches Institut , Westfälische Wilhelms-Universität Münster , Corrensstraße 40 , 48149 Münster , Germany . Email: glorius@uni-muenster.de

## Abstract

An efficient synthesis of optically active 4-substituted 2-oxazolidinones by the ruthenium(ii)–NHC-catalysed asymmetric hydrogenation of 2-oxazolones was developed. Excellent enantioselectivities (up to 96% *ee*) and yields (up to 99%) were obtained for a variety of substrates, bearing a range of functional groups and useful motifs. The hydrogenation reaction was successfully scaled up to gram scale using low catalyst loading. Moreover, the utility of this methodology was demonstrated by the transformation of the enantioenriched product into the corresponding chiral β-amino alcohol, a bisoxazoline ligand, and the formal synthesis of (–)-aurantioclavine.

## Abstract 2

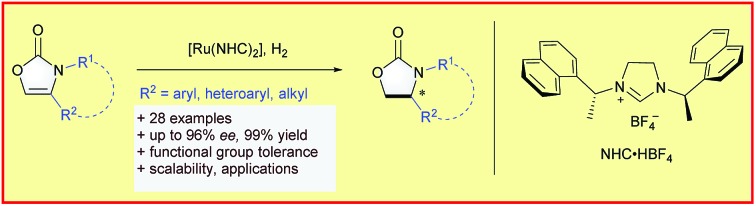
A highly enantioselective synthesis of optically active 4-substituted 2-oxazolidinones was described *via* ruthenium(ii)–NHC-catalysed asymmetric hydrogenation of 2-oxazolones.

## Introduction

**Scheme 1:**
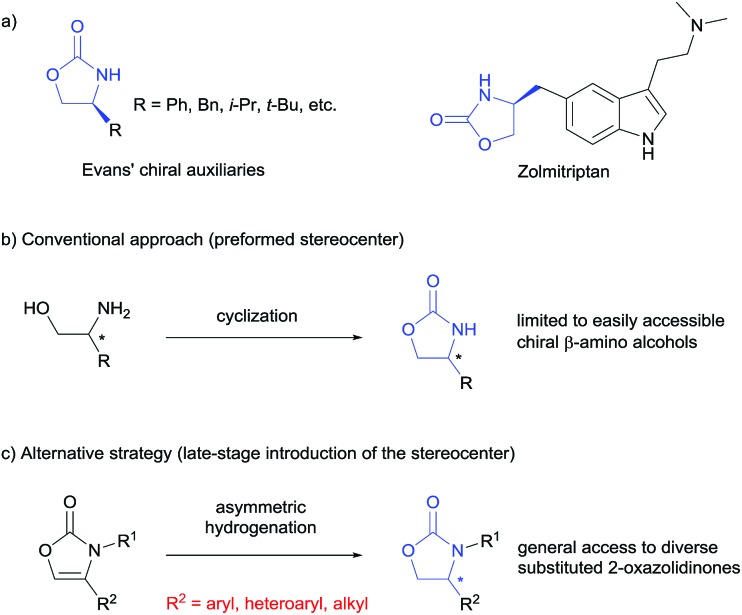
Applications and synthesis of chiral 2-oxazolidinones.

Chiral 2-oxazolidinones, widely used as Evans' chiral auxiliaries (Scheme 1a, left), play a prominent role in modern organic synthesis.^1^ Based on chiral 2-oxazolidinone auxiliaries, a wide range of asymmetric transformations has been developed to construct new chiral building blocks, which are frequently used in both natural product synthesis and drug discovery.^2^ Furthermore, the chiral 2-oxazolidinone motif itself is common in pharmaceutically relevant molecules (Scheme 1a, right).^3^ Thus, the synthesis of chiral 2-oxazolidinones has already attracted considerable attention. Conventionally, enantioenriched 2-oxazolidinones are synthesized by cyclization of the corresponding optically pure β-amino alcohols with C1-building blocks like phosgene and its derivatives (Scheme 1b). These methods often require toxic reagents and the pre-construction of the key stereocenter of the β-amino alcohols arises synthetic problems if they cannot be formed from natural enantiopure amino acids or related precursors.^2^ Considering this, the exploration of orthogonal, efficient and divergent catalytic strategies for the construction of diverse 2-oxazolidinone derivatives is highly important for organic synthesis and drug discovery.

In the last decade, the asymmetric hydrogenation of unsaturated heterocycles has emerged as a conceptually powerful method to produce optically active cyclic compounds and has received significant attention.^4^ In this regard, a synthetic method utilizing the enantioselective hydrogenation of 2-oxazolones for the late-stage construction of the key stereocenter can be envisioned as a powerful alternative to prepare diverse optically active 2-oxazolidinones (Scheme 1c). Moreover, this strategy would also provide a general way to produce optically active β-amino alcohols since the transformation from 2-oxazolidinones to free β-amino alcohols is very convenient.^2^ Recently, Zhang and co-workers reported the rhodium-catalysed asymmetric hydrogenation of 2-oxazolones, which afforded 4-aryl substituted 2-oxazolidinones with moderate enantioselectivities.^5^ To the best of our knowledge, this is the only precedent of an enantioselective synthesis of chiral 2-oxazolidinones by asymmetric hydrogenation of unsaturated heterocycles. As a continuous effort in the field of arene and heterocycle hydrogenation,^6^ we herein describe a highly enantioselective and practical hydrogenation of a broad scope of 2-oxazolones to access diverse enantioenriched 2-oxazolidinones catalysed by a ruthenium(ii)–N-heterocyclic carbene (NHC) complex.

## Results and discussion

Initially, the hydrogenation of 4-phenyloxazol-2(3*H*)-one (**1a**) was attempted under 50 bar H_2_ in *n*-hexane at room temperature in the presence of our previously-developed ruthenium(ii)–NHC catalyst, which is prepared *in situ* from [Ru(2-methylallyl)_2_(COD)], the NHC precursor (*R*,*R*)-SINpEt·HBF_4_, and NaO*t*-Bu.^7^,^8^ However, the desired product was not observed, presumably due to catalyst deactivation by coordination of the metal with free N–H (Table 1, entry 1). To circumvent catalyst deactivation, a variety of protecting groups were investigated, of which the 4-methoxybenzyl (PMB) protected carbamate **1d** was found to be suitable for hydrogenation, providing the desired 4-phenyloxazolidin-2-one with 85% *ee* and in 95% isolated yield (entry 2–4). To further improve the reaction conditions, a solvent screen was conducted (entries 4–8). No reaction occurred in dichloromethane presumably due to catalyst decomposition (entry 5), and cyclohexane (entry 8) was found to be slightly superior to other solvents (*n*-hexane, toluene and THF) for enantioselectivity. Decreasing the reaction temperature to 0 °C further improved enantiocontrol, providing the desired product with 95% *ee* and in 93% yield (entry 9). Finally, a solvent mixture of cyclohexane/THF = 20/1 was used to improve the solubility of the substrate, to afford the chiral 2-oxazolidinone **2d** in 99% yield and 95% *ee* (entry 10).

**Table 1:** Optimisation of the reaction conditions^*a*^

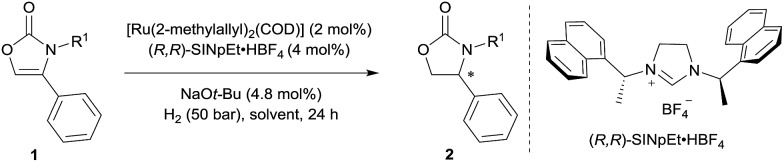					
Entry	R^1^	Solvent	*T* (°C)	Yield^*b*^ (%)	*ee* ^*c*^ (%)
1	H (**1a**)	*n*-Hexane	25	0	—
2	Boc (**1b**)	*n*-Hexane	25	Traces	N. D.
3	Ac (**1c**)	*n*-Hexane	25	0	—
4	PMB (**1d**)	*n*-Hexane	25	95	85
5	PMB (**1d**)	CH_2_Cl_2_	25	0	—
6	PMB (**1d**)	Toluene	25	99	85
7	PMB (**1d**)	THF	25	99	87
8	PMB (**1d**)	Cyclohexane	25	98	88
9	PMB (**1d**)	Cyclohexane	0	93	95
10^*d*^	PMB (**1d**)	Cyclohexane	0	99	95

^*a*^General conditions: [Ru(2-methylallyl)_2_(COD)] (0.10 mmol), (*R*,*R*)-SINpEt·HBF_4_ (0.20 mmol) and NaO*t*-Bu (0.24 mmol) were stirred at 70 °C in *n*-hexane (5.0 mL) for 16 h to perform the catalyst (0.02 M), after which 0.1 mL of the catalyst suspension were added to substrates **1a–d** (0.10 mmol) in the indicated solvent (1.0 mL), and the hydrogenation was performed at 50 bar H_2_ for 24 h.

^*b*^Yields of isolated product after column chromatography are reported.

^*c*^Determined by HPLC analysis using a chiral stationary phase.

^*d*^Using a solvent mixture of cyclohexane/THF = 20/1. Boc = *tert*-butyloxycarbonyl, Ac = acetyl, PMB = 4-methoxybenzyl. N. D. = not determined.

**Scheme 2:**
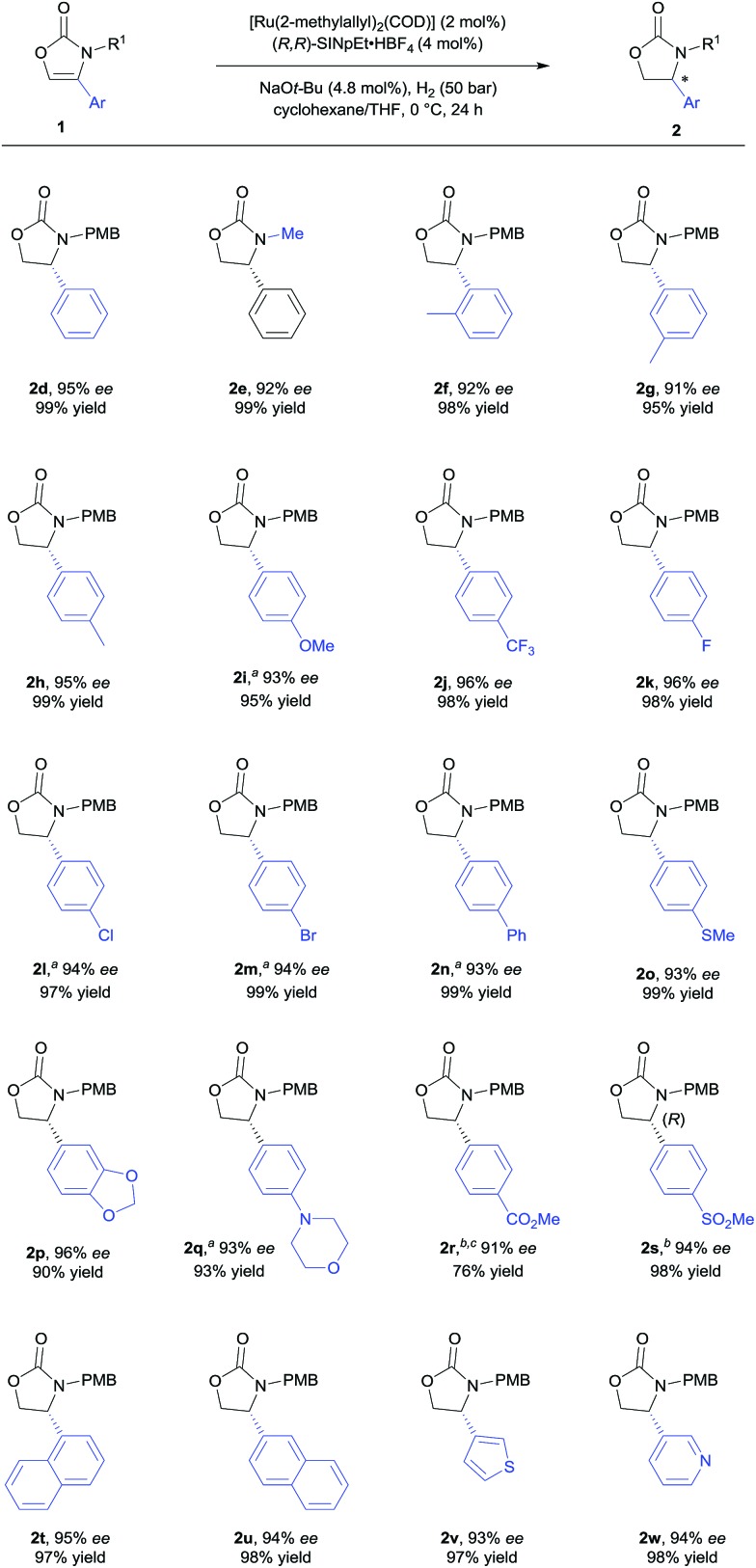
Substrate scope of 4-aryl or 4-heteroaryl substituted 2-oxazolones. General conditions: [Ru(2-methylallyl)_2_(COD)] (0.10 mmol), (*R*,*R*)-SINpEt·HBF_4_ (0.20 mmol) and NaO*t*-Bu (0.24 mmol) were stirred at 70 °C in *n*-hexane (5.0 mL) for 16 h to perform the catalyst (0.02 M), after which 0.2 mL of the catalyst suspension were added to substrates **1d–s** (0.20 mmol) in cyclohexane/THF (20/1), and the hydrogenation was performed under 50 bar H_2_ at 0 °C for 24 h. Yields of isolated products after column chromatography are reported. % *ee* values were determined by HPLC analysis using a chiral stationary phase. ^*a*^Using a solvent mixture of cyclohexane/THF (1/1). ^*b*^Using THF (2.0 mL). ^*c*^At –10 °C.

**Scheme 3:**
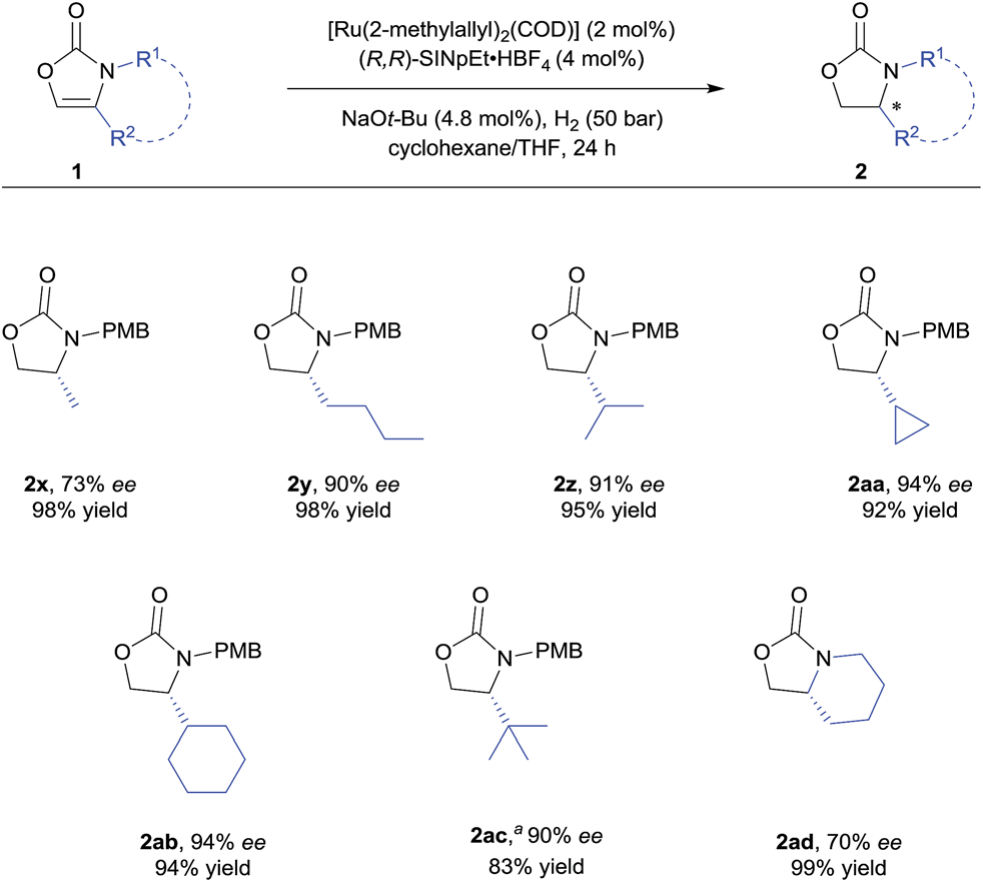
Substrate scope of 4-alkyl substituted 2-oxazolones. For detailed conditions, see ESI.† Yields of isolated products after column chromatography are reported. % *ee* values were determined by HPLC analysis using a chiral stationary phase. ^*a*^Using 5 mol% of the catalyst.

With the optimised reaction conditions in hand (Table 1, entry 10), the substrate scope of the reaction was explored (Schemes 2 and 3). First, the variation of the protecting group from *N*-PMB to *N*-methyl afforded the products with similar results (Scheme 2, **2d** and **2e**). Next, the positional influence of substituents on the phenyl ring was investigated. Methyl groups in the 2-, 3- and 4-positions were well-tolerated, providing the corresponding 2-oxazolidinones with excellent enantioselectivities and in high yields (**2f–h**). The electronic effect of the substituents was also examined. Both, electron-rich and electron-deficient substrates (**1i** and **1j** respectively) underwent hydrogenation to smoothly afford products **2i** and **2j**. Halogenated substrates **1k–m** were also used to provide the desired products **2k–m** with excellent enantioselectivities, in very high yields and without the formation of dehalogenated byproducts. Notably, the catalytic system showcased a robust reactivity, tolerating various functional groups and useful motifs (such as SMe, 1,3-benzodioxole, morpholine, CO_2_Me and SO_2_Me) to provide the corresponding products **2o–s** with 91–96% *ee* and in 76–99% yield. These functional groups and motifs (**2i–s**) provide an excellent opportunity for further applications of the 2-oxazolidinone products. In addition, the absolute configuration of **2s** was determined to be (*R*) by X-ray crystallographic analysis.^9^ The absolute configuration of all other products was assigned by analogy. Additionally, substrates with condensed-ring and heteroaromatic moieties were also tested. Both 1- and 2-naphthyl-substituted substrates were tolerated under the standard conditions (**2t** and **2u**). Remarkably, thiophene and pyridine containing substrates did not poison the Ru–NHC catalyst, producing the corresponding products (**2v** and **2w**) with 93% *ee* and 94% *ee* respectively.

We further explored the substrate scope with 4-alkyl substituents (Scheme 3). Alkyl substituted substrates with different steric demand were systematically tested. Simple 4-methyloxazolidin-2-one **2x** was obtained with moderate enantioselectivity. Better control of the stereoselectivity was observed when introducing *n*-butyl substituent (**2y**). Isopropyl, cyclopropyl, and cyclohexyl substituents were successfully employed, affording the corresponding products with 91–94% *ee* and in 92–95% yield (**2z–ab**). Finally, *tert*-butyl substituted 2-oxazolone **1ac** was tolerated to give the product **2ac** with 90% *ee* and in 83% yield. These results indicate that bulky alkyl groups are beneficial for the enantioinduction. In addition, bicyclic 2-oxazolidinone **2ad** was also obtained by the developed method, albeit with moderate *ee*.

**Scheme 4:**
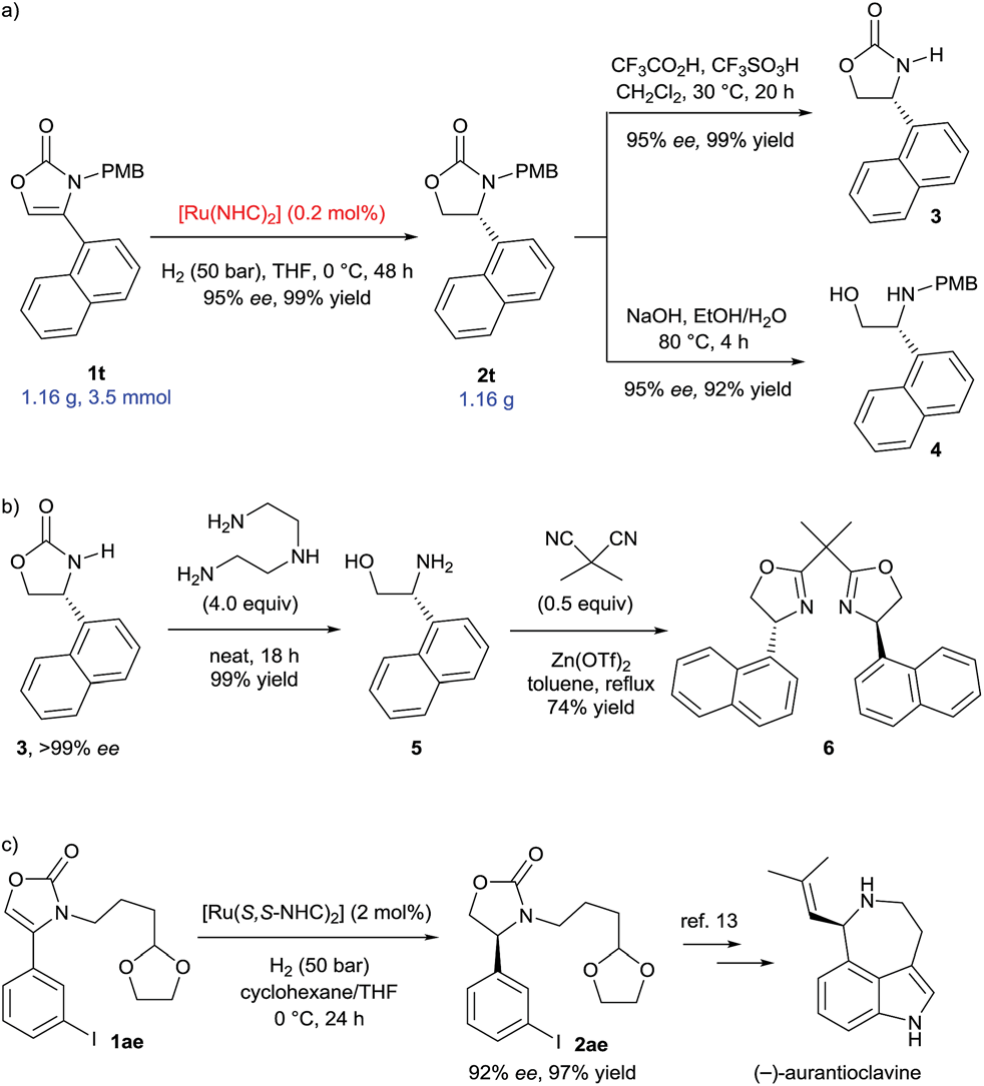
Scaled-up hydrogenation and transformations of the products.

We then proceeded to demonstrate applications of this methodology (Scheme 4). Scale-up of the hydrogenation of 4-(1-naphthyl) substituted substrate **1t** to gram-scale provided the corresponding 2-oxazolidinone **2t** with 95% *ee* and in 99% yield (Scheme 4a). Remarkably, the catalyst loading was successfully reduced to 0.2 mol%, which greatly increases the synthetic viability of this protocol. The deprotection of the PMB group was conveniently completed to afford **3** in 99% yield and without loss of enantiomeric excess.6*d* Hydrolysis of **2t** using NaOH liberated the *N*-PMB β-amino alcohol **4** in 92% yield. The enantiopurity of product **3** was readily increased to >99% *ee* after recrystallization from ethyl acetate. Optically pure β-amino alcohol **5** was then prepared in quantitative yield by cleavage of the 2-oxazolidinone **3** using diethylenetriamine (Scheme 4b).^10^ The absolute configuration of β-amino alcohol **5** was reaffirmed to be (*R*) by comparing the optical rotation to the literature data.^11^ The β-amino alcohol **5** was further transformed into 1-naphthyl-substituted bisoxazoline ligand **6** in 74% yield by reaction with dimethylmalononitrile and Zn(OTf)_2_.11a,^12^ Furthermore, aryl iodide and different *N*-alkyl substituted substrate **1ae** was well tolerated under the established reaction conditions (Scheme 4c). Hydrogenation of **1ae** using (*S*,*S*)-SINpEt·HBF_4_ as the carbene ligand precursor, furnished the oxazolidinone **2ae** (92% *ee* and 96% yield), a key synthetic intermediate employed in the synthesis of the alkaloid (–)-aurantioclavine.^13^

## Conclusions

In summary, we have developed a protocol for the catalytic enantioselective hydrogenation of 2-oxazolones to obtain optically active 2-oxazolidinone derivatives. The ruthenium(ii)–NHC catalyst system enabled a broad range of substrates to be successfully hydrogenated with excellent enantioselectivities (up to 96% *ee*) and in yields (up to 99%), thus to make the approach practical for the first time. Various functional groups and synthetically useful motifs were well-tolerated. The synthetic utility of this protocol was further demonstrated by performing a reaction on a gram-scale with a reduced catalyst loading; the obtained enantioenriched product was readily converted into an optically pure β-amino alcohol and subsequently a bisoxazoline ligand. The formal synthesis of (–)-aurantioclavine was enabled by the functional group tolerance towards an iodine substituent at the aryl ring and a varied *N*-alkyl chain.

## Conflicts of interest

There are no conflicts to declare.

## Supplementary Material

Supplementary information

Crystal structure data
