# Supplementary material for: Enantioselective synthesis of 2-oxazolidinones by ruthenium(ii)–NHC-catalysed asymmetric hydrogenation of 2-oxazolones
Source: Chem Sci. 2018 Jun 28;9(29):6260–3. doi: 10.1039/c8sc01869c (PMC6063072; doi:10.1039/c8sc01869c)

Electronic Supplementary Material (ESI) for Chemical Science.
This journal is © The Royal Society of Chemistry 2018

Enantioselective synthesis of 2-oxazolidinones by ruthenium(II)–NHC-catalysed asymmetric hydrogenation of 2-oxazolones

*Wei Li, Marco Wollenburg, and Frank Glorius**

Organisch-Chemisches Institut, Westfälische Wilhelms-Universität Münster,

Corrensstraße 40, 48149 Münster, Germany.

E-mail: glorius@uni-muenster.de

Supporting Information

CONTENTS:

(A) General 2

(B) Preparation of the substrates 3

(C) General procedure for the enantioselective hydrogenation 14

(D) Scaled-up reaction and transformations of the products 43

(E) References 50

(F) Copies of NMR spectra 50

(A) General

Unless otherwise noted, all reactions were carried out under an atmosphere of argon in flame-dried glassware. Reaction temperatures are reported as the temperature of the bath surrounding the vessel unless otherwise stated. The solvents used were purified by distillation over the drying agents indicated in parentheses and were transferred under argon: *n*-hexane (CaH_2), THF (Na-benzophenone), toluene (CaH_2).

All hydrogenation reactions were carried out in Berghof High Pressure Reactors using hydrogen gas. Commercially available chemicals were obtained from Acros Organics, Aldrich Chemical Co., Strem Chemicals, Alfa Aesar, ABCR, TCI Europe, Combi-Blocks and Chempur and used as received unless otherwise stated. Chiral amines for the preparation of NHC-ligands (*R,R*)-SINpEt•HBF_4, (*S,S*)-SINpEt•HBF_4 were received from BASF SE. NHC Ligands were synthesized following literature known procedures.[1] All analytical data was in agreement with the reported data.

Analytical thin layer chromatography was performed on Polygram SIL G/UV_{254} plates and alox B. Visualization was accomplished with short wave UV light, vanillin, ninhydrine and/or $KMnO_4$ staining solutions followed by heating. Flash chromatography was either performed on Merck silica gel (40–63 mesh) by standard technique eluting with solvents as indicated or alox B. GC-MS Spectra were recorded on an Agilent Technologies 7890A GC-system with an Agilent 5975C VL MSD or an Agilent 5975 inert Mass Selective Detector (EI) and a HP-5MS column (0.25 mm × 30 m, Film: 0.25 μm).

^{1}H, ^{13}C and ^{19}F NMR spectra were recorded on a Bruker AV 300 or AV 400, Varian 500 MHz INOVA or Varian Unity plus 600 in the indicated solvents. Chemical shifts (δ) are given in ppm relative to TMS. The residual solvent signals were used as references and the chemical shifts converted to the TMS scale ($CDCl_3$: δ_H = 7.26 ppm, δ_C = 77.16 ppm; CD_2Cl_2: δ_H = 5.32 ppm, δ_C = 53.8 ppm; CD_3OD: δ_H = 3.31 ppm, δ_C = 49.00 ppm). ^{19}F spectra were not calibrated by an internal

reference. The ^{1}H, ^{13}C and ^{19}F multiplicities of the signals are reported as s (singlet), bs (broad singlet), d (doublet), t (triplet), q (quartet), p (pentet) and m (multiplet). Coupling constants (*J*) are quoted in Hz. ESI mass spectra were recorded on a Bruker Daltonics MicroTof. Specific rotation was measured on a Perkin Elmer 341 polarimeter at 22°C using a quartz glass cell (100 mm path length). The enantiomeric excess (*ee*) of the products was determined by HPLC analysis using chiral column AS-H, AD-H and OD-H.

(B) Preparation of the substrates

Procedure A:

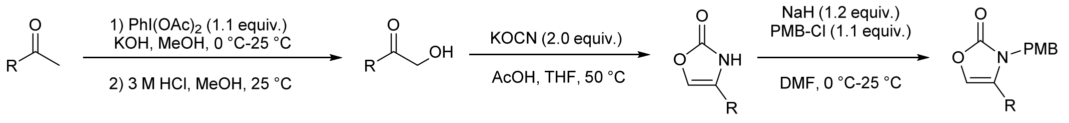

The substrates (**1d**–**x**, **1z**–**ac**) were synthesized according to a modified literature procedure.[2] The typical procedure is as follows: (Diacetoxyiodo)benzene (10.63 g, 33.0 mmol, 1.1 equiv.) was slowly added to a solution of the corresponding ketone derivative (30.0 mmol, 1.0 equiv.) in MeOH (60 mL) at 0 °C in an open flask. After stirring at 0 °C for 0.5 h, the reaction mixture was warmed to room temperature and was detected by TLC analysis until full consumption of starting material was observed. The reaction mixture was concentrated under reduced pressure, water (100 mL) was added and the mixture was extracted with EtOAc (3 x 100 mL). The volatiles were evaporated and the residue was dissolved in a mixture of MeOH (20 mL) and aqueous 3 M HCl (20 mL). After stirring overnight at room temperature, the crude mixture was concentrated under reduced pressure and purified by column chromatography on silica gel to provide pure α-hydroxy ketones (for **1d** and **1x**, α-hydroxy ketones were commercially available).

A solution of the corresponding α-hydroxy ketone derivative (1.0 equiv.), potassium cyanate (2.0 equiv.), acetic acid (2.4 equiv.) and THF (0.4 M) was stirred at 50 °C

until complete consumption of the starting material was indicated by TLC (generally overnight). The mixture was allowed to cool down to room temperature, quenched with water (30 mL), extracted with EtOAc (3 x 50 mL). The organic layers were combined, washed with saturated aqueous $NaHCO_3$ solution (50 mL), dried over $MgSO_4$, concentrated and purified by column chromatography on silica gel.

Sodium hydride (60% purity, 1.2 equiv.) was added portionwise to a solution of the corresponding oxazolone (1.0 equiv.) in DMF (0.5 M) at 0 °C. The mixture was stirred at 0 °C for 45 min, before *p*-methoxybenzyl chloride (1.1 equiv.) was added. The mixture was stirred at room temperature and after full consumption of the starting material, as indicated by TLC analysis (generally 5–6 h), the reaction was quenched with water. EtOAc was added and the organic layers were washed twice with 5wt% aqueous LiCl solution to remove DMF, followed by additional washing with brine. After drying over $MgSO_4$, the crude product was purified by column chromatography on silica gel. Solid substrates were further purified by recrystallization from EtOAc to give pure PMB-protected oxazol-2(3*H*)-ones.

3-(4-Methoxybenzyl)-4-phenyloxazol-2(3*H*)-one (1d)

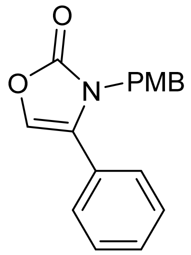

^{1}H NMR (400 MHz, $CDCl_3$) δ = 7.44 – 7.36 (m, 3H), 7.24 – 7.19 (m, 2H), 6.99 – 6.95 (m, 2H), 6.80 (s, 1H), 6.78 – 6.74 (m, 2H), 4.73 (s, 2H), 3.76 (s, 3H). ^{13}C NMR (101 MHz, $CDCl_3$): δ = 159.3, 156.6, 129.9, 129.7, 129.0, 129.0, 128.9, 128.4, 126.7, 124.2, 114.1, 55.4, 45.5. ESI-MS: calculated $[C_{17}H_{15}NO_3+Na]^+$: 304.0944, found: 304.0940.

3-Methyl-4-phenyloxazol-2(3*H*)-one (1e)

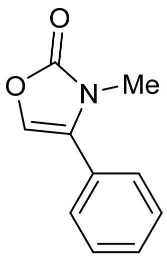

^{1}H NMR (300 MHz, $CDCl_3$) δ = 7.49 – 7.43 (m, 3H), 7.39 – 7.33 (m, 2H), 6.84 (s, 1H), 3.23 (s, 3H). ^{13}C NMR (75 MHz, $CDCl_3$): δ = 156.4,

130.1, 130.0, 129.2, 128.2, 126.6, 123.8, 29.5. ESI-MS: calculated $[C_{10}H_9NO_2+Na]^+$: 198.0525, found: 198.0531.

3-(4-Methoxybenzyl)-4-(*o*-tolyl)oxazol-2(3*H*)-one (1f)

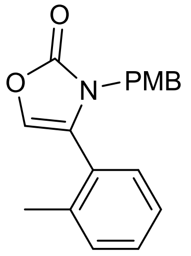

^{1}H NMR (400 MHz, $CDCl_3$) δ = 7.36 (td, J = 7.6, 1.3 Hz, 1H), 7.26 – 7.18 (m, 2H), 7.07 (dd, J = 7.6, 1.3 Hz, 1H), 6.84 – 6.78 (m, 2H), 6.72 – 6.65 (m, 3H), 4.49 (s, 2H), 3.75 (s, 3H), 2.02 (s, 3H). ^{13}C NMR (101 MHz, $CDCl_3$) δ = 159.3, 156.3, 138.7, 131.4, 130.5, 130.3, 129.6, 128.3, 127.9, 126.0, 125.8, 124.6, 113.9, 55.4, 45.3, 19.7. ESI-MS: calculated $[C_{18}H_{17}NO_3+Na]^+$: 318.1101, found: 318.1102.

3-(4-Methoxybenzyl)-4-(*m*-tolyl)oxazol-2(3*H*)-one (1g)

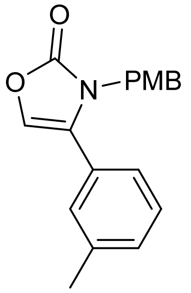

^{1}H NMR (400 MHz, $CDCl_3$) δ = 7.23 (d, J = 7.0 Hz, 1H), 7.21 – 7.17 (m, 1H), 7.01 – 6.93 (m, 4H), 6.76 – 6.72 (m, 3H), 4.68 (s, 2H), 3.73 (s, 3H), 2.29 (s, 3H). ^{13}C NMR (101 MHz, $CDCl_3$) δ = 159.3, 156.6, 138.8, 130.4, 130.0, 129.6, 129.0, 128.9, 128.5, 126.5, 125.9, 124.0, 114.1, 55.3, 45.5, 21.4. ESI-MS: calculated $[C_{18}H_{17}NO_3+Na]^+$: 318.1101, found: 318.1093.

3-(4-Methoxybenzyl)-4-(*p*-tolyl)oxazol-2(3*H*)-one (1h)

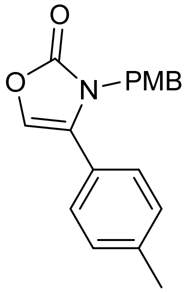

^{1}H NMR (400 MHz, $CDCl_3$) δ = 7.20 (d, J = 7.9 Hz, 2H), 7.13 – 7.08 (m, 2H), 7.02 – 6.97 (m, 2H), 6.80 – 6.77 (m, 1H), 6.77 – 6.75 (m, 2H), 4.71 (s, 2H), 3.77 (s, 3H), 2.39 (s, 3H). ^{13}C NMR (101 MHz, $CDCl_3$) δ = 159.3, 156.6, 139.9, 129.9, 129.7, 129.0, 128.9, 128.5, 123.9, 123.7, 114.1, 55.4, 45.4, 21.5. ESI-MS: calculated $[C_{18}H_{17}NO_3+Na]^+$: 318.1101, found: 318.1094.

3-(4-Methoxybenzyl)-4-(4-methoxyphenyl)oxazol-2(3*H*)-one (1i)

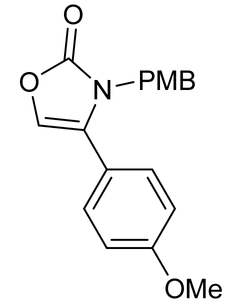

^{1}H NMR (400 MHz, $CDCl_3$) δ = 7.15 – 7.10 (m, 2H), 7.02 – 6.97 (m, 2H), 6.93 – 6.88 (m, 2H), 6.80 – 6.75 (m, 2H), 6.74 (s, 1H), 4.69 (s, 2H), 3.84 (s, 3H), 3.77 (s, 3H). ^{13}C NMR (101 MHz, $CDCl_3$) δ = 160.7, 159.3, 130.5, 129.6, 129.0, 128.6, 123.8, 118.8, 114.5, 114.1, 55.5, 55.4, 45.3. ESI-MS: calculated $[C_{18}H_{17}NO_4+Na]^+$: 334.1050, found: 334.1053.

3-(4-Methoxybenzyl)-4-(4-(trifluoromethyl)phenyl)oxazol-2(3*H*)-one (1j)

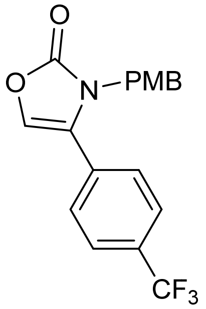

^{1}H NMR (300 MHz, $CDCl_3$) δ = 7.64 (d, J = 8.2 Hz, 2H), 7.34 (d, J = 8.2 Hz, 2H), 6.98 (d, J = 8.6 Hz, 2H), 6.88 (s, 1H), 6.78 (d, J = 8.6 Hz, 2H), 4.75 (s, 2H), 3.76 (s, 3H). ^{13}C NMR (75 MHz, $CDCl_3$) δ = 159.4, 156.4, 131.6 (q, J = 32.8 Hz), 130.3, 130.3, 129.0, 128.8, 128.0, 126.0 (q, J = 3.7 Hz), 125.0, 123.8 (q, J = 272.5 Hz), 114.3, 55.4, 45.8. ^{19}F NMR (282 MHz, $CDCl_3$): δ = −62.87 (s). ESI-MS: calculated $[C_{18}H_{14}NO_3F_3+Na]^+$: 372.0818, found: 372.0818.

4-(4-Fluorophenyl)-3-(4-methoxybenzyl)oxazol-2(3*H*)-one (1k)

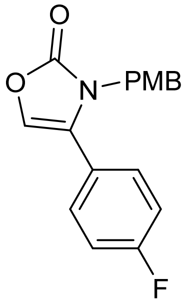

^{1}H NMR (300 MHz, $CDCl_3$) δ = 7.22 – 7.14 (m, 2H), 7.12 – 7.03 (m, 2H), 6.99 – 6.92 (m, 2H), 6.80 – 6.77 (m, 2H), 6.77 – 6.74 (m, 1H), 4.69 (s, 2H), 3.77 (s, 3H). ^{13}C NMR (75 MHz, $CDCl_3$) δ = 163.5 (d, J = 250.5 Hz), 159.3, 156.4, 131.0 (d, J = 8.4 Hz), 128.9, 128.8, 128.2, 124.3, 122.7 (d, J = 3.5 Hz), 116.2 (d, J = 21.9 Hz), 114.2, 55.4, 45.5. ^{19}F NMR (282 MHz, $CDCl_3$) δ = −110.51 (s). ESI-MS: calculated $[C_{17}H_{14}NO_3F+Na]^+$: 322.0850, found: 322.0845.

4-(4-Chlorophenyl)-3-(4-methoxybenzyl)oxazol-2(3*H*)-one (1l)

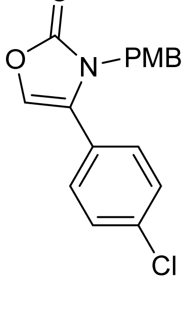

^{1}H NMR (400 MHz, $CDCl_3$) δ = 7.39 – 7.33 (m, 2H), 7.17 – 7.11 (m, 2H), 7.01 – 6.95 (m, 2H), 6.80 (s, 1H), 6.80 – 6.76 (m, 2H), 4.71 (s, 2H), 3.77 (s, 3H). ^{13}C NMR (101 MHz, $CDCl_3$) δ = 159.4, 156.5,

135.9, 130.2, 129.4, 128.9, 128.8, 128.2, 125.1, 124.4, 114.2, 55.4, 45.6. ESI-MS: calculated $[C_{17}H_{14}NO_3Cl+Na]^+$: 338.0554, found: 338.0555.

4-(4-Bromophenyl)-3-(4-methoxybenzyl)oxazol-2(3*H*)-one (1m)

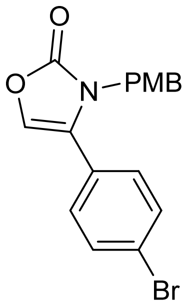

^{1}H NMR (400 MHz, $CDCl_3$) δ = 7.54 – 7.48 (m, 2H), 7.10 – 7.04 (m, 2H), 7.00 – 6.94 (m, 2H), 6.80 (s, 1H), 6.79 – 6.74 (m, 2H), 4.70 (s, 2H), 3.75 (s, 3H). ^{13}C NMR (101 MHz, $CDCl_3$) δ = 159.3, 156.4, 132.3, 130.3, 128.8, 128.8, 128.1, 125.5, 124.4, 124.0, 114.2, 55.3, 45.5. ESI-MS: calculated $[C_{17}H_{14}NO_3Br+Na]^+$: 382.0049, found: 382.0041.

4-([1,1'-Biphenyl]-4-yl)-3-(4-methoxybenzyl)oxazol-2(3*H*)-one (1n)

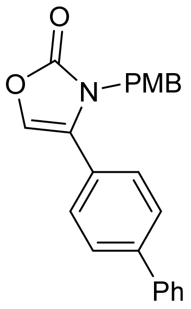

^{1}H NMR (400 MHz, $CDCl_3$) δ = 7.66 – 7.59 (m, 4H), 7.51 – 7.45 (m, 2H), 7.43 – 7.36 (m, 1H), 7.32 – 7.27 (m, 2H), 7.07 – 7.01 (m, 2H), 6.85 (s, 1H), 6.82 – 6.77 (m, 2H), 4.78 (s, 2H), 3.77 (s, 3H). ^{13}C NMR (101 MHz, $CDCl_3$) δ = 159.3, 156.6, 142.5, 140.0, 129.6, 129.2, 129.1, 128.9, 128.4, 128.1, 127.6, 127.2, 125.5, 124.2, 114.2, 55.4, 45.5. ESI-MS: calculated $[C_{23}H_{19}NO_3+Na]^+$: 380.1257, found: 380.1252.

3-(4-Methoxybenzyl)-4-(4-(methylthio)phenyl)oxazol-2(3*H*)-one (1o)

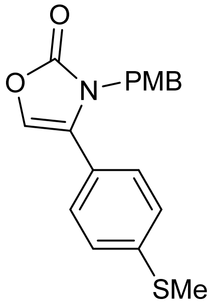

^{1}H NMR (300 MHz, $CDCl_3$) δ = 7.25 – 7.20 (m, 2H), 7.14 – 7.08 (m, 2H), 7.03 – 6.97 (m, 2H), 6.81 – 6.75 (m, 3H), 4.71 (s, 2H), 3.77 (s, 3H), 2.50 (s, 3H). ^{13}C NMR (75 MHz, $CDCl_3$) δ = 159.3, 156.6, 141.1, 129.5, 129.2, 128.9, 128.4, 126.2, 124.1, 122.8, 114.2, 55.4, 45.4, 15.4. ESI-MS: calculated $[C_{18}H_{17}NO_3S+Na]^+$: 350.0821, found: 350.0830.

4-(Benzo[*d*][1,3]dioxol-5-yl)-3-(4-methoxybenzyl)oxazol-2(3*H*)-one (1p)

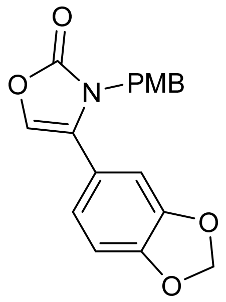

^{1}H NMR (400 MHz, $CDCl_3$) δ = 7.03 – 6.98 (m, 2H), 6.82 – 6.78 (m, 2H), 6.78 – 6.76 (m, 1H), 6.73 (s, 1H), 6.69 (dd, J = 8.0, 1.7 Hz, 1H), 6.64 (d, J = 1.7 Hz, 1H), 6.01 (s, 2H), 4.70 (s, 2H), 3.76 (s, 3H). ^{13}C NMR (101 MHz, $CDCl_3$): δ = 159.3, 156.4, 148.9, 148.1, 129.5, 128.9, 128.4, 124.0, 123.2, 120.0, 114.1, 109.2, 108.8, 101.7, 55.4, 45.3. ESI-MS: calculated $[C_{18}H_{15}NO_5+Na]^+$: 348.0842, found: 348.0835.

3-(4-Methoxybenzyl)-4-(4-morpholinophenyl)oxazol-2(3*H*)-one (1q)

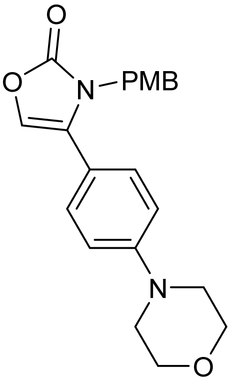

^{1}H NMR (300 MHz, $CDCl_3$) δ = 7.13 – 7.06 (m, 2H), 7.05 – 6.99 (m, 2H), 6.92 – 6.85 (m, 2H), 6.83 – 6.75 (m, 2H), 6.74 (s, 1H), 4.70 (s, 2H), 3.93 – 3.83 (m, 4H), 3.77 (s, 3H), 3.26 – 3.16 (m, 4H). ^{13}C NMR (75 MHz, $CDCl_3$) δ = 159.1, 156.5, 151.9, 131.1, 129.9, 128.8, 128.5, 123.5, 117.1, 115.1, 114.0, 66.7, 55.3, 48.5, 45.2. ESI-MS: calculated $[C_{21}H_{22}N_2O_4+Na]^+$: 389.1472, found: 389.1477.

Methyl 4-(3-(4-methoxybenzyl)-2-oxo-2,3-dihydrooxazol-4-yl)benzoate (1r)

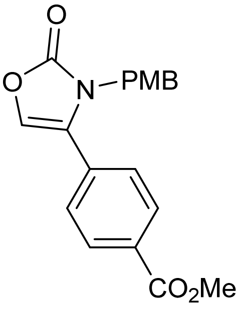

^{1}H NMR (300 MHz, $CDCl_3$) δ = 8.09 – 8.00 (m, 2H), 7.32 – 7.27 (m, 2H), 7.00 – 6.92 (m, 2H), 6.88 (s, 1H), 6.80 – 6.72 (m, 2H), 4.76 (s, 2H), 3.94 (s, 3H), 3.76 (s, 3H). ^{13}C NMR (75 MHz, $CDCl_3$) δ = 166.4, 159.4, 156.5, 131.1, 130.2, 129.1, 128.9, 128.4, 128.0, 125.0, 114.2, 55.4, 52.6, 45.8. ESI-MS: calculated $[C_{19}H_{17}NO_5+Na]^+$: 362.0999, found: 362.1003.

3-(4-Methoxybenzyl)-4-(4-(methylsulfonyl)phenyl)oxazol-2(3*H*)-one (1s)

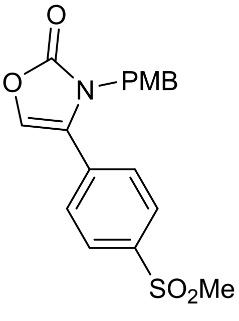

^{1}H NMR (400 MHz, $CDCl_3$) δ = 7.99 – 7.91 (m, 2H), 7.44 – 7.39 (m, 2H), 7.01 – 6.95 (m, 2H), 6.92 (s, 1H), 6.81 – 6.75 (m, 2H), 4.77 (s, 2H), 3.77 (s, 3H), 3.07 (s, 3H). ^{13}C NMR (101 MHz,

CDCl$_3$) δ = 159.5, 156.4, 141.4, 132.2, 129.2, 128.7, 128.4, 128.2, 127.8, 125.5, 114.4, 55.4, 46.0, 44.5. ESI-MS: calculated [$C_{18}H_{17}NO_5S$+Na]$^+$: 382.0720, found: 382.0718.

3-(4-Methoxybenzyl)-4-(naphthalen-1-yl)oxazol-2(3*H*)-one (1t)

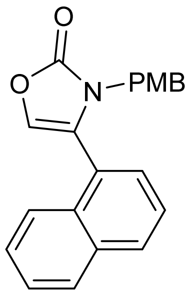

^{1}H NMR (400 MHz, CDCl$_3$) δ = 7.96 (d, J = 8.3 Hz, 1H), 7.91 (d, J = 8.2 Hz, 1H), 7.69 (d, J = 8.4 Hz, 1H), 7.59 – 7.40 (m, 3H), 7.26 – 7.23 (m, 1H), 6.87 (s, 1H), 6.67 – 6.60 (m, 2H), 6.60 – 6.53 (m, 2H), 4.48 (s, 2H), 3.70 (s, 3H). ^{13}C NMR (101 MHz, CDCl$_3$) δ = 159.2, 156.3, 133.5, 132.4, 130.7, 129.8, 129.5, 128.6, 128.2, 127.4, 126.7, 125.5, 125.2, 124.8, 123.6, 113.8, 55.3, 45.8. ESI-MS: calculated [$C_{21}H_{17}NO_3$+Na]$^+$: 354.1101, found: 354.1100.

3-(4-Methoxybenzyl)-4-(naphthalen-2-yl)oxazol-2(3*H*)-one (1u)

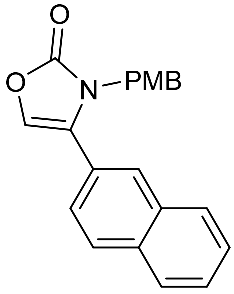

^{1}H NMR (400 MHz, CDCl$_3$) δ = 7.90 – 7.84 (m, 2H), 7.79 – 7.75 (m, 1H), 7.70 (s, 1H), 7.59 – 7.51 (m, 2H), 7.29 (dd, J = 8.5, 1.7 Hz, 1H), 7.05 – 6.99 (m, 2H), 6.90 (s, 1H), 6.79 – 6.74 (m, 2H), 4.79 (s, 2H), 3.76 (s, 3H). ^{13}C NMR (101 MHz, CDCl$_3$): δ = 159.3, 156.7, 133.5, 133.1, 130.1, 129.1, 128.9, 128.5, 128.5, 128.3, 128.0, 127.3, 127.1, 125.8, 124.5, 123.9, 114.2, 55.4, 45.8. ESI-MS: calculated [$C_{21}H_{17}NO_3$+Na]$^+$: 354.1101, found: 354.1099.

3-(4-Methoxybenzyl)-4-(thiophen-3-yl)oxazol-2(3*H*)-one (1v)

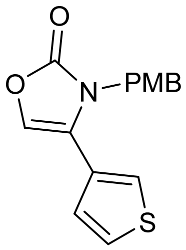

^{1}H NMR (400 MHz, CDCl$_3$) δ = 7.38 (dd, J = 5.0, 3.0 Hz, 1H), 7.20 (dd, J = 3.0, 1.3 Hz, 1H), 7.06 – 7.00 (m, 2H), 6.95 (dd, J = 5.0, 1.3 Hz, 1H), 6.85 (s, 1H), 6.84 – 6.78 (m, 2H), 4.76 (s, 2H), 3.77 (s, 3H). ^{13}C NMR (101 MHz, CDCl$_3$) δ = 159.3, 156.4, 128.8, 128.6, 128.4, 127.3, 127.1, 126.5, 125.4, 125.2, 124.4, 114.3, 114.1, 55.4, 45.5. ESI-MS: calculated [$C_{15}H_{13}NO_3S$+Na]$^+$: 310.0508, found: 310.0508.

3-(4-Methoxybenzyl)-4-methyloxazol-2(3*H*)-one (1x)

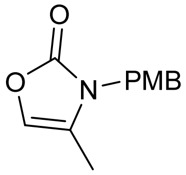

^{1}H NMR (400 MHz, $CDCl_3$) δ = 7.23 – 7.16 (m, 2H), 6.89 – 6.82 (m, 2H), 6.55 (q, J = 1.5 Hz, 1H), 4.70 (s, 2H), 3.78 (s, 3H), 1.90 (d, J = 1.6 Hz, 3H). ^{13}C NMR (101 MHz, $CDCl_3$) δ = 159.4, 156.6, 128.7, 128.3, 124.2, 123.4, 114.4, 55.4, 44.9, 9.2. ESI-MS: calculated $[C_{12}H_{13}NO_3+Na]^+$: 242.0788, found: 242.0802.

4-Isopropyl-3-(4-methoxybenzyl)oxazol-2(3*H*)-one (1z)

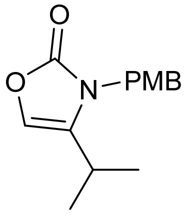

^{1}H NMR (400 MHz, $CDCl_3$) δ = 7.22 – 7.09 (m, 2H), 6.93 – 6.75 (m, 2H), 6.50 (dd, J = 3.1, 1.7, 1H), 4.78 – 4.67 (m, 2H), 3.78 (t, J = 2.4, 3H), 2.57 – 2.39 (m, 1H), 1.16 – 1.03 (m, 6H). ^{13}C NMR (101 MHz, $CDCl_3$) δ = 159.3, 156.9, 134.8, 128.3, 122.1, 114.2, 107.0, 55.3, 45.0, 23.7, 21.1. ESI-MS: calculated $[C_{14}H_{17}NO_3+Na]^+$: 270.1101, found: 270.1102.

4-Cyclopropyl-3-(4-methoxybenzyl)oxazol-2(3*H*)-one (1aa)

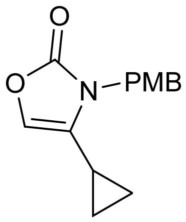

^{1}H NMR (300 MHz, $CDCl_3$) δ = 7.31 – 7.24 (m, 2H), 6.92 – 6.86 (m, 2H), 6.47 (d, J = 1.6 Hz, 1H), 4.83 (s, 2H), 3.82 (s, 3H), 1.39 – 1.24 (m, 1H), 0.81 (d, J = 6.4 Hz, 2H), 0.55 – 0.45 (m, 2H). ^{13}C NMR (75 MHz, $CDCl_3$) δ = 159.4, 156.5, 131.2, 129.0, 128.7, 123.3, 114.2, 65.2, 55.4, 45.2, 5.2, 4.1. ESI-MS: calculated $[C_{14}H_{15}NO_3+Na]^+$: 268.0944, found: 268.0942.

4-Cyclohexyl-3-(4-methoxybenzyl)oxazol-2(3*H*)-one (1ab)

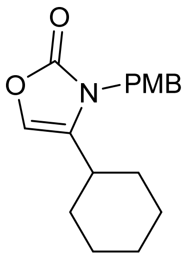

^{1}H NMR (400 MHz, $CDCl_3$) δ = 7.19 – 7.13 (m, 2H), 6.89 – 6.83 (m, 2H), 6.48 (d, J = 1.1 Hz, 1H), 4.72 (s, 2H), 3.79 (s, 3H), 2.17 – 2.04 (m, 1H), 1.83 – 1.70 (m, 4H), 1.30 – 1.06 (m, 6H). ^{13}C NMR (101 MHz, $CDCl_3$) δ = 159.2, 156.8, 134.0, 128.4, 128.4, 122.3, 114.2, 55.3, 45.1, 33.3, 31.8, 26.0, 25.7. ESI-MS: calculated

$[C_{17}H_{21}NO_3+Na]^+$: 310.1414, found: 310.1407.

4-(*tert*-Butyl)-3-(4-methoxybenzyl)oxazol-2(3*H*)-one (1ac)

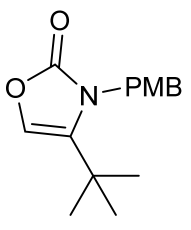

^{1}H NMR (300 MHz, $CDCl_3$) δ = 7.10 – 7.02 (m, 2H), 6.89 – 6.81 (m, 2H), 6.54 (s, 1H), 4.93 (s, 2H), 3.78 (s, 3H), 1.16 (s, 9H). ^{13}C NMR (75 MHz, $CDCl_3$): δ = 159.0, 157.7, 136.9, 128.5, 127.3, 122.3, 114.3, 55.4, 46.4, 30.7, 29.0. ESI-MS: calculated $[C_{15}H_{19}NO_3+Na]^+$: 284.1257, found: 284.1264.

Procedure B:

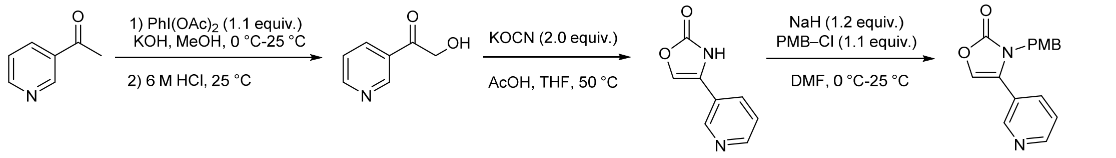

Method for substrate **1w**: (Diacetoxyiodo)benzene (10.63 g, 33.0 mmol, 1.1 equiv.) was slowly added to a solution of 1-(pyridin-3-yl)ethan-1-one (30.0 mmol, 1.0 equiv.) in MeOH (60 mL) at 0 °C in an open flask. After stirring at 0 °C for 1.5 h, the reaction mixture was concentrated under reduced pressure, water (100 mL) was added and the mixture was extracted with EtOAc (6 x 100 mL). The volatiles were evaporated and the residue was dissolved in aqueous 6 M HCl (20 mL). After stirring at room temperature for 24 h, the crude mixture was basified to pH > 10, extracted with CH_2Cl_2 (10 x 100 mL), dried over $MgSO_4$, concentrated under reduced pressure and purified by column chromatography on silica gel to provide 2-hydroxy-1-(pyridin-3-yl)ethan-1-one. After two more steps described in procedure A, 3-(4-methoxybenzyl)-4-(pyridin-3-yl)oxazol-2(3*H*)-one **1w** was obtained.

3-(4-Methoxybenzyl)-4-(pyridin-3-yl)oxazol-2(3*H*)-one (1w)

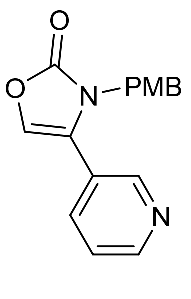

^{1}H NMR (400 MHz, $CDCl_3$) δ = 8.65 (dd, J = 4.9, 1.7 Hz, 1H), 8.49 (dd, J = 2.2, 0.8 Hz, 1H), 7.47 (dt, J = 7.9, 2.0 Hz, 1H), 7.31 (ddd, J = 7.9, 4.9, 0.8 Hz, 1H), 6.99 – 6.92 (m, 2H), 6.88 (s, 1H), 6.80 – 6.73

(m, 2H), 4.73 (s, 2H), 3.76 (s, 3H). ^{13}C NMR (101 MHz, $CDCl_3$) δ = 159.4, 156.3, 150.8, 149.4, 136.2, 128.8, 127.8, 126.7, 125.2, 123.6, 123.0, 114.3, 55.4, 45.7. ESI-MS: calculated $[C_{16}H_{14}N_2O_3+Na]^+$: 305.0897, found: 305.0924.

Procedure C:

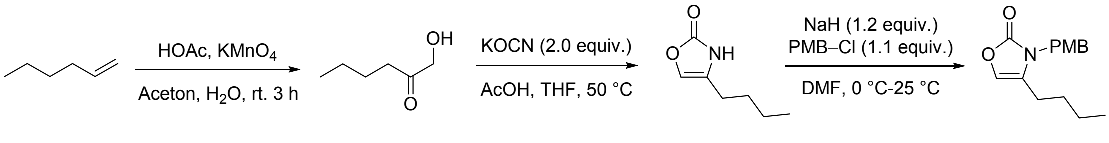

Method for substrate **1y**: A solution of $KMnO_4$ (5.31 g, 33.6 mmol, 1.6 equiv.) in acetone (66 mL) and deionized water (21 mL) was added to the mixture of hex-1-ene (21.0 mmol), acetone (168 mL), deionized water (39 mL) and glacial acetic acid (8.1 mL). The reaction mixture was stirred at 25 °C for 3 h. Saturated aqueous $NaHCO_3$ (200 mL) was poured into the reaction mixture and extracted with CH_2Cl_2 (3 x 200 mL). The combined organic layer was washed with brine (2 x 100 mL), dried (Na_2SO_4), and concentrated *in vacuo*. The resulting residue was purified by column chromatography on silica gel (5% EtOAc/hexane → 30% EtOAc/hexane) to give the 1-hydroxyhexan-2-one.[3] After two more steps described in Procedure A, 4-butyl-3-(4-methoxybenzyl)oxazol-2(3*H*)-one **1y** was obtained.

4-Butyl-3-(4-methoxybenzyl)oxazol-2(3*H*)-one (1y)

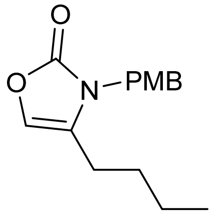

^1H NMR (400 MHz, $CDCl_3$) δ = 7.21 – 7.14 (m, 2H), 6.89 – 6.82 (m, 2H), 6.51 (t, J = 1.6 Hz, 1H), 4.70 (s, 2H), 3.79 (s, 3H), 2.24 – 2.14 (m, 2H), 1.48 – 1.27 (m, 4H), 0.87 (t, J = 7.2 Hz, 3H). ^{13}C NMR (101 MHz, $CDCl_3$) δ = 159.4, 156.8, 128.7, 128.6, 128.4, 123.1, 114.3, 55.4, 45.0, 28.3, 23.4, 22.3, 13.8. ESI-MS: calculated $[C_{15}H_{19}NO_3+Na]^+$: 284.1257, found: 284.1275.

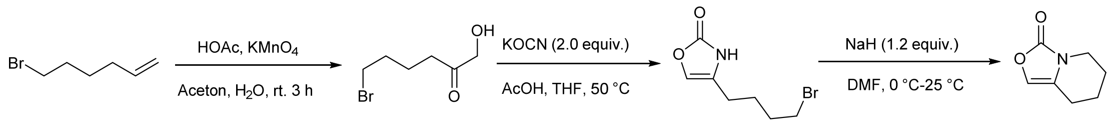

According to procedure C, 4-(4-bromobutyl)oxazol-2(3*H*)-one was obtained. Sodium hydride (60% purity, 1.2 equiv.) was added portionwise to a solution of the corresponding oxazolone (1.0 equiv.) in DMF (0.5 M) at 0 °C. After stirring at 0 °C for 30 min, the mixture was warmed to 30 °C. After full consumption of the starting material, as indicated by TLC analysis (24 h), the reaction was quenched with water. EtOAc was added and the organic layers were washed twice with 5wt% aqueous LiCl solution to remove DMF, followed by additional washing with brine. After drying over $MgSO_4$, the crude product was purified by column chromatography on silica gel. Final purification by recrystallization from EtOAc afforded 5,6,7,8-tetrahydro-3*H*-oxazolo[3,4-*a*]pyridin-3-one **1ad**.

5,6,7,8-Tetrahydro-3*H*-oxazolo[3,4-*a*]pyridin-3-one (1ad)

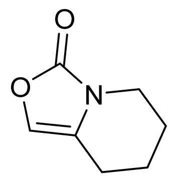

^{1}H NMR (400 MHz, $CDCl_3$) δ = 6.55 (t, J = 1.8 Hz, 1H), 3.55 (t, J = 6.2 Hz, 2H), 2.54 (td, J = 6.5, 1.7 Hz, 2H), 1.92 – 1.82 (m, 2H), 1.77 – 1.66 (m, 2H). ^{13}C NMR (101 MHz, $CDCl_3$) δ = 156.0, 124.2, 122.5, 40.8, 22.5, 20.0, 19.8. ESI-MS: calculated $[C_7H_9NO_3+Na]^+$: 162.0525, found: 162.0512.

(C) General procedure for the enantioselective hydrogenation

In a glove box, to a flame-dried screw-capped tube equipped with a magnetic stir bar was added [Ru(2-methylallyl)$_2$(COD)] (0.10 mmol; COD = cyclooctadiene), (*R,R*)-SINpEt·HBF$_4$ (0.20 mmol), and dry NaO*t*-Bu (0.24 mmol). The mixture was suspended in *n*-hexane (5.0 mL) and stirred at 70 °C for 16 h to form the catalyst mixture (0.02 M). To a glass vial, substrates (0.20 mmol), the indicated solvent, and 0.2 mL of the catalyst mixture (0.5 mL of the catalyst mixture was used for substrate **1ac**) was added under argon. The glass vial was placed in a 150 mL stainless steel autoclave under an argon atmosphere. The autoclave was pressurized and depressurized with hydrogen gas five times before 50 bar was set. The hydrogenation was performed at 50 bar H_2 for 24 h at indicated reaction temperature. After the autoclave was carefully depressurized, the mixture was directly purified by flash column chromatography on silica gel (*n*-pentane/EtOAc = 10/1, later 4/1, 2.5/1) to afford the desired product **2**. The yield was calculated based on the employed amount of the corresponding starting material. The enantiomeric excess (*ee*) of the product was determined by HPLC analysis using chiral columns AS-H, AD-H or OD-H.

3-(4-Methoxybenzyl)-4-phenyloxazolidin-2-one (2d)

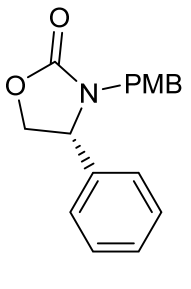

Colorless solid; cyclohexane/THF = 2 mL/0.1 mL, 0 °C, 99% yield, 95% *ee*. $[\alpha]_D^{22}$ = –86.7 (c = 1.00 in $CHCl_3$). HPLC DAICEL CHIRALCEL AS-H, *n*-hexane/2-propanol = 70/30, flow rate = 1 mL/min, λ = 254 nm, retention time: 17.4 min (major), 21.6 min (minor). ^{1}H NMR (400 MHz, $CDCl_3$) δ = 7.45 – 7.38 (m, 3H), 7.26 – 7.20 (m, 2H), 7.09 – 7.00 (m, 2H), 6.85 – 6.79 (m, 2H), 4.80 (d, *J* = 14.7, 1H), 4.55 – 4.45 (m, 2H), 4.15 – 4.04 (m, 1H), 3.79 (s, 3H), 3.58 (d, *J* = 14.7, 1H). ^{13}C NMR (101 MHz, $CDCl_3$) δ = 159.4, 158.4, 137.5, 130.1, 129.4, 129.2, 127.5, 127.3, 114.1, 70.0, 58.7, 55.4, 45.3. ESI-MS: calculated $[C_{17}H_{17}O_3+Na]^+$: 306.1101, found: 306.1114.

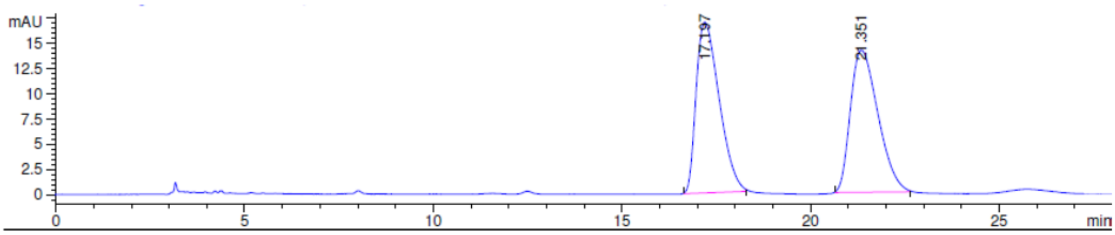

Peak #	RetTime [min]	Type	Width [min]	Area [mAU*s]	Height [mAU]	Area %
1	17.197	BB	0.5997	719.22839	16.92740	50.0845
2	21.351	BB	0.6958	716.80011	14.10785	49.9155

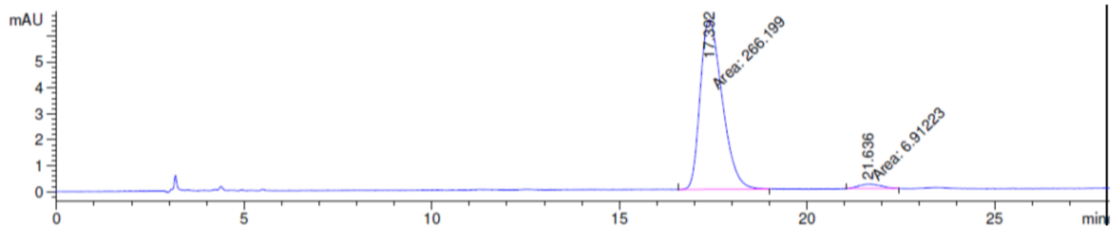

Peak #	RetTime [min]	Type	Width [min]	Area [mAU*s]	Height [mAU]	Area %
1	17.392	MM	0.6789	266.19861	6.53479	97.4691
2	21.636	MM	0.6847	6.91223	1.68266e-1	2.5309

3-Methyl-4-phenyloxazolidin-2-one (2e)

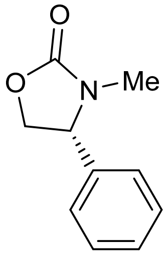

Colorless solid; cyclohexane/THF = 2.0 mL/0.1 mL, 0 °C, 99% yield, 92% *ee*. $[\alpha]_D^{22}$ = –61.2 (c = 1.02 in $CHCl_3$). HPLC DAICEL CHIRALCEL AS-H, *n*-hexane/2-propanol = 80/20, flow rate = 0.8 mL/min, λ = 210 nm, retention time: 25.7 min (major), 29.4 min (minor). ^{1}H NMR (300 MHz, $CDCl_3$) δ = 7.48 – 7.36 (m, 3H), 7.33 – 7.26 (m, 2H), 4.70 – 4.57 (m, 2H), 4.14 – 4.02 (m, 1H), 2.71 (s, 3H). ^{13}C NMR (75 MHz, $CDCl_3$) δ = 158.7, 137.6, 129.4, 129.1, 126.9, 69.8, 62.2, 29.3. ESI-MS: calculated $[C_{10}H_{11}NO_2+Na]^+$: 200.0682, found: 200.0685.

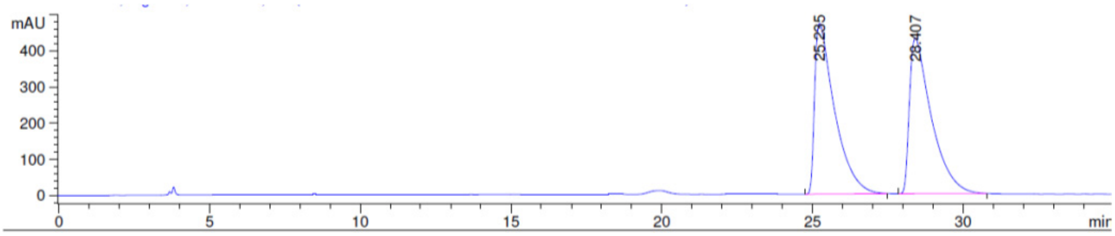

Peak #	RetTime [min]	Type	Width [min]	Area [mAU*s]	Height [mAU]	Area %
1	25.235	BB	0.6606	2.17791e4	472.60715	50.0207
2	28.407	BB	0.7310	2.17611e4	431.01498	49.9793

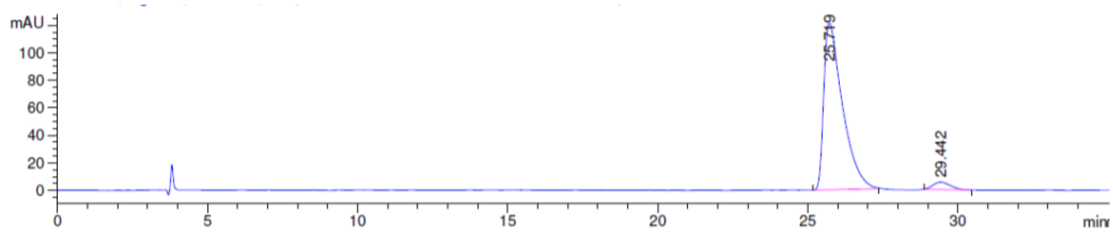

Peak #	RetTime [min]	Type	Width [min]	Area [mAU*s]	Height [mAU]	Area %
1	25.719	BB	0.6250	5256.51123	121.18918	95.9881
2	29.442	BB	0.5022	219.70189	5.40511	4.0119

3-(4-Methoxybenzyl)-4-(*o*-tolyl)oxazolidin-2-one (2f)

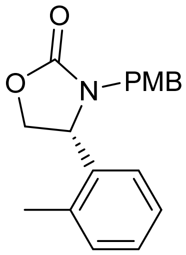

Colorless oil; cyclohexane/THF = 2.0 mL/0.1 mL, 0 °C, 98% yield, 92% *ee*. $[\alpha]_D^{22}$ = –88.3 (c = 2.15 in $CHCl_3$). HPLC DAICEL CHIRALCEL AD-H, *n*-hexane/2-propanol = 90/10, flow rate = 0.8 mL/min, λ = 230 nm, retention time: 17.4 min (minor), 18.9 min (major). ^{1}H NMR (300 MHz, $CDCl_3$) δ = 7.34 – 7.22 (m, 3H), 7.17 (d, *J* = 7.1, 1H), 7.07 – 6.93 (m, 2H), 6.88 – 6.75 (m, 2H), 4.87 (d, *J* = 14.6, 1H), 4.76 (dd, *J* = 8.8, 7.8, 1H), 4.53 (t, *J* = 8.7, 1H), 4.06 – 3.89 (m, 1H), 3.79 (s, 3H), 3.61 (d, *J* = 14.6, 1H), 2.07 (s, 3H). ^{13}C NMR (75 MHz, $CDCl_3$) δ = 159.4, 158.6, 135.9, 135.2, 131.1, 130.1, 128.5, 127.3, 127.2, 114.1, 69.3, 55.3, 45.3, 18.9. ESI-MS: calculated $[C_{18}H_{19}NO_3+Na]^+$: 320.1257, found: 320.1252.

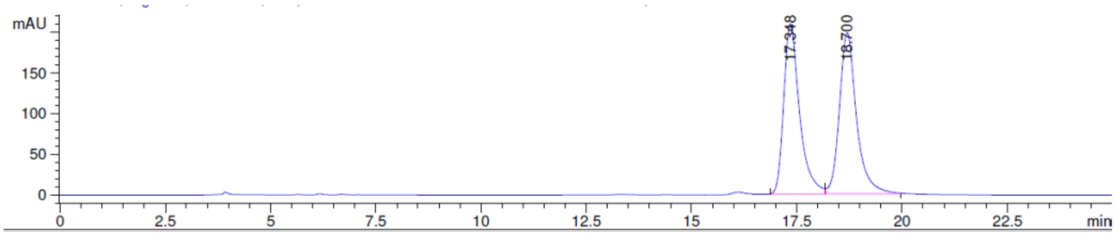

Peak #	RetTime [min]	Type	Width [min]	Area [mAU*s]	Height [mAU]	Area %
1	17.348	BV	0.3890	5466.28613	209.76419	49.3328
2	18.700	VB	0.4235	5614.13965	198.20087	50.6672

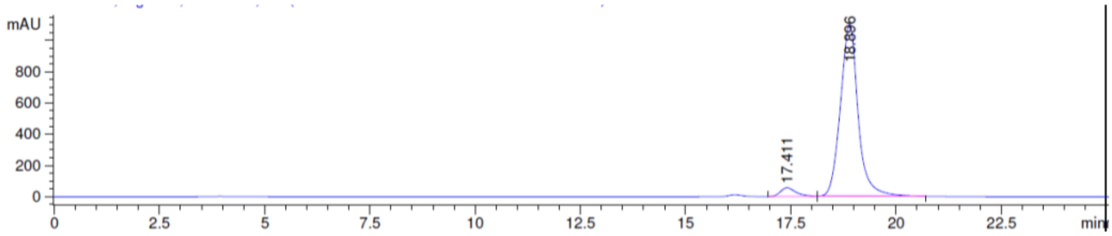

Peak #	RetTime [min]	Type	Width [min]	Area [mAU*s]	Height [mAU]	Area %
1	17.411	BV	0.3890	1426.98560	55.49067	4.2337
2	18.896	VB	0.4417	3.22788e4	1102.11841	95.7663

3-(4-Methoxybenzyl)-4-(*m*-tolyl)oxazolidin-2-one (2g)

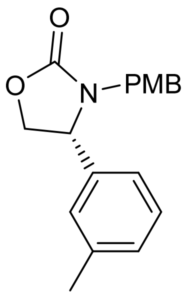

Colorless oil; cyclohexane/THF = 2 mL/0.1 mL, 0 °C, 95% yield, 91% *ee*. $[\alpha]_D^{22}$ = –85.3 (c = 2.44 in $CHCl_3$). HPLC DAICEL CHIRALCEL AS-H, *n*-hexane/2-propanol = 80/20, flow rate = 1 mL/min, λ = 230 nm, retention time: 10.2 min (minor), 13.2 min (major). ^{1}H NMR (600 MHz, $CDCl_3$) δ = 7.29 (t, J = 7.6, 1H), 7.19 (d, J = 7.6, 1H), 7.09 – 7.03 (m, 3H), 7.01 (d, J = 7.6, 1H), 6.82 (t, J = 5.7, 2H), 4.78 (d, J = 14.8, 1H), 4.54 – 4.44 (m, 2H), 4.08 (dd, J = 7.9, 6.5, 1H), 3.79 (s, 3H), 3.61 (d, J = 14.8, 1H), 2.37 (s, 3H). ^{13}C NMR (151 MHz, $CDCl_3$) δ = 159.3, 158.3, 139.2, 137.5, 130.0, 129.8, 129.1, 127.6, 127.5, 124.4, 114.0, 69.9, 58.6, 55.2, 45.2, 21.4. ESI-MS: calculated $[C_{18}H_{19}NO_3+Na]^+$: 320.1257, found: 320.1258.

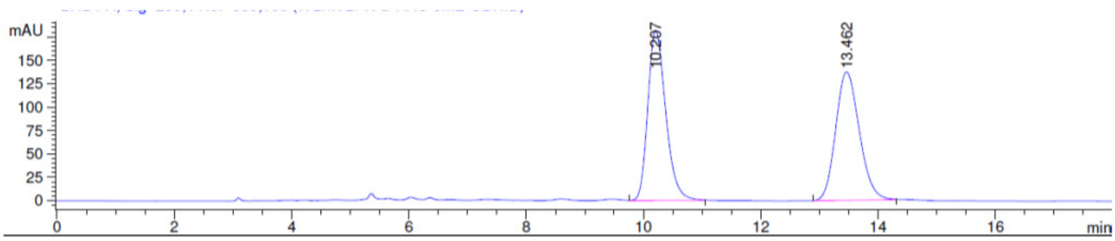

Peak #	RetTime [min]	Type	Width [min]	Area [mAU*s]	Height [mAU]	Area %
1	10.207	VB	0.3264	3858.64575	181.99916	50.0847
2	13.462	BB	0.4341	3845.59229	137.16020	49.9153

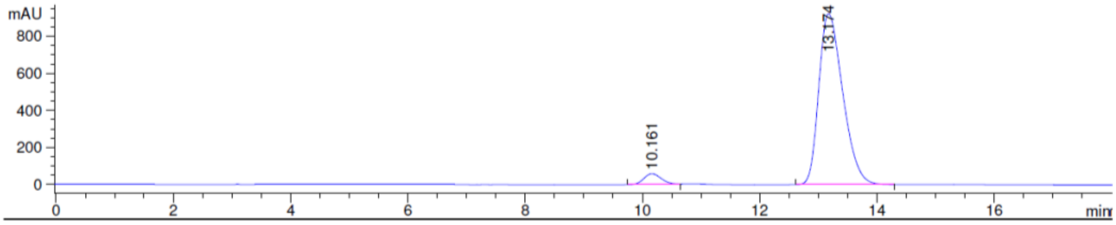

Peak #	RetTime [min]	Type	Width [min]	Area [mAU*s]	Height [mAU]	Area %
1	10.161	BV	0.3275	1253.89136	58.85909	4.5794
2	13.174	BB	0.4407	2.61275e4	921.61096	95.4206

3-(4-Methoxybenzyl)-4-(*p*-tolyl)oxazolidin-2-one (2h)

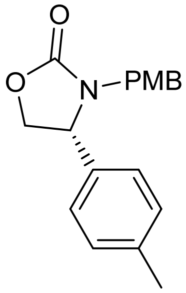

Colorless oil; cyclohexane/THF = 2.0 mL/0.1 mL, 0 °C, 99% yield, 95% *ee*. $[\alpha]_D^{22}$ = –114.6 (c = 1.82 in $CHCl_3$). HPLC DAICEL CHIRALCEL AS-H, *n*-hexane/2-propanol = 80/20, flow rate = 1 mL/min, λ = 254 nm, retention time: 23.4 min (major), 29.6 min (minor). ^{1}H NMR (400 MHz, $CDCl_3$) δ = 7.22 (d, J = 7.8, 2H), 7.12 (d, J = 8.1, 2H), 7.08 – 7.02 (m, 2H), 6.86 – 6.80 (m, 2H), 4.79 (d, J = 14.7, 1H), 4.49 (qd, J = 8.9, 6.8, 2H), 4.07 (dd, J =7 .4, 6.1, 1H), 3.80 (s, 3H), 3.56 (d, J = 14.7, 1H), 2.39 (s, 3H). ^{13}C NMR (101 MHz, $CDCl_3$) δ = 159.3, 158.4, 139.0, 134.4, 130.1, 130.0, 127.5, 127.3, 114.0, 70.0, 58.4, 55.3, 45.1, 21.2. ESI-MS: calculated $[C_{18}H_{19}NO_3+Na]^+$: 320.1257, found: 320.1269.

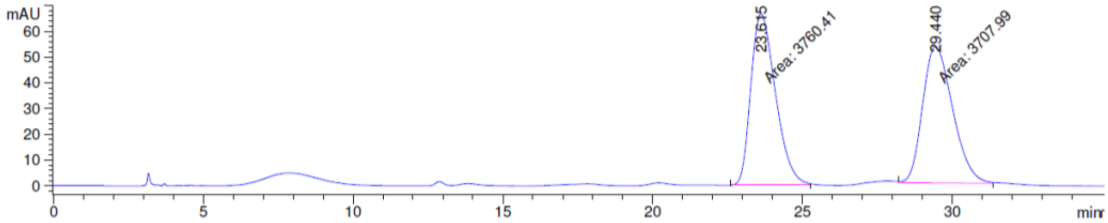

Peak #	RetTime [min]	Type	Width [min]	Area [mAU*s]	Height [mAU]	Area %
1	23.615	MM	0.9470	3760.41235	66.18448	50.3509
2	29.440	MM	1.1636	3707.99365	53.11205	49.6491

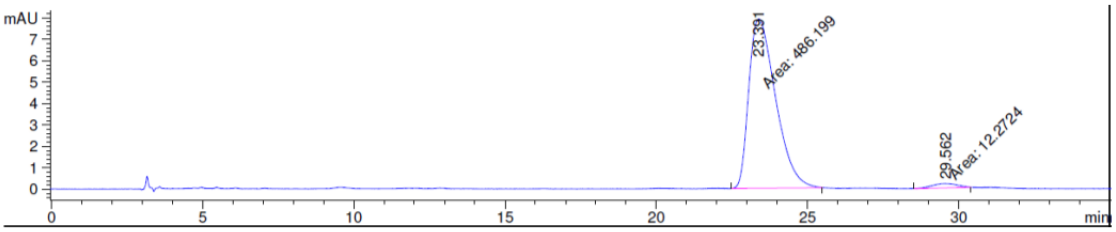

Peak #	RetTime [min]	Type	Width [min]	Area [mAU*s]	Height [mAU]	Area %
1	23.391	MM	1.0270	486.19885	7.89058	97.5380
2	29.562	MM	0.9702	12.27237	2.10827e-1	2.4620

3-(4-Methoxybenzyl)-4-(4-methoxyphenyl)oxazolidin-2-one (2i)

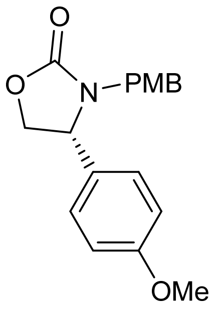

Colorless oil; cyclohexane/THF = 1.0 mL/1.0 mL, 0 °C, 95% yield, 93% *ee*. $[\alpha]_D^{22}$ = –161.6 (c = 0.78 in $CHCl_3$). HPLC DAICEL CHIRALCEL AS-H, *n*-hexane/2-propanol = 70/30, flow rate = 1 mL/min, λ = 230 nm, retention time: 23.6 min (major), 32.3 min (minor). ^1H NMR (400 MHz, $CDCl_3$) δ = 7.17 – 7.11 (m, 2H), 7.05 (dd, J = 9.1, 2.3, 2H), 6.96 – 6.88 (m, 2H), 6.86 – 6.75 (m, 2H), 4.77 (d, J = 14.7, 1H), 4.53 – 4.41 (m, 2H), 4.07 (dd, J = 7.5, 6.4, 1H), 3.83 (s, 3H), 3.80 (s, 3H), 3.56 (d, J = 14.7, 1H). ^{13}C NMR (101 MHz, $CDCl_3$) δ = 160.1, 159.3, 158.3, 130.1, 129.2, 128.6, 127.5, 114.7, 114.0, 70.0, 58.2, 55.4, 55.3, 45.1. ESI-MS: calculated $[C_{18}H_{19}NO_4+Na]^+$: 336.1206, found: 336.1206.

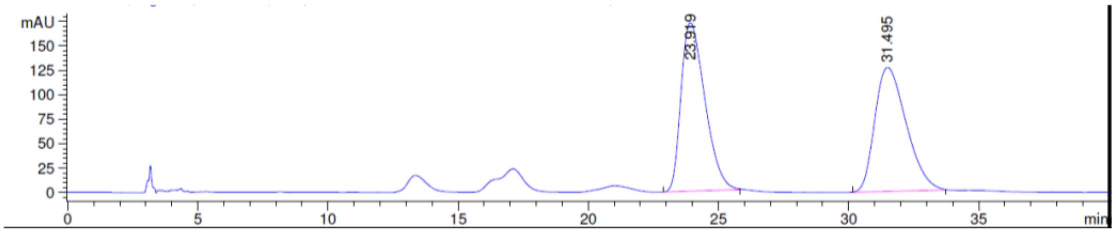

Peak #	RetTime [min]	Type	Width [min]	Area [mAU*s]	Height [mAU]	Area %
1	23.919	BB	0.8894	1.09731e4	172.20703	50.7804
2	31.495	BB	0.9960	1.06358e4	126.75195	49.2196

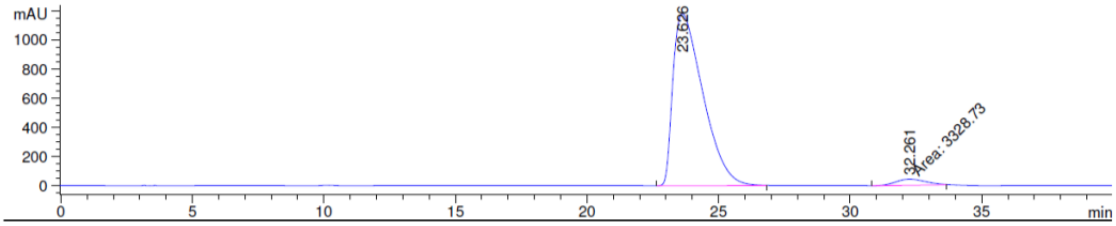

Peak #	RetTime [min]	Type	Width [min]	Area [mAU*s]	Height [mAU]	Area %
1	23.626	BB	1.1426	9.35310e4	1180.15527	96.5634
2	32.261	MM	0.9174	3328.72803	42.47741	3.4366

3-(4-Methoxybenzyl)-4-(4-(trifluoromethyl)phenyl)oxazolidin-2-one (2j)

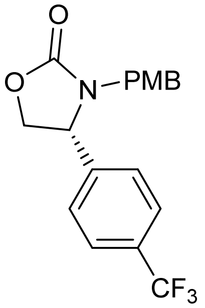

Colorless solid; cyclohexane/THF = 2.0 mL/0.1 mL, 0 °C, 98% yield, 96% *ee*. $[\alpha]_D^{22}$ = –286.6 (c = 0.63 in $CHCl_3$). HPLC DAICEL CHIRALCEL OD-H, *n*-hexane/2-propanol = 80/20, flow rate = 1 mL/min, λ = 254 nm, retention time: 11.6 min (minor), 14.7 min (major). ^{1}H NMR (300 MHz, $CDCl_3$) δ = 7.68 (d, J = 8.1, 2H), 7.36 (d, J = 8.0, 2H), 7.08 – 6.96 (m, 2H), 6.87 – 6.72 (m, 2H), 4.82 (d, J = 14.7, 1H), 4.61 – 4.52 (m, 2H), 4.06 (q, J = 11.3, 1H), 3.79 (s, 3H), 3.61 (d, J = 14.7, 1H). ^{13}C NMR (75 MHz, $CDCl_3$) δ = 159.6, 158.3, 141.8, 131,5 (q, J = 32.7 Hz), 130.2, 127.8, 127.1, 126.5 (q, J = 3.7 Hz), 123.9 (q, J = 272.9 Hz) 114.3, 69.6, 58.3, 55.4, 45.7. ^{19}F NMR (282 MHz, $CDCl_3$) δ = –62.70. ESI-MS: calculated $[C_{18}H_{16}F_3NO_3+Na]^+$: 374.0974, found: 374.0980.

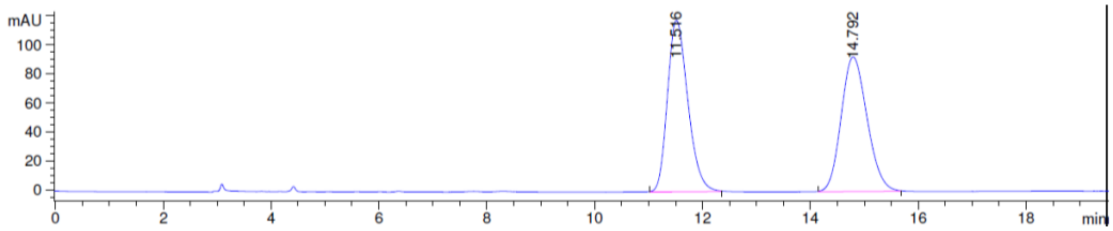

Peak #	RetTime [min]	Type	Width [min]	Area [mAU*s]	Height [mAU]	Area %
1	11.516	BB	0.4082	3099.51538	118.10490	50.0639
2	14.792	BB	0.5183	3091.60010	92.65806	49.9361

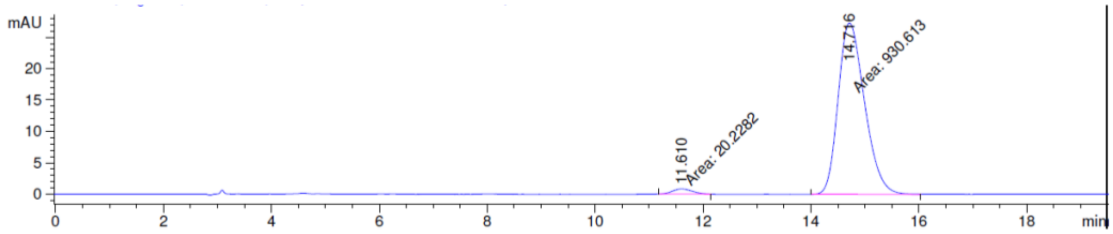

Peak #	RetTime [min]	Type	Width [min]	Area [mAU*s]	Height [mAU]	Area %
1	11.610	MM	0.4191	20.22822	8.04394e-1	2.1274
2	14.716	MM	0.5676	930.61310	27.32662	97.8726

4-(4-Fluorophenyl)-3-(4-methoxybenzyl)oxazolidin-2-one (2k)

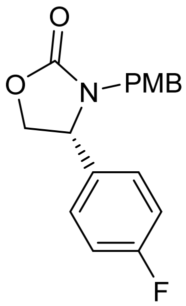

Colorless oil; cyclohexane/THF = 2.0 mL/0.1 mL, 0 °C, 98% yield, 96% *ee*. $[\alpha]_D^{22}$ = –77.4 (c = 0.97 in $CHCl_3$). HPLC DAICEL CHIRALCEL AD-H, *n*-hexane/2-propanol = 90/10, flow rate = 1 mL/min, λ = 230 nm, retention time: 17.6 min (minor), 18.8 min (major). ^{1}H NMR (300 MHz, $CDCl_3$) δ = 7.21 (ddd, *J* = 10.3, 5.2, 2.5, 2H), 7.15 – 7.07 (m, 2H), 7.06 – 6.99 (m, 2H), 6.89 – 6.72 (m, 2H), 4.79 (d, *J* = 14.7, 1H), 4.51 (p, *J* = 8.9, 2H), 4.15 – 3.99 (m, 1H), 3.80 (s, 3H), 3.57 (d, *J* = 14.7, 1H). ^{13}C NMR (75 MHz, $CDCl_3$) δ = 163.1 (d, *J* = 248.3 Hz), 159.5, 158.3, 133.3 (d, *J* = 3.2 Hz), 130.1, 129.2 (d, *J* = 8.4 Hz), 127.3, 116.5 (d, *J* = 21.8 Hz), 114.2, 70.0, 58.1, 55.4, 45.4. ^{19}F NMR (282 MHz, $CDCl_3$) δ = –112.22. ESI-MS: calculated $[C_{17}H_{16}FNO_3+Na]^+$: 324.1006, found: 324.1003.

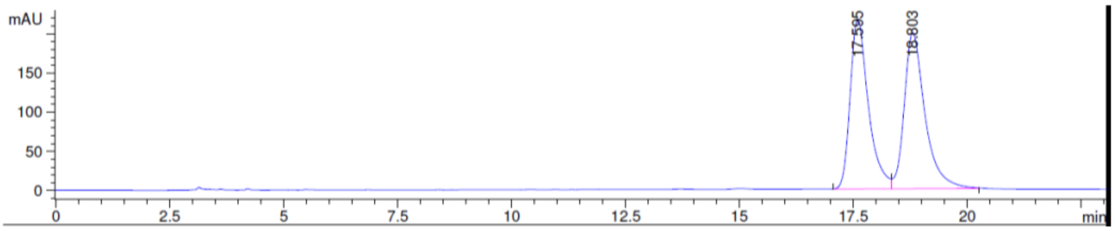

Peak #	RetTime [min]	Type	Width [min]	Area [mAU*s]	Height [mAU]	Area %
1	17.595	BV	0.4172	6029.98096	216.97952	48.8420
2	18.803	VB	0.4755	6315.90820	198.88145	51.1580

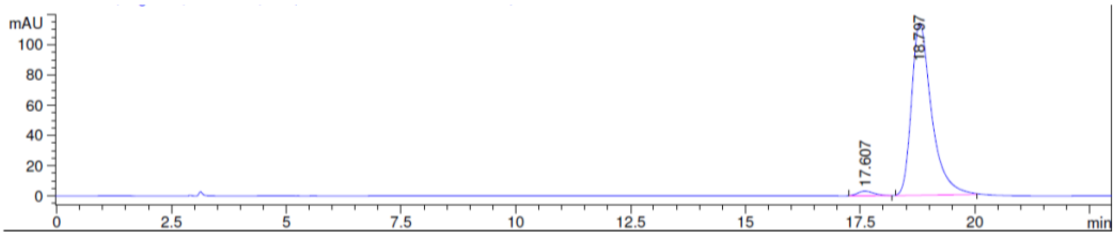

Peak #	RetTime [min]	Type	Width [min]	Area [mAU*s]	Height [mAU]	Area %
1	17.607	BB	0.3638	74.46832	3.07258	2.1049
2	18.797	BB	0.4549	3463.40381	113.85394	97.8951

4-(4-Chlorophenyl)-3-(4-methoxybenzyl)oxazolidin-2-one (2l)

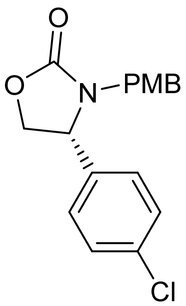

Colorless oil; cyclohexane/THF = 1.0 mL/1.0 mL, 0 °C, 97% yield, 94% *ee*. $[\alpha]_D^{22}$ = –121.2 (c = 1.30 in $CHCl_3$). HPLC DAICEL CHIRALCEL OD-H, *n*-hexane/2-propanol = 80/20, flow rate = 1 mL/min, λ = 230 nm, retention time: 13.3 min (minor), 16.8 min (major). ^1H NMR (400 MHz, $CDCl_3$) δ = 7.45 – 7.33 (m, 2H), 7.22 – 7.11 (m, 2H), 7.09 – 6.97 (m, 2H), 6.87 – 6.75 (m, 2H), 4.80 (d, J = 14.7, 1H), 4.50 (qd, J = 8.9, 7.0, 2H), 4.04 (dd, J = 7.7, 6.1, 1H), 3.79 (s, 3H), 3.57 (d, J = 14.7, 1H). ^{13}C NMR (101 MHz, $CDCl_3$) δ = 159.4, 158.2, 136.0, 135.0, 130.1, 129.6, 128.7, 127.1, 114.1, 69.7, 58.1, 55.3, 45.4. ESI-MS: calculated $[C_{17}H_{16}ClNO_3+Na]^+$: 340.0711, found: 340.0718.

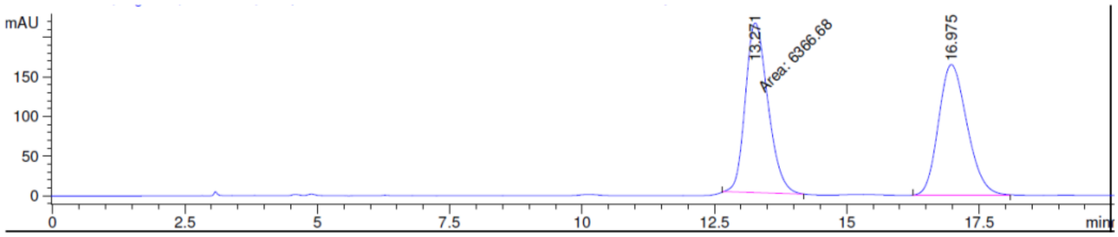

Peak #	RetTime [min]	Type	Width [min]	Area [mAU*s]	Height [mAU]	Area %
1	13.271	MM	0.4950	6366.68457	214.35805	50.6986
2	16.975	BB	0.5853	6191.22803	164.79747	49.3014

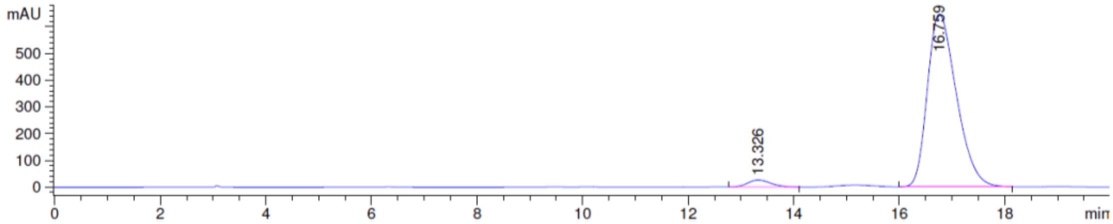

Peak #	RetTime [min]	Type	Width [min]	Area [mAU*s]	Height [mAU]	Area %
1	13.326	BB	0.4581	771.47156	25.77944	3.0324
2	16.759	VB	0.5901	2.46699e4	646.65814	96.9676

4-(4-Bromophenyl)-3-(4-methoxybenzyl)oxazolidin-2-one (2m)

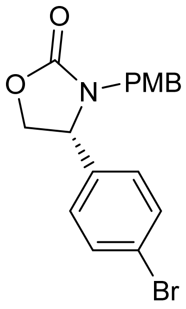

Colorless oil; cyclohexane/THF = 1.0 mL/1.0 mL, 0 °C, 99% yield, 94% *ee*. $[\alpha]_D^{22}$ = –138.8 (c = 2.15 in $CHCl_3$). HPLC DAICEL CHIRALCEL OD-H, *n*-hexane/2-propanol = 80/20, flow rate = 1 mL/min, λ = 230 nm, retention time: 14.7 min (minor), 17.9 min (major). ^1H NMR (300 MHz, $CDCl_3$) δ = 7.58 – 7.48 (m, 2H), 7.16 – 7.07 (m, 2H), 7.06 – 6.94 (m, 2H), 6.87 – 6.74 (m, 2H), 4.80 (d, J = 14.7, 1H), 4.49 (qd, J = 8.9, 7.0, 2H), 4.04 (dd, J = 7.7, 6.2, 1H), 3.80 (s, 3H), 3.57 (d, J = 14.7, 1H). ^{13}C NMR (75 MHz, $CDCl_3$) δ = 159.4, 158.2, 136.6, 132.5, 130.1, 129.0, 127.1, 123.1, 114.1, 69.6, 58.1, 55.3, 45.4. ESI-MS: calculated $[C_{17}H_{16}BrNO_3+Na]^+$: 384.0206, 386.0185, found: 384.0206, 386.0188.

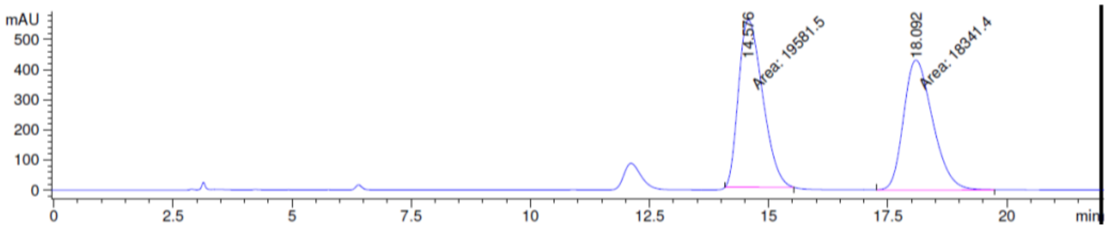

Peak #	RetTime [min]	Type	Width [min]	Area [mAU*s]	Height [mAU]	Area %
1	14.576	MM	0.5876	1.95815e4	555.38367	51.6350
2	18.092	MM	0.7087	1.83414e4	431.35019	48.3650

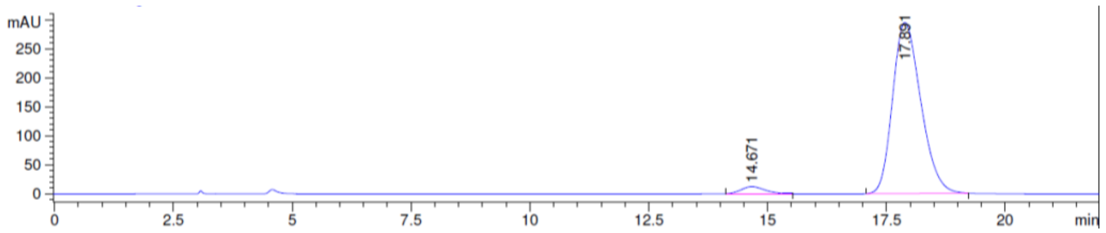

Peak #	RetTime [min]	Type	Width [min]	Area [mAU*s]	Height [mAU]	Area %
1	14.671	BB	0.5013	405.49939	11.99930	3.2271
2	17.891	BB	0.6399	1.21597e4	295.01819	96.7729

4-([1,1'-Biphenyl]-4-yl)-3-(4-methoxybenzyl)oxazolidin-2-one (2n)

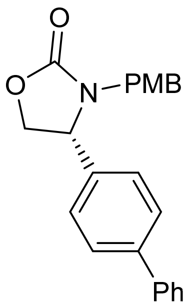

Colorless solid; cyclohexane/THF = 1.0 mL/1.0 mL, 0 °C, 99% yield, 93% *ee*. $[\alpha]_D^{22}$ = –193.5 (c = 0.64 in $CHCl_3$). HPLC DAICEL CHIRALCEL AS-H, *n*-hexane/2-propanol = 70/30, flow rate = 1 mL/min, λ = 230 nm, retention time: 19.2 min (major), 28.6 min (minor). ^{1}H NMR (300 MHz, $CDCl_3$) δ = 7.70 – 7.56 (m, 4H), 7.53 – 7.43 (m, 2H), 7.40 (dt, *J* = 9.6, 4.3, 1H), 7.35 – 7.27 (m, 2H), 7.15 – 7.04 (m, 2H), 6.89 – 6.80 (m, 2H), 4.83 (d, *J* = 14.7, 1H), 4.61 – 4.48 (m, 2H), 4.14 (q, *J* = 11.6, 1H), 3.80 (s, 3H), 3.65 (d, *J* = 14.7, 1H). ^{13}C NMR (75 MHz, $CDCl_3$) δ = 159.4, 158.4, 142.1, 140.2, 136.4, 130.2, 129.0, 128.0, 127.8, 127.4, 127.1, 114.1, 69.9, 58.4, 55.3, 45.3. ESI-MS: calculated $[C_{23}H_{21}NO_3+Na]^+$: 382.1414, found: 382.1404.

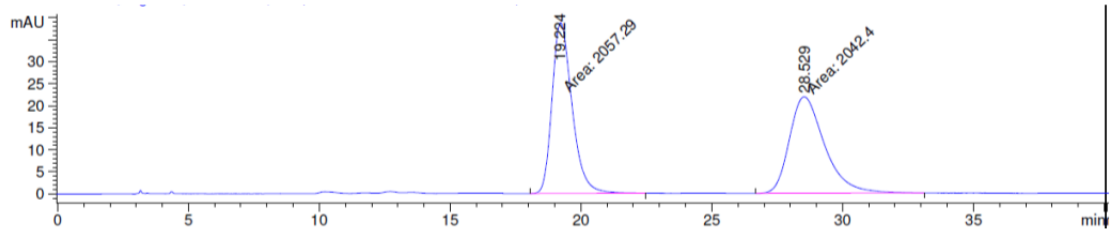

Peak #	RetTime [min]	Type	Width [min]	Area [mAU*s]	Height [mAU]	Area %
1	19.224	MM	0.8852	2057.29053	38.73398	50.1816
2	28.529	MM	1.5569	2042.39709	21.86439	49.8184

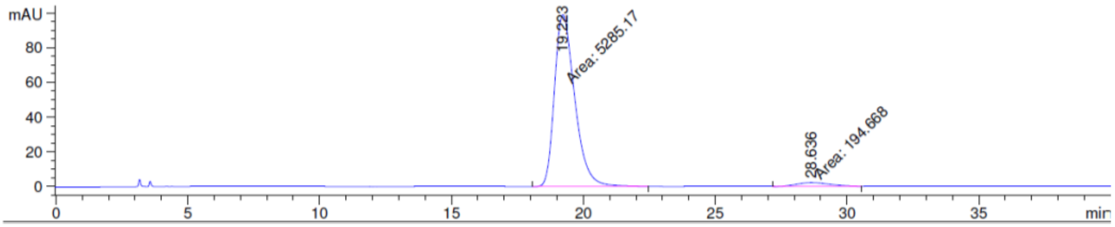

Peak #	RetTime [min]	Type	Width [min]	Area [mAU*s]	Height [mAU]	Area %
1	19.223	MM	0.8903	5285.17188	98.93953	96.4476
2	28.636	MM	1.4772	194.66768	2.19632	3.5524

3-(4-Methoxybenzyl)-4-(4-(methylthio)phenyl)oxazolidin-2-one (2o)

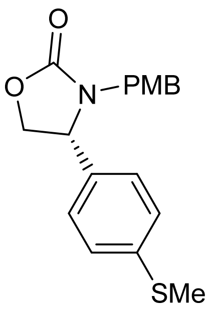

Colorless oil; cyclohexane/THF = 2.0 mL/0.1 mL, 0 °C, 99% yield, 93% *ee*. $[\alpha]_D^{22}$ = –179.7 (c = 2.10 in $CHCl_3$). HPLC DAICEL CHIRALCEL OD-H, *n*-hexane/2-propanol = 80/20, flow rate = 1 mL/min, λ = 254 nm, retention time: 17.1 min (minor), 20.0 min (major). ^1H NMR (300 MHz, $CDCl_3$) δ = 7.30 (dd, J = 7.0, 1.3, 2H), 7.22 – 7.12 (m, 2H), 7.12 – 7.00 (m, 2H), 6.90 – 6.78 (m, 2H), 4.82 (d, J = 14.7, 1H), 4.59 – 4.44 (m, 2H), 4.08 (dd, J = 7.3, 6.1, 1H), 3.82 (s, 3H), 3.60 (d, J = 14.7, 1H), 2.54 (s, 3H). ^{13}C NMR (75 MHz, $CDCl_3$) δ = 159.3, 158.3, 140.0, 133.9, 130.1, 127.8, 127.4, 126.8, 114.1, 69.8, 58.2, 55.3, 45.2, 15.5. ESI-MS: calculated $[C_{18}H_{19}NO_3S+Na]^+$: 352.0978, found: 352.0982.

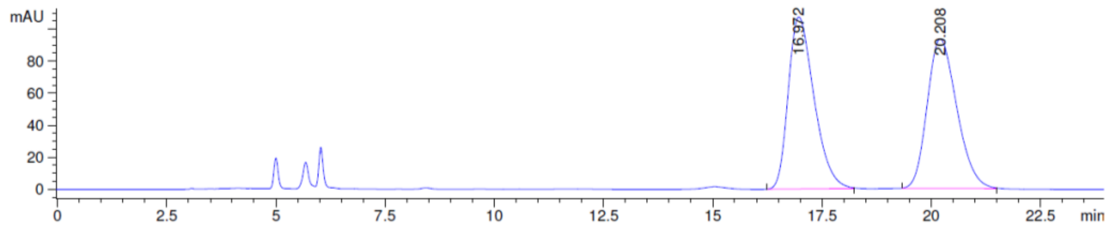

Peak #	RetTime [min]	Type	Width [min]	Area [mAU*s]	Height [mAU]	Area %
1	16.972	BB	0.6371	4405.09912	107.27018	50.1193
2	20.208	BB	0.7246	4384.12891	93.23904	49.8807

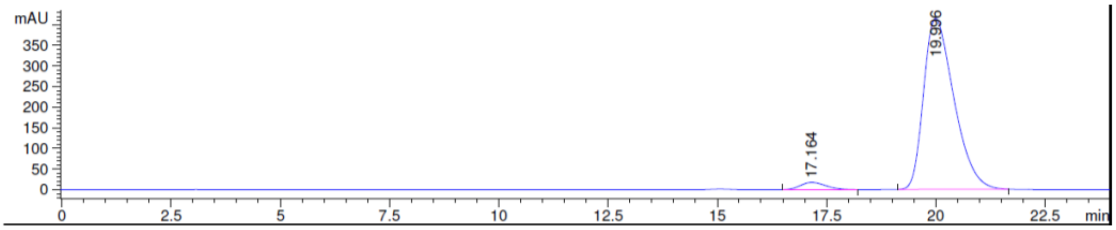

Peak #	RetTime [min]	Type	Width [min]	Area [mAU*s]	Height [mAU]	Area %
1	17.164	BB	0.5816	703.66150	17.29635	3.4004
2	19.996	BB	0.7440	1.99895e4	414.23303	96.5996

4-(Benzo[*d*][1,3]dioxol-5-yl)-3-(4-methoxybenzyl)oxazolidin-2-one (2p)

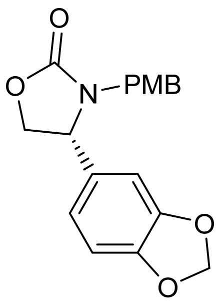

Colorless oil; cyclohexane/THF = 2.0 mL/0.1 mL, 0 °C, 90% yield, 96% *ee*. $[\alpha]_D^{22}$ = –126.3 (c = 1.49 in $CHCl_3$). HPLC DAICEL CHIRALCEL AS-H, *n*-hexane/2-propanol = 70/30, flow rate = 1 mL/min, λ = 230 nm, retention time: 35.8 min (major), 46.9 min (minor). ^{1}H NMR (300 MHz, $CDCl_3$) δ = 7.14 – 7.03 (m, 2H), 6.88 – 6.77 (m, 3H), 6.73 (d, *J* = 1.7, 1H), 6.65 (dd, *J* = 7.9, 1.8, 1H), 6.01 (s, 2H), 4.78 (d, *J* = 14.7, 1H), 4.54 – 4.37 (m, 2H), 4.04 (dd, *J* = 7.8, 6.5, 1H), 3.79 (s, 3H), 3.60 (d, *J* = 14.7, 1H). ^{13}C NMR (75 MHz, $CDCl_3$) δ = 159.3, 158.2, 148.7, 148.3, 131.2, 130.1, 127.4, 121.3, 114.1, 108.6, 106.9, 101.5, 69.9, 58.5, 55.3, 45.1. ESI-MS: calculated $[C_{18}H_{17}NO_5+Na]^+$: 350.0999, found: 350.0995.

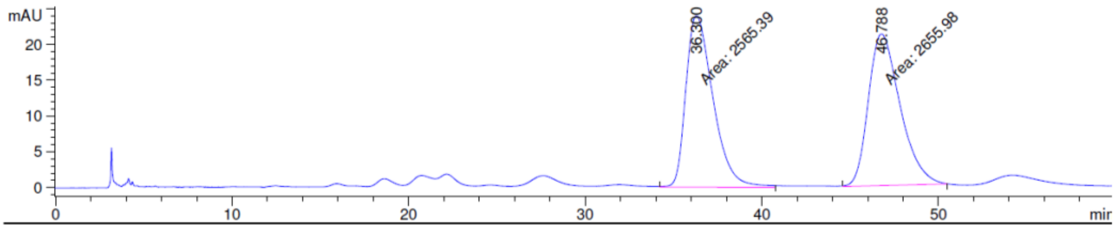

Peak #	RetTime [min]	Type	Width [min]	Area [mAU*s]	Height [mAU]	Area %
1	36.300	MM	1.7835	2565.39209	23.97279	49.1325
2	46.788	MM	2.0877	2655.98120	21.20355	50.8675

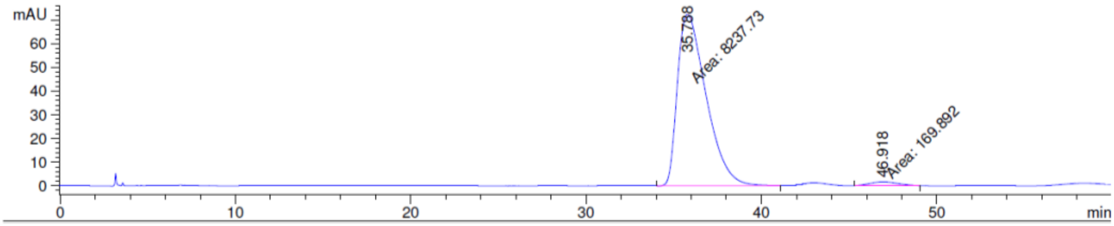

Peak #	RetTime [min]	Type	Width [min]	Area [mAU*s]	Height [mAU]	Area %
1	35.788	MM	1.8952	8237.72949	72.44439	97.9793
2	46.918	MM	1.7846	169.89182	1.58663	2.0207

3-(4-Methoxybenzyl)-4-(4-morpholinophenyl)oxazolidin-2-one (2q)

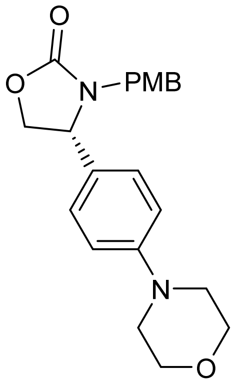

Colorless oil; cyclohexane/THF = 1.0 mL/1.0 mL, 0 °C, 93% yield, 93% *ee*. $[\alpha]_D^{22}$ = –133.0 (c = 1.16 in $CHCl_3$). HPLC DAICEL CHIRALCEL OD-H, *n*-hexane/2-propanol = 80/20, flow rate = 0.8 mL/min, λ = 230 nm, retention time: 43.0 min (minor), 45.8 min (major). ^{1}H NMR (400 MHz, $CDCl_3$) δ = 7.17 – 7.11 (m, 2H), 7.10 – 7.03 (m, 2H), 6.96 – 6.89 (m, 2H), 6.86 – 6.79 (m, 2H), 4.77 (d, J = 14.7 Hz, 1H), 4.55 – 4.38 (m, 2H), 4.07 (dd, J = 7.9, 6.7 Hz, 1H), 3.91 – 3.85 (m, 4H), 3.80 (s, 3H), 3.56 (d, J = 14.7 Hz, 1H), 3.23 – 3.17 (m, 4H). ^{13}C NMR (101 MHz, $CDCl_3$) δ = 159.3, 158.3, 151.6, 130.1,

128.4, 128.0, 127.6, 115.8, 114.0, 70.0, 66.8, 58.2, 55.3, 48.9, 45.0. ESI-MS: calculated $[C_{21}H_{24}N_2O_4+Na]^+$: 391.1628, found: 391.1645.

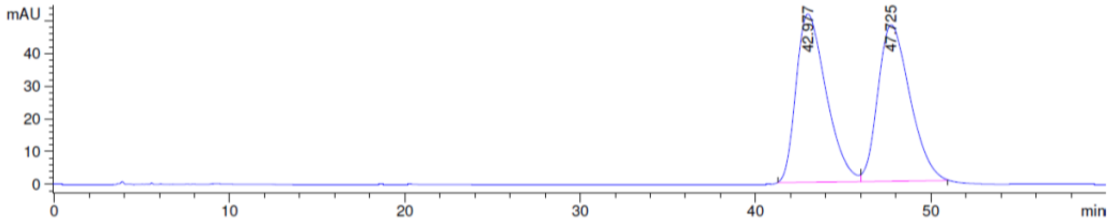

Peak #	RetTime [min]	Type	Width [min]	Area [mAU*s]	Height [mAU]	Area %
1	42.977	BB	1.7654	6143.90723	51.44282	50.2550
2	47.725	BB	1.8454	6081.55176	47.83133	49.7450

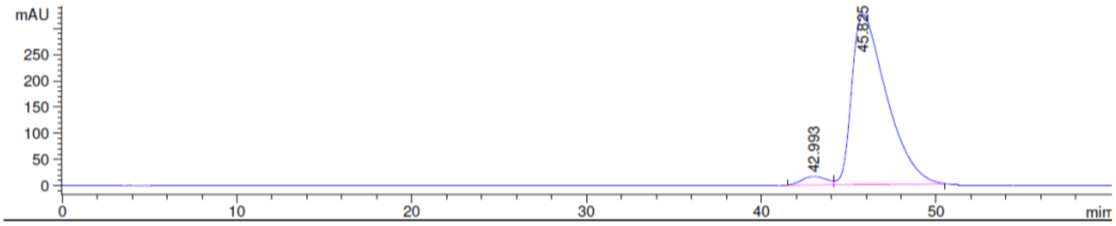

Peak #	RetTime [min]	Type	Width [min]	Area [mAU*s]	Height [mAU]	Area %
1	42.993	BV	1.1759	1611.62671	16.12636	3.4537
2	45.825	VB	1.9292	4.50524e4	324.81747	96.5463

Methyl 4-(3-(4-methoxybenzyl)-2-oxooxazolidin-4-yl)benzoate (2r)

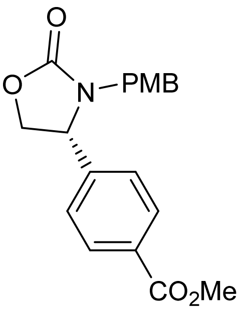

Colorless oil; cyclohexane/THF = 0 mL/2.0 mL, –10 °C, 76% yield, 91% *ee*. $[\alpha]_D^{22}$ = –107.3 (c = 1.36 in $CHCl_3$). HPLC DAICEL CHIRALCEL AS-H, *n*-hexane/2-propanol = 70/30, flow rate = 1 mL/min, λ = 230 nm, retention time: 29.5 min (major), 40.8 min (minor). ^1H NMR (400 MHz, $CDCl_3$) δ = 8.15 – 8.04 (m, 2H), 7.34 – 7.27 (m, 2H), 7.05 – 6.98 (m, 2H), 6.87 – 6.77 (m, 2H), 4.83 (d, *J* = 14.7, 1H), 4.60 – 4.50 (m, 2H), 4.07 (q, *J* = 11.3, 1H), 3.94 (s, 3H), 3.79 (s, 3H), 3.57 (d, *J* = 14.7, 1H). ^{13}C NMR (101 MHz, $CDCl_3$) δ = 166.4, 159.5, 158.2, 142.6,

131.0, 130.6, 130.1, 127.3, 127.0, 114.2, 69.5, 58.3, 55.3, 52.4, 45.5. ESI-MS: calculated $[C_{19}H_{19}NO_5+Na]^+$: 364.1155, found: 364.1165.

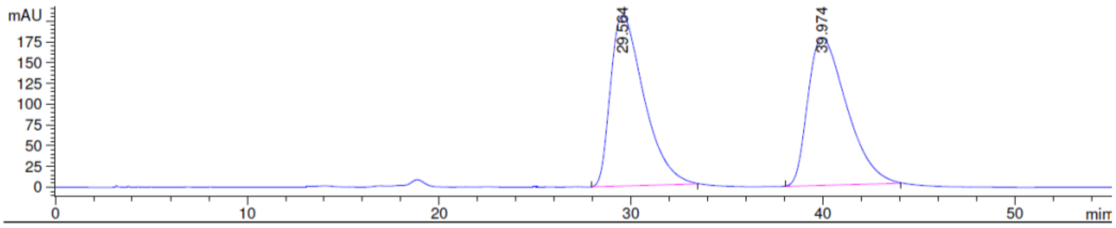

Peak #	RetTime [min]	Type	Width [min]	Area [mAU*s]	Height [mAU]	Area %
1	29.564	BB	1.7522	2.49036e4	205.59198	49.9567
2	39.974	BB	1.9792	2.49468e4	177.55614	50.0433

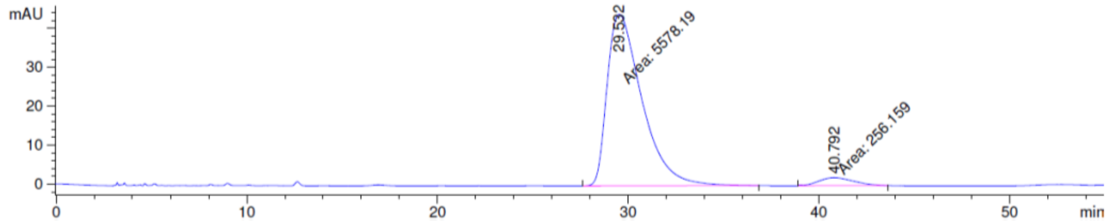

Peak #	RetTime [min]	Type	Width [min]	Area [mAU*s]	Height [mAU]	Area %
1	29.532	MM	2.1126	5578.18896	44.00636	95.6095
2	40.792	MM	2.0927	256.15851	2.04008	4.3905

(*R*)-3-(4-Methoxybenzyl)-4-(4-(methylsulfonyl)phenyl)oxazolidin-2-one (2s)

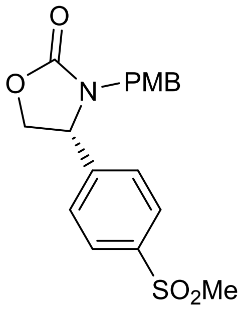

Colorless solid; cyclohexane/THF = 0 mL/2.0 mL, 0 °C, 98% yield, 94% *ee*. $[\alpha]_D^{22}$ = –244.7 (c = 0.41 in $CHCl_3$). HPLC DAICEL CHIRALCEL AD-H, *n*-hexane/2-propanol = 70/30, flow rate = 0.5 mL/min, λ = 230 nm, retention time: 69.0 min (major), 75.0 min (minor). ^{1}H NMR (300 MHz, $CDCl_3$) δ = 8.03 – 7.89 (m, 2H), 7.49 – 7.37 (m, 2H), 7.06 – 6.93 (m, 2H), 6.86 – 6.75 (m, 2H), 4.81 (d, *J* = 14.7, 1H), 4.66 – 4.52 (m, 2H), 4.06 (dd, *J* = 6.3, 4.4, 1H), 3.79 (s, 3H), 3.65 (d, *J* = 14.8, 1H), 3.09 (s, 3H). ^{13}C NMR (75 MHz, $CDCl_3$) δ = 159.5, 158.1, 144.0, 141.4,

130.0, 128.5, 128.2, 126.8, 114.2, 69.3, 58.2, 55.3, 45.8, 44.4. ESI-MS: calculated $[C_{18}H_{19}NO_5S+Na]^+$: 384.0876, found: 384.0873.

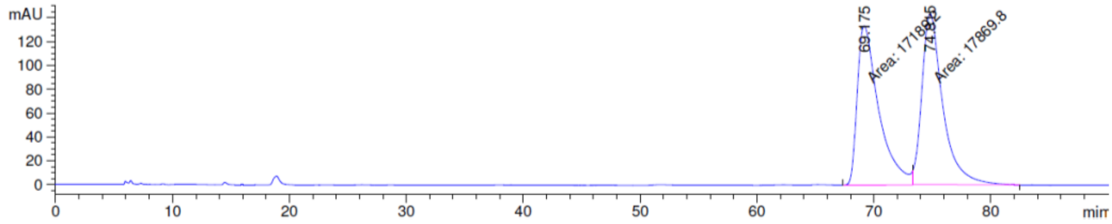

Peak #	RetTime [min]	Type	Width [min]	Area [mAU*s]	Height [mAU]	Area %
1	69.175	MM	2.1433	1.71892e4	133.66618	49.0293
2	74.815	MM	2.0783	1.78698e4	143.30595	50.9707

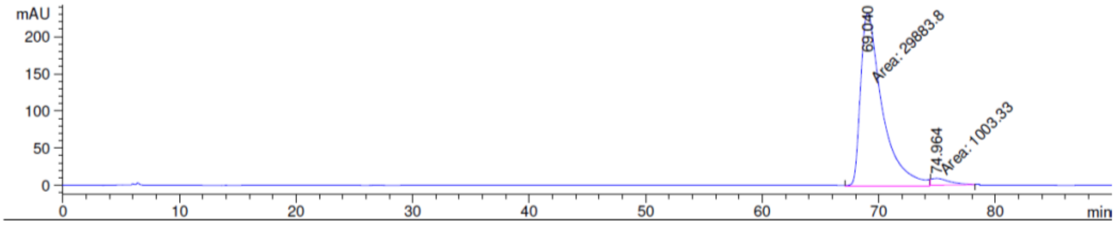

Peak #	RetTime [min]	Type	Width [min]	Area [mAU*s]	Height [mAU]	Area %
1	69.040	MM	2.1515	2.98838e4	231.49097	96.7516
2	74.964	MM	1.8417	1003.33301	9.08001	3.2484

X-Ray diffraction: Data sets were collected with a Nonius Kappa CCD diffractometer. Programs used: data collection, COLLECT (R. W. W. Hooft, Bruker AXS, 2008, Delft, The Netherlands); data reduction Denzo-SMN (Z. Otwinowski, W. Minor, *Methods Enzymol.* **1997**, *276*, 307-326); absorption correction, Denzo (Z. Otwinowski, D. Borek, W. Majewski, W. Minor, *Acta Crystallogr.* **2003**, *A59*, 228-234); structure solution SHELXS-97 (G. M. Sheldrick, *Acta Crystallogr.* **1990**, *A46*, 467-473); structure refinement SHELXL-97 (G. M. Sheldrick, *Acta Crystallogr.* **2008**, *A64*, 112-122) and graphics, XP (BrukerAXS, 2000). *R*-values are given for observed reflections, and wR^2 values are given for all reflections.

X-ray crystal structure analysis of 2s: formula $C_{18}H_{19}NO_5S$, M = 361.40, colourless crystal, 0.16 x 0.06 x 0.03 mm, a = 19.1685(5), b = 5.7871(3), c = 14.8903(3) Å, V = 1651.8(1) Å^3, ρ_{calc} = 1.453 g·cm^{-3}, μ = 0.226 mm^{-1}, empirical absorption correction (0.964 ≤ T ≤ 0.993), Z = 4, orthorhombic, space group $P2_12_12_1$ (No. 19), λ = 0.71073 Å, T = 173(2) K, ω and φ scans, 8858 reflections collected (±h, ±k, ±l), 2765 independent (R_{int} = 0.061) and 2582 observed reflections [I>2σ(I)], 228 refined parameters, R = 0.044, wR^2 = 0.091, max. (min.) residual electron density 0.18 (-0.18) e.Å^{-3}, hydrogen atoms calculated and refined as riding atoms. Flack parameter: -0.03(11).

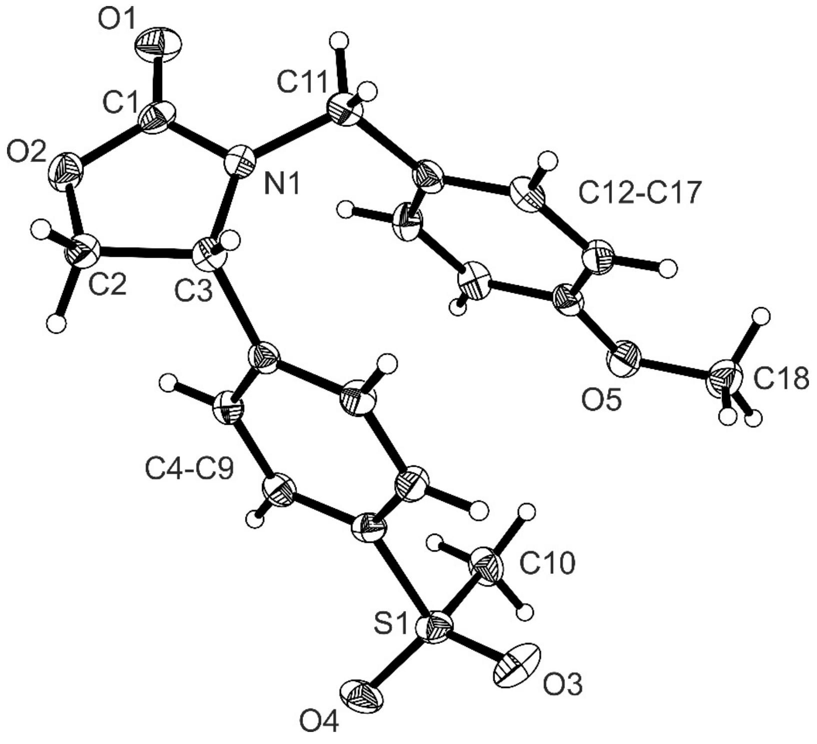

Crystal structure of compound **2s**.

(Thermals ellipsoids are shown with 30% probability.)

3-(4-Methoxybenzyl)-4-(naphthalen-1-yl)oxazolidin-2-one (2t)

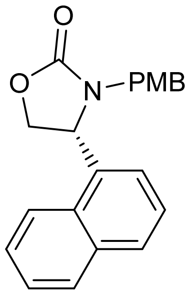

Colorless oil; cyclohexane/THF = 1.0 mL/1.0 mL, 0 °C, 97% yield, 95% *ee*. $[\alpha]_D^{22}$ = –126.1 (c = 1.37 in $CHCl_3$). HPLC DAICEL CHIRALCEL AS-H, *n*-hexane/2-propanol = 70/30, flow rate = 1 mL/min, λ = 230 nm, retention time: 21.8 min (major), 27.0 min (minor). ^{1}H NMR (400 MHz, $CDCl_3$) δ = 7.98 – 7.84 (m, 2H), 7.74 – 7.32 (m, 5H), 7.02 (d, J = 8.6, 2H), 6.78 (d, J = 8.6, 2H), 5.41 (s, 1H), 5.06 – 4.81 (m, 1H), 4.69 (s, 1H), 4.05 (s, 1H), 3.87 – 3.72 (m, 4H). ^{13}C NMR (101 MHz, $CDCl_3$) δ = 159.5, 158.8, 134.2, 133.5, 130.8, 130.2, 129.4, 129.1, 127.6, 127.0, 126.3, 125.9, 123.3, 121.7, 114.2, 70.1, 55.4, 53.7, 45.8. ESI-MS: calculated $[C_{21}H_{19}NO_3+Na]^+$: 356.1257, found: 356.1266.

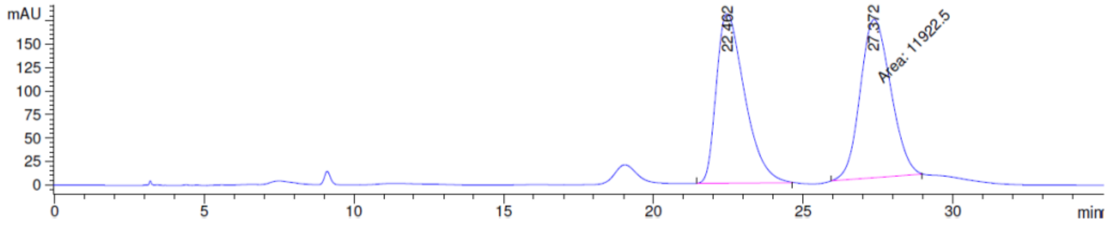

Peak #	RetTime [min]	Type	Width [min]	Area [mAU*s]	Height [mAU]	Area %
1	22.462	BB	0.9836	1.18630e4	180.29207	49.8749
2	27.372	MM	1.1800	1.19225e4	168.39098	50.1251

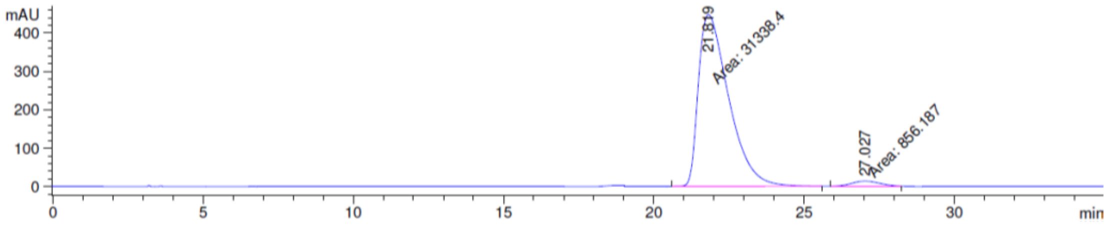

Peak #	RetTime [min]	Type	Width [min]	Area [mAU*s]	Height [mAU]	Area %
1	21.819	MM	1.1678	3.13384e4	447.27002	97.3406
2	27.027	MM	1.0713	856.18726	13.31951	2.6594

3-(4-Methoxybenzyl)-4-(naphthalen-2-yl)oxazolidin-2-one (2u)

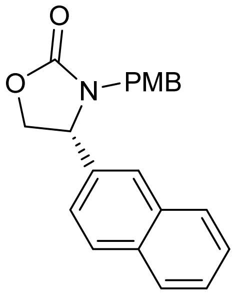

Colorless solid; cyclohexane/THF = 1.0 mL/1.0 mL, 0 °C, 98% yield, 94% *ee*. $[\alpha]_D^{22}$ = –175.0 (c = 1.54 in $CHCl_3$). HPLC DAICEL CHIRALCEL AS-H, *n*-hexane/2-propanol = 80/20, flow rate = 1 mL/min, λ = 230 nm, retention time: 33.0 min (major), 44.2 min (minor). ^{1}H NMR (300 MHz, $CDCl_3$) δ = 8.00 – 7.80 (m, 3H), 7.65 (s, 1H), 7.60 – 7.51 (m, 2H), 7.38 (dd, J = 8.5, 1.8, 1H), 7.10 – 6.99 (m, 2H), 6.88 – 6.77 (m, 2H), 4.85 (d, J = 14.7, 1H), 4.69 (dd, J = 8.9, 6.9, 1H), 4.60 (t, J = 8.7, 1H), 4.19 (dd, J = 8.4, 6.9, 1H), 3.80 (s, 3H), 3.62 (d, J = 14.7, 1H). ^{13}C NMR (75 MHz, $CDCl_3$) δ = 159.4, 158.4, 134.7, 133.6, 133.2, 130.1, 129.7, 127.9, 127.9, 127.4, 127.2, 126.9, 126.8 123.8, 114.1, 69.7, 58.8, 55.3, 45.3. ESI-MS: calculated $[C_{21}H_{19}NO_3+Na]^+$: 356.1257, found: 356.1244.

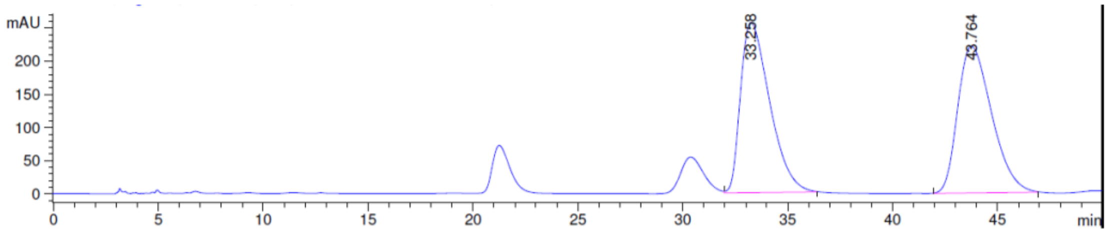

Peak #	RetTime [min]	Type	Width [min]	Area [mAU*s]	Height [mAU]	Area %
1	33.258	VB	1.4276	2.47880e4	256.46979	49.9385
2	43.764	BB	1.6666	2.48491e4	221.02969	50.0615

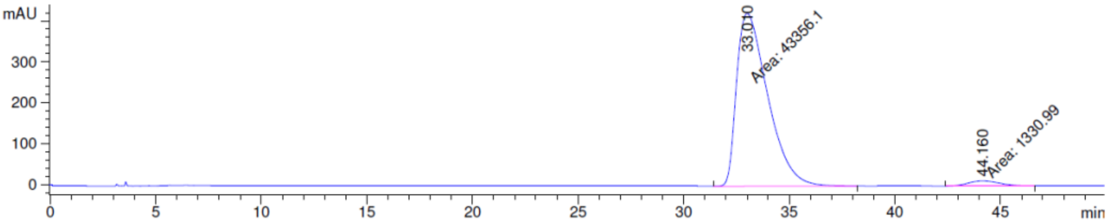

Peak #	RetTime [min]	Type	Width [min]	Area [mAU*s]	Height [mAU]	Area %
1	33.010	MM	1.7095	4.33561e4	422.69315	97.0215
2	44.160	MM	1.7736	1330.99268	12.50751	2.9785

3-(4-Methoxybenzyl)-4-(thiophen-3-yl)oxazolidin-2-one (2v)

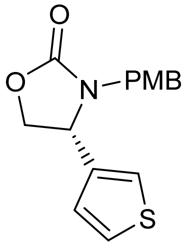

Colorless solid; cyclohexane/THF = 1.0 mL/1.0 mL, 0 °C, 97% yield, 93% *ee*. $[\alpha]_D^{22}$ = –65.5 (c = 1.01 in $CHCl_3$). HPLC DAICEL CHIRALCEL AS-H, *n*-hexane/2-propanol = 70/30, flow rate = 1 mL/min, λ = 210 nm, retention time: 23.1 min (major), 29.0 min (minor). ^{1}H NMR (400 MHz, $CDCl_3$) δ = 7.41 (dd, J = 5.0, 2.9, 1H), 7.17 (dd, J = 2.9, 1.3, 1H), 7.11 – 7.05 (m, 2H), 7.02 (dd, J = 5.0, 1.3, 1H), 6.86 – 6.80 (m, 2H), 4.76 (d, J = 14.7, 1H), 4.66 (dd, J = 8.7, 7.3, 1H), 4.49 (t, J = 8.7, 1H), 4.13 (dd, J = 8.7, 7.2, 1H), 3.80 (s, 3H), 3.63 (d, J = 14.7, 1H). ^{13}C NMR (101 MHz, $CDCl_3$) δ = 159.4, 158.1, 138.8, 130.1, 128.0, 127.6, 125.6, 124.4, 114.2, 69.2, 55.4, 54.3, 45.4. ESI-MS: calculated $[C_{15}H_{15}NO_3S+Na]^+$: 312.0665, found: 312.0673

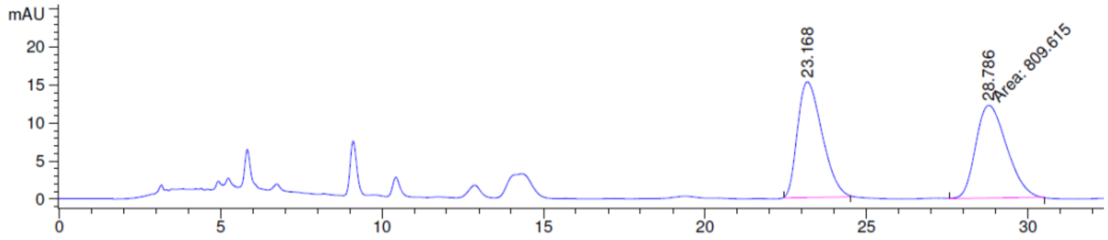

Peak #	RetTime [min]	Type	Width [min]	Area [mAU*s]	Height [mAU]	Area %
1	23.168	BB	0.7726	807.91760	15.18801	49.9475
2	28.786	MM	1.1089	809.61511	12.16860	50.0525

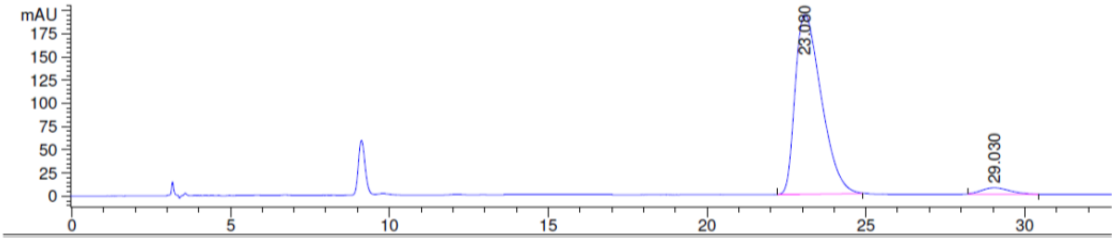

Peak #	RetTime [min]	Type	Width [min]	Area [mAU*s]	Height [mAU]	Area %
1	23.080	BB	0.8127	1.10693e4	193.40445	96.4560
2	29.030	BB	0.7148	406.70673	6.69444	3.5440

3-(4-Methoxybenzyl)-4-(pyridin-3-yl)oxazolidin-2-one (2w)

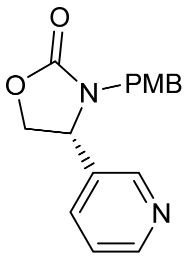

Colorless oil; cyclohexane/THF = 0 mL/2.0 mL, 0 °C, 98% yield, 94% *ee*. $[\alpha]_D^{22}$ = –90.0 (c = 0.25 in $CHCl_3$). HPLC DAICEL CHIRALCEL AS-H, *n*-hexane/2-propanol = 70/30, flow rate = 1 mL/min, λ = 230 nm, retention time: 29.8 min (major), 40.8 min (minor). ^{1}H NMR (600 MHz, $CDCl_3$) δ = 8.64 (dd, J = 4.8, 1.6, 1H), 8.42 (d, J = 1.9, 1H), 7.65 – 7.56 (m, 1H), 7.37 (dd, J = 7.9, 4.8, 1H), 7.02 (d, J = 8.6, 2H), 6.84 – 6.76 (m, 2H), 4.79 (d, J = 14.8, 1H), 4.59 – 4.50 (m, 2H), 4.12 – 4.04 (m, 1H), 3.78 (s, 3H), 3.59 (d, J =1 4.8, 1H). ^{13}C NMR (151 MHz, $CDCl_3$) δ = 159.4, 158.0, 150.7, 149.1, 134.5, 133.1, 129.9, 126.8, 124.2, 114.2, 69.3, 56.3, 55.2, 45.5. ESI-MS: calculated $[C_{15}H_{16}N_2O_3+Na]^+$: 307.1053, found: 307.1063.

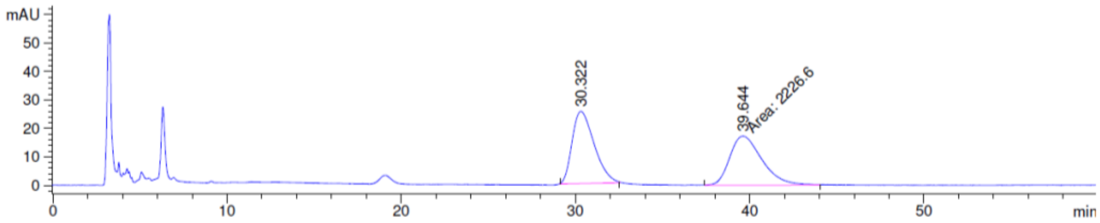

Peak #	RetTime [min]	Type	Width [min]	Area [mAU*s]	Height [mAU]	Area %
1	30.322	BB	1.0346	2242.42847	25.43356	50.1771
2	39.644	MM	2.1496	2226.60083	17.26384	49.8229

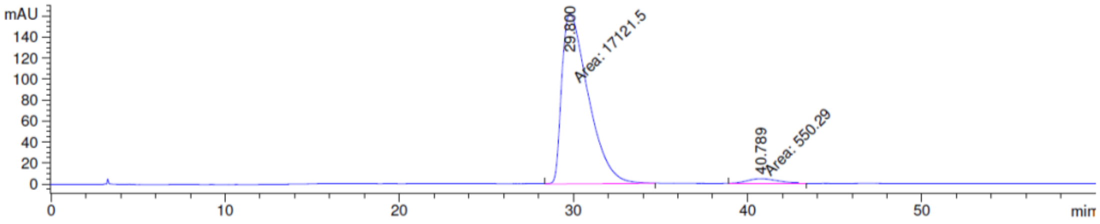

Peak #	RetTime [min]	Type	Width [min]	Area [mAU*s]	Height [mAU]	Area %
1	29.800	MM	1.7603	1.71215e4	162.11203	96.8861
2	40.789	MM	2.0125	550.29022	4.55726	3.1139

3-(4-Methoxybenzyl)-4-methyloxazolidin-2-one (2x)

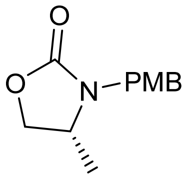

Colorless oil; cyclohexane/THF = 0 mL/2.0 mL, –20 °C, 98% yield, 73% *ee*. $[\alpha]_D^{22}$ = +29.7 (c = 0.57 in $CHCl_3$). HPLC DAICEL CHIRALCEL AS-H, *n*-hexane/2-propanol = 70/30, flow rate = 1 mL/min, λ = 230 nm, retention time: 18.1 min (major), 24.0 min (minor). ^1H NMR (300 MHz, $CDCl_3$) δ = 7.21 (d, J = 8.4, 2H), 6.86 (d, J = 8.6, 2H), 4.71 (d, J = 15.0, 1H), 4.33 (t, J = 8.3, 1H), 4.02 (d, J = 15.1, 1H), 3.85 – 3.76 (m, 4H), 3.72 – 3.55 (m, 1H), 1.20 (d, J = 6.1, 3H). ^{13}C NMR (75 MHz, $CDCl_3$) δ = 159.3, 158.3, 129.5, 127.9, 114.1, 69.0, 55.3, 50.1, 45.1, 17.9. ESI-MS: calculated $[C_{12}H_{15}NO_3+Na]^+$: 244.0944, found: 244.0958.

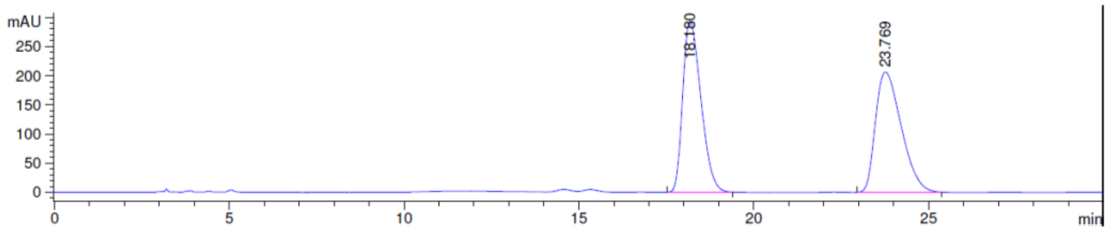

Peak #	RetTime [min]	Type	Width [min]	Area [mAU*s]	Height [mAU]	Area %
1	18.180	BB	0.5669	1.06172e4	292.89273	50.0224
2	23.769	BB	0.8063	1.06077e4	206.17409	49.9776

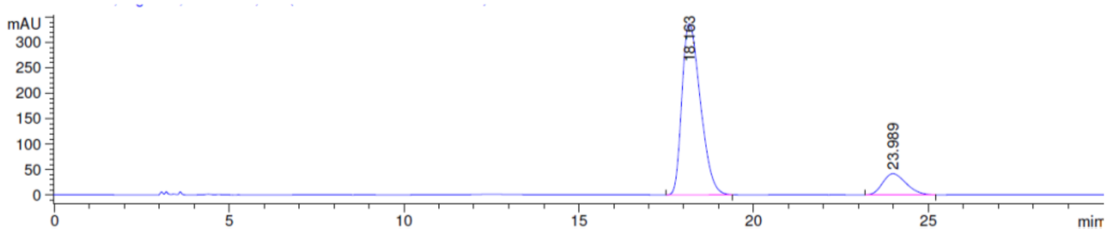

Peak #	RetTime [min]	Type	Width [min]	Area [mAU*s]	Height [mAU]	Area %
1	18.163	BB	0.5750	1.23807e4	336.70538	86.3790
2	23.989	BB	0.7279	1952.29932	41.57281	13.6210

4-Butyl-3-(4-methoxybenzyl)oxazolidin-2-one (2y)

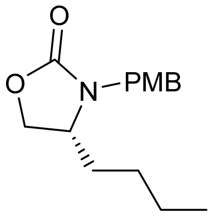

Colorless oil; cyclohexane/THF = 0 mL/2.0 mL, –20 °C, 98% yield, 90% *ee*. $[\alpha]_D^{22}$ = –3.5 (c = 0.71 in $CHCl_3$). HPLC DAICEL CHIRALCEL OD-H, *n*-hexane/2-propanol = 85/15, flow rate = 1 mL/min, λ = 230 nm, retention time: 12.0 min (minor), 14.9 min (major). ^{1}H NMR (300 MHz, $CDCl_3$) δ = 7.20 (dt, *J* = 8.9, 2.6, 2H), 6.86 (dt, *J* = 5.0, 2.8, 2H), 4.72 (dd, *J* = 15.0, 2.6, 1H), 4.28 (ddd, *J* = 11.4, 5.8, 2.9, 1H), 4.06 – 3.88 (m, 2H), 3.79 (t, *J* = 2.8, 3H), 3.63 – 3.46 (m, 1H), 1.67 (tdd, *J* = 10.9, 7.7, 3.6, 1H), 1.53 – 1.35 (m, 1H), 1.36 – 1.06 (m, 4H), 0.94 – 0.80 (m, 3H). ^{13}C NMR (75 MHz, $CDCl_3$) δ = 159.3, 158.5, 129.5, 128.0, 114.1, 67.3, 55.3, 54.0, 45.3, 31.2, 25.9, 22.6, 13.9. ESI-MS: calculated $[C_{15}H_{21}NO_3+Na]^+$: 286.1414, found: 286.1417.

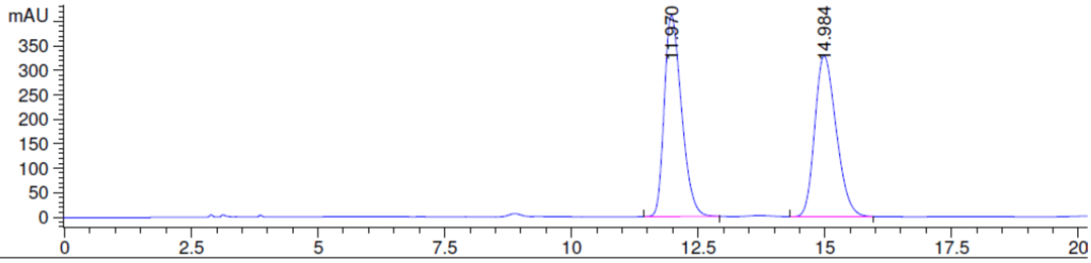

Peak #	RetTime [min]	Type	Width [min]	Area [mAU*s]	Height [mAU]	Area %
1	11.970	BB	0.3650	9783.10254	411.98627	50.2584
2	14.984	VB	0.4563	9682.49219	329.08868	49.7416

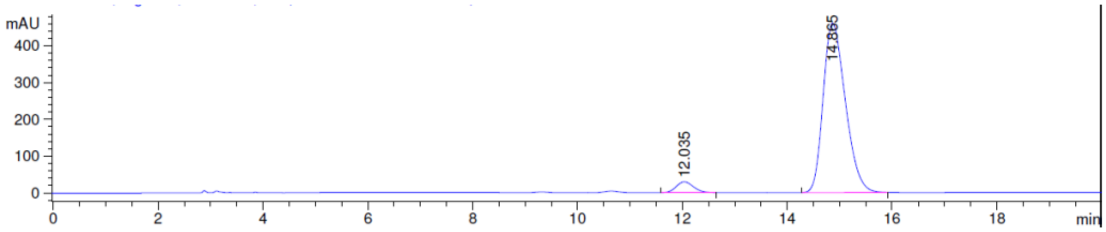

Peak #	RetTime [min]	Type	Width [min]	Area [mAU*s]	Height [mAU]	Area %
1	12.035	BB	0.3588	679.64807	29.50374	4.7661
2	14.865	BB	0.4557	1.35803e4	460.94757	95.2339

4-Isopropyl-3-(4-methoxybenzyl)oxazolidin-2-one (2z)

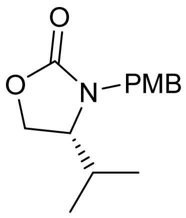

Colorless oil; cyclohexane/THF = 0 mL/2.0 mL, –10 °C, 95% yield, 91% *ee*. $[\alpha]_D^{22}$ = +25.6 (c = 1.17 in $CHCl_3$). HPLC DAICEL CHIRALCEL OD-H, *n*-hexane/2-propanol = 80/20, flow rate = 1 mL/min, λ = 230 nm, retention time: 9.9 min (minor), 14.6 min (major). ^{1}H NMR (400 MHz, $CDCl_3$) δ = 7.20 (t, J = 5.7, 2H), 6.89 – 6.83 (m, 2H), 4.81 (d, J = 15.0, 1H), 4.14 (t, J = 9.0, 1H), 4.05 (dd, J = 8.9, 6.0, 1H), 3.91 (d, J = 15.0, 1H), 3.80 (s, 3H), 3.52 (ddd, J = 9.3, 5.9, 3.6, 1H), 2.06 (dtd, J = 13.9, 6.9, 3.7, 1H), 0.84 (dd, J = 14.2, 6.9, 6H). ^{13}C NMR (101 MHz, $CDCl_3$) δ = 159.3, 158.7, 129.5, 127.9, 114.1, 62.7, 58.1, 55.3, 45.4, 27.1, 17.7, 14.2. ESI-MS: calculated $[C_{14}H_{19}NO_3+Na]^+$: 272.1257, found: 272.1258.

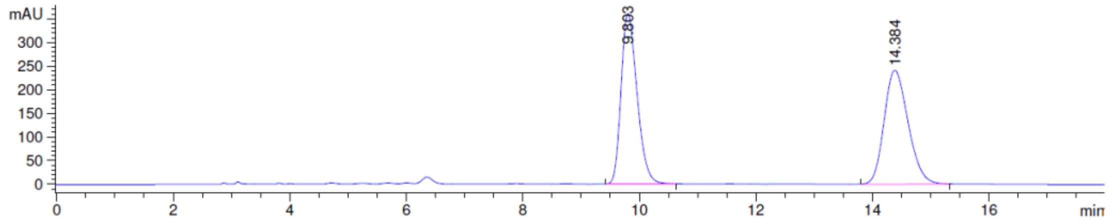

Peak #	RetTime [min]	Type	Width [min]	Area [mAU*s]	Height [mAU]	Area %
1	9.803	BB	0.2947	6882.76318	360.57819	49.9823
2	14.384	BB	0.4468	6887.64307	240.73291	50.0177

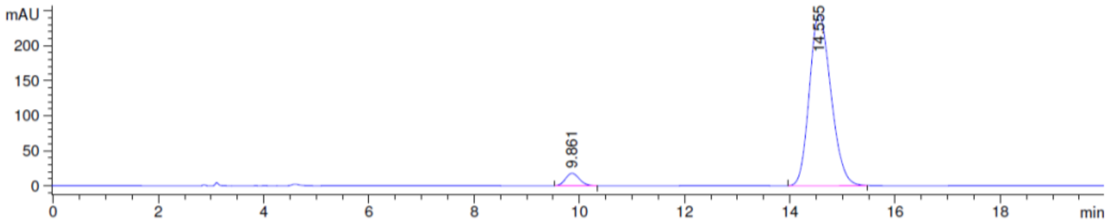

Peak #	RetTime [min]	Type	Width [min]	Area [mAU*s]	Height [mAU]	Area %
1	9.861	BB	0.2879	334.97507	18.01498	4.5815
2	14.555	BB	0.4437	6976.42334	244.67567	95.4185

4-Cyclopropyl-3-(4-methoxybenzyl)oxazolidin-2-one (2aa)

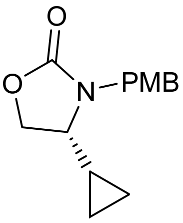

Colorless oil; cyclohexane/THF = 0 mL/2.0 mL, –10 °C, 92% yield, 94% *ee*. $[\alpha]_D^{22}$ = +57.8 (c = 1.28 in $CHCl_3$). HPLC DAICEL CHIRALCEL OD-H, *n*-hexane/2-propanol = 90/10, flow rate = 1 mL/min, λ = 230 nm, retention time: 23.9 min (minor), 26.8 min (major). ^{1}H NMR (300 MHz, $CDCl_3$) δ = 7.24 – 7.15 (m, 2H), 6.88 – 6.80 (m, 2H), 4.78 (d, J = 15.1, 1H), 4.34 (t, J = 8.7, 1H), 4.23 (d, J = 15.1, 1H), 4.08 (dd, J = 8.7, 7.1, 1H), 3.80 (d, J = 4.6, 3H), 2.79 (td, J = 8.9, 7.1, 1H), 0.86 – 0.74 (m, 1H), 0.72 – 0.59 (m, 1H), 0.48 (ddd, J = 17.5, 8.7, 5.3, 1H), 0.17 (td, J = 10.0, 4.8, 1H), −0.01 (td, J = 10.1, 4.9, 1H). ^{13}C NMR (75 MHz, $CDCl_3$) δ = 159.2, 158.6, 129.4, 128.5, 114.1, 67.8, 59.9, 55.4, 45.5, 13.2, 4.6, 0.0. ESI-MS: calculated $[C_{14}H_{17}NO_3+Na]^+$: 270.1101, found: 270.1101.

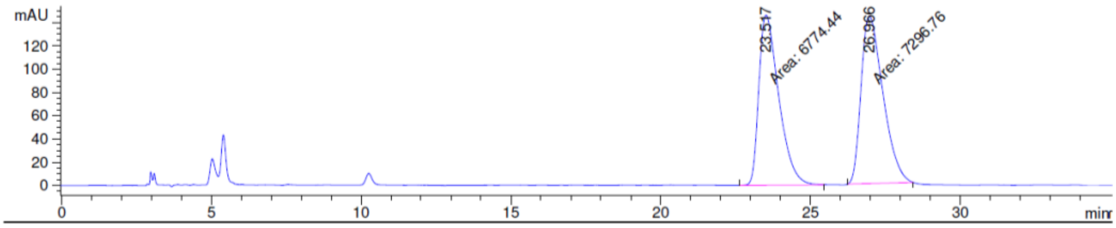

Peak #	RetTime [min]	Type	Width [min]	Area [mAU*s]	Height [mAU]	Area %
1	23.517	MM	0.7720	6774.43896	146.24544	48.1440
2	26.966	MM	0.8458	7296.75537	143.78401	51.8560

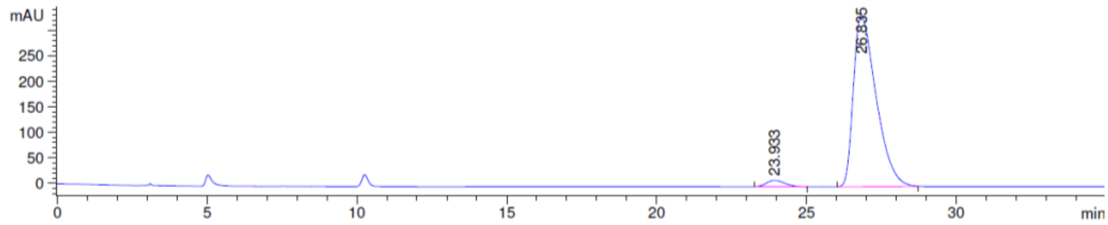

Peak #	RetTime [min]	Type	Width [min]	Area [mAU*s]	Height [mAU]	Area %
1	23.933	BB	0.5505	553.58569	12.87710	3.0332
2	26.835	BB	0.7866	1.76973e4	336.46545	96.9668

4-Cyclohexyl-3-(4-methoxybenzyl)oxazolidin-2-one (2ab)

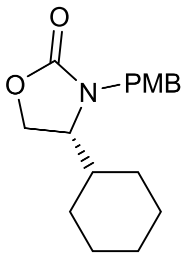

Colorless oil; cyclohexane/THF = 2.0 mL/0 mL, 0 °C, 94% yield, 94% *ee*. $[\alpha]_D^{22}$ = –21.1 (c = 0.78 in $CHCl_3$). HPLC DAICEL CHIRALCEL OD-H, *n*-hexane/2-propanol = 80/20, flow rate = 1 mL/min, λ = 230 nm, retention time: 9.7 min (minor), 14.9 min (major). ^1H NMR (300 MHz, $CDCl_3$) δ = 7.25 – 7.16 (m, 2H), 6.91 – 6.80 (m, 2H), 4.79 (d, J = 15.0, 1H), 4.18 – 4.06 (m, 2H), 3.92 (d, J = 15.0, 1H), 3.80 (s, 3H), 3.53 – 3.42 (m, 1H), 1.87 – 1.61 (m, 4H), 1.54 (d, J = 12.0, 1H), 1.41 (d, J = 12.9, 1H), 1.32 – 0.97 (m, 4H), 0.95 – 0.75 (m, 1H). ^{13}C NMR (75 MHz, $CDCl_3$) δ = 159.3, 158.7, 129.5, 128.0, 114.1, 63.6, 57.8, 55.3, 45.5, 37.6, 28.3, 26.3, 26.2, 25.6, 24.8. ESI-MS: calculated $[C_{17}H_{23}NO_3+Na]^+$: 312.1570, found: 312.1575.

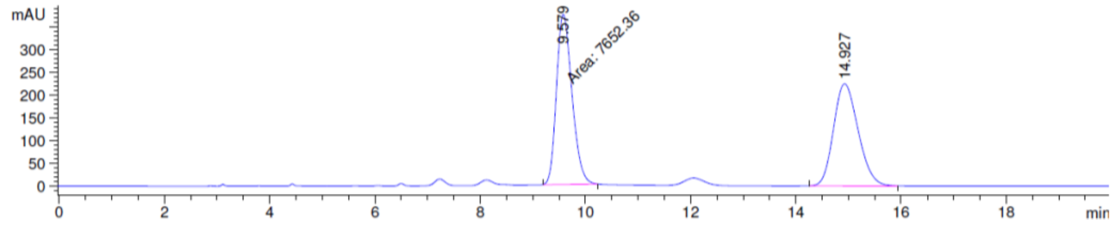

Peak #	RetTime [min]	Type	Width [min]	Area [mAU*s]	Height [mAU]	Area %
1	9.579	MM	0.3414	7652.36328	373.60800	51.1419
2	14.927	BB	0.5060	7310.65088	224.45422	48.8581

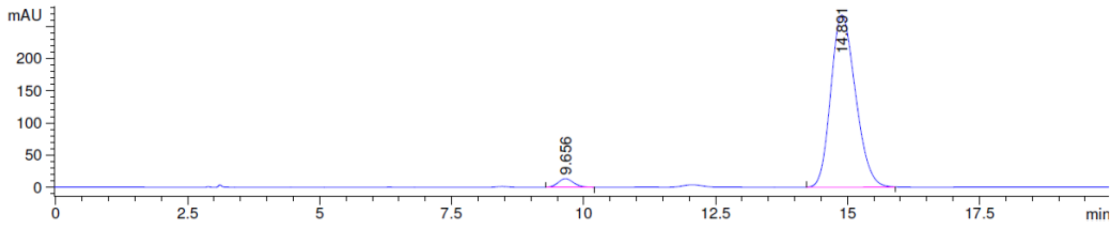

Peak #	RetTime [min]	Type	Width [min]	Area [mAU*s]	Height [mAU]	Area %
1	9.656	BB	0.3169	278.59885	13.49511	3.0991
2	14.891	BB	0.5046	8710.98438	267.72891	96.9009

4-(*tert*-Butyl)-3-(4-methoxybenzyl)oxazolidin-2-one (2ac)

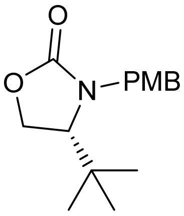

Colorless oil; cyclohexane/THF = 2.0 mL/0 mL, 0 °C, 83% yield, 90% *ee*. $[\alpha]_D^{22}$ = +53.6 (c = 0.89 in $CHCl_3$). HPLC DAICEL CHIRALCEL AS-H, *n*-hexane/2-propanol = 70/30, flow rate = 1 mL/min, λ = 230 nm, retention time: 15.5 min (major), 21.3 min (minor). ^{1}H NMR (300 MHz, $CDCl_3$) δ = 7.17 (d, J = 8.5, 2H), 6.87 (d, J = 8.7, 2H), 5.00 (d, J = 15.3, 1H), 4.20 – 4.07 (m, 3H), 3.80 (s, 3H), 3.26 (dd, J = 8.5, 4.9, 1H), 0.94 (s, 9H). ^{13}C NMR (75 MHz, $CDCl_3$) δ = 160.1, 159.2, 129.3, 128.3, 114.2, 65.0, 62.2, 55.3, 48.6, 35.1, 25.9. ESI-MS: calculated $[C_{15}H_{21}NO_3+Na]^+$: 286.1414, found: 286.1416.

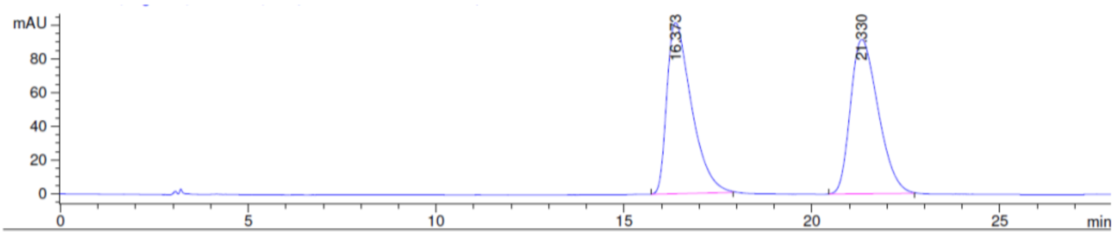

Peak #	RetTime [min]	Type	Width [min]	Area [mAU*s]	Height [mAU]	Area %
1	16.373	BB	0.6788	4629.54199	101.56145	49.5750
2	21.330	BB	0.7813	4708.91602	91.66784	50.4250

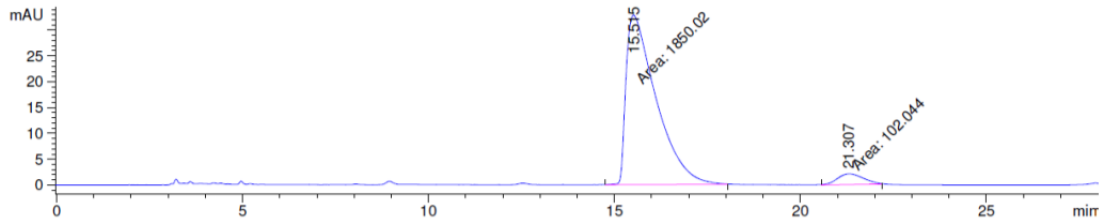

Peak #	RetTime [min]	Type	Width [min]	Area [mAU*s]	Height [mAU]	Area %
1	15.515	MM	0.9394	1850.01624	32.82386	94.7725
2	21.307	MM	0.8082	102.04350	2.10424	5.2275

Hexahydro-3*H*-oxazolo[3,4-*a*]pyridin-3-one (2ad)

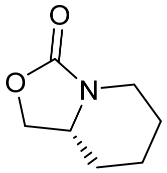

Colorless oil; cyclohexane/THF = 0 mL/2.0 mL, –20 °C, 99% yield, 70% *ee*. $[\alpha]_D^{22}$ = –15.8 (c = 1.18 in $CHCl_3$). HPLC DAICEL CHIRALCEL AS-H, *n*-hexane/2-propanol = 70/30, flow rate = 1 mL/min, λ = 210 nm, retention time: 13.6 min (minor), 15.1 min (major). ^{1}H NMR (300 MHz, $CDCl_3$) δ = 4.39 (t, J = 8.3, 1H), 3.86 (ddd, J = 6.3, 5.6, 3.0, 2H), 3.64 (tdd, J = 10.9, 7.2, 3.5, 1H), 2.82 (ddd, J = 10.2, 9.3, 3.5, 1H), 1.88 (ddt, J = 15.4, 7.7, 3.6, 2H), 1.73 – 1.57 (m, 1H), 1.53 – 1.18 (m, 3H). ^{13}C NMR (75 MHz, $CDCl_3$) δ = 157.1, 68.2, 54.5, 41.5, 30.6, 24.3, 22.7. ESI-MS: calculated $[C_7H_{11}NO_2+Na]^+$: 164.0682, found: 164.0688.

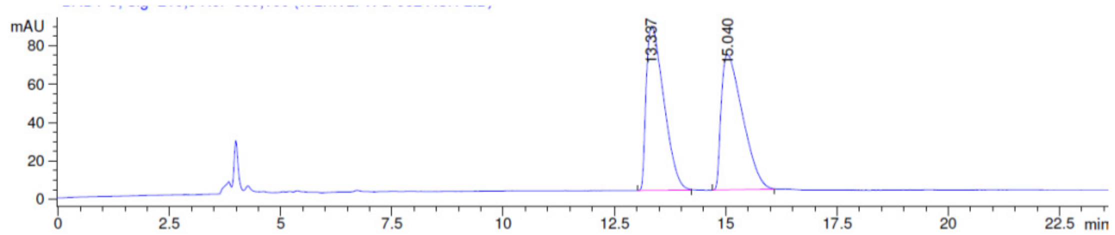

Peak #	RetTime [min]	Type	Width [min]	Area [mAU*s]	Height [mAU]	Area %
1	13.337	BB	0.4130	2350.56812	85.44250	50.0414
2	15.040	BB	0.4902	2346.67505	70.52921	49.9586

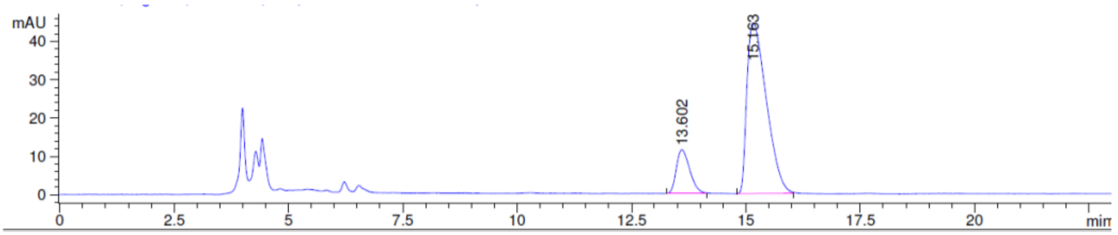

Peak #	RetTime [min]	Type	Width [min]	Area [mAU*s]	Height [mAU]	Area %
1	13.602	BB	0.3082	232.64534	11.39627	15.0576
2	15.163	BB	0.4368	1312.39136	44.53253	84.9424

(D) Scaled-up reaction and transformations of the products

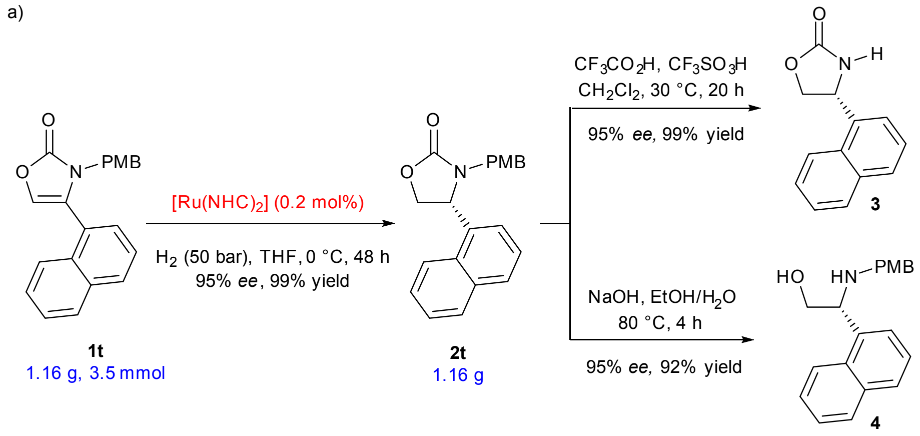

In a glove box, to a flame-dried screw-capped tube equipped with a magnetic stir bar was added [Ru(2-methylallyl)$_2$(COD)] (0.10 mmol; COD = cyclooctadiene), (*R,R*)-SINpEt·HBF_4 (0.20 mmol), and dry NaO*t*Bu (0.24 mmol). The mixture was suspended in *n*-hexane (5.0 mL) and stirred at 70 °C for 16 h to form the catalyst mixture (0.02 M). To a 20 mL glass vial, **1t** (1.160 g, 3.50 mmol), THF (7.0 mL, 0.5 M), and 0.35 mL (0.007 mmol, 0.2 mol%) of the catalyst mixture was added under argon. The glass vial was placed in a 150 mL stainless steel autoclave under an argon atmosphere. The autoclave was pressurized and depressurized with hydrogen gas five times before 50 bar was set. The hydrogenation was performed under 50 bar H_2 for 48 h at 0 °C. After the autoclave was carefully depressurized, the mixture was concentrated and purified by flash column chromatography on silica gel (*n*-pentane/EtOAc = 10/1, 2.5/1) to afford the pure product **2t** (1.159 g, 95% *ee*, 99% yield).

The deprotection of PMB group was conducted according to a modified literature procedure.[4] Under argon, TFA (15.0 mmol, 5.0 equiv.) and TfOH (9.0 mmol,

3.0 equiv.) were added to a solution of enantiomerically enriched **2t** (1.0 g, 3.0 mmol, 1.0 equiv.) in CH_2Cl_2 (30 mL, 0.1 M). The mixture was stirred at 30 °C for 20 h (monitored by TLC), then quenched by the addition of saturated aqueous $NaHCO_3$ solution and extracted with CH_2Cl_2 (3 x 50 mL). The combined organic layers were dried over Na_2SO_4, evaporated *in vacuo*, and further purified by column chromatography on silica gel (*n*-pentane/EtOAc = 4/1, later 1.5/1) to give pure compound **3** (95% *ee*, 99% yield).

4-(Naphthalen-1-yl)oxazolidin-2-one (3)

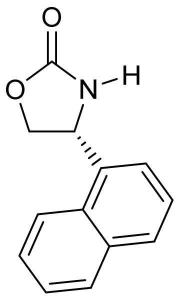

Colorless solid; 99% yield, 95% *ee* $[\alpha]_D^{22}$ = –176.7 (c = 0.20 in $CHCl_3$), HPLC DAICEL CHIRALCEL AD-H, *n*-hexane/2-propanol = 90/10, flow rate = 1.0 mL/min, λ = 254 nm, retention time: 13.0 min (major), 15.0 min (minor). ^{1}H NMR (300 MHz, $CDCl_3$) δ = 7.99 – 7.90 (m, 1H), 7.86 (d, *J* = 8.2, 1H), 7.81 – 7.74 (m, 1H), 7.66 (d, *J* = 7.1, 1H), 7.61 – 7.48 (m, 3H), 5.98 (s, 1H), 5.81 – 5.69 (m, 1H), 5.02 (t, *J* = 8.7, 1H), 4.23 (dd, *J* = 8.5, 6.5, 1H). ^{13}C NMR (101 MHz, $CDCl_3$) δ = 159.7, 135.0, 134.0, 123.0, 129.4, 129.1, 127.0, 126.2, 125.6, 122.2, 121.7, 71.9, 53.1. ESI-MS: calculated $[C_{13}H_{11}NO_2+Na]^+$: 236.0682, found: 236.0683.

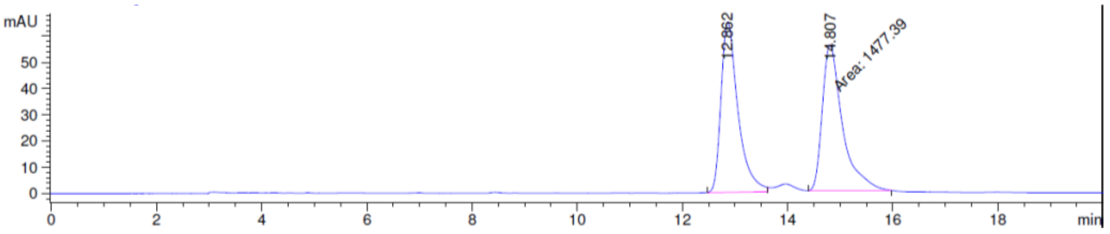

Peak #	RetTime [min]	Type	Width [min]	Area [mAU*s]	Height [mAU]	Area %
1	12.862	BB	0.3358	1457.87927	64.96033	49.6676
2	14.807	MM	0.4434	1477.39429	55.53014	50.3324

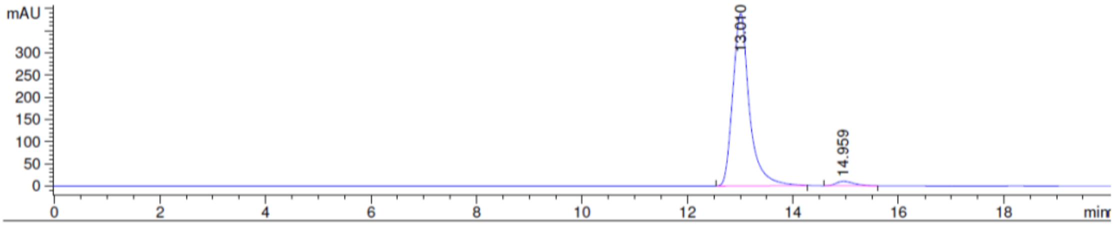

Peak #	RetTime [min]	Type	Width [min]	Area [mAU*s]	Height [mAU]	Area %
1	13.010	BB	0.3293	8450.86035	387.64233	97.4089
2	14.959	BB	0.3564	224.79488	9.59180	2.5911

Hydrolysis: to a flask oxazolidinone **2t** (67 mg, 0.2 mmol), EtOH (2.5 mL) and 10% NaOH aqueous solution (1.0 mL) was added. The reaction mixture was stirred for 4 h at 80 °C (monitored by TLC). The reaction mixture was then extracted with EtOAc three times. The combined organic solution was dried over anhydrous Na_2SO_4, filtered and concentrated in vacuo. The residue was further purified by column chromatography on silica gel (*n*-pentane/EtOAc = 3/1, later 1/2) to give pure compound **4** (95% *ee*, 92% yield).

2-((4-methoxybenzyl)amino)-2-(naphthalen-1-yl)ethan-1-ol (4)

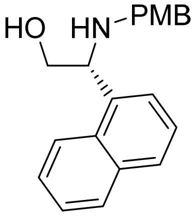

Colorless solid; 92% yield, 95% *ee* $[\alpha]_D^{22}$ = –31.3 (c = 1.44 in $CHCl_3$), HPLC DAICEL CHIRALCEL AD-H, *n*-hexane/2-propanol = 80/20, flow rate = 0.8 mL/min, λ = 230 nm, retention time: 8.9 min (minor), 9.6 min (major). ^{1}H NMR (300 MHz, $CDCl_3$) δ = 8.19 – 8.03 (m, 1H), 7.99 – 7.86 (m, 1H), 7.82 (d, *J*=8.1, 1H), 7.65 (d, *J*=6.6, 1H), 7.58 – 7.45 (m, 3H), 7.22 (d, *J*=8.6, 2H), 6.94 – 6.78 (m, 2H), 4.73 (dd, *J*=8.4, 4.0, 1H), 3.90 (dd, *J*=10.9, 4.1, 1H), 3.84 – 3.72 (m, 4H), 3.69 – 3.54 (m, 2H), 2.43 (s, 2H). ^{13}C NMR (75 MHz, $CDCl_3$) δ = 158.76, 135.93, 134.03, 132.22, 131.81, 129.51, 129.08, 127.99, 126.22, 125.67, 125.57, 123.50, 122.67, 113.87, 66.21, 58.95, 55.31, 50.71. ESI-MS: calculated $[C_{20}H_{21}NO_2+H^+]^+$: 308.1645, found: 308.1656.

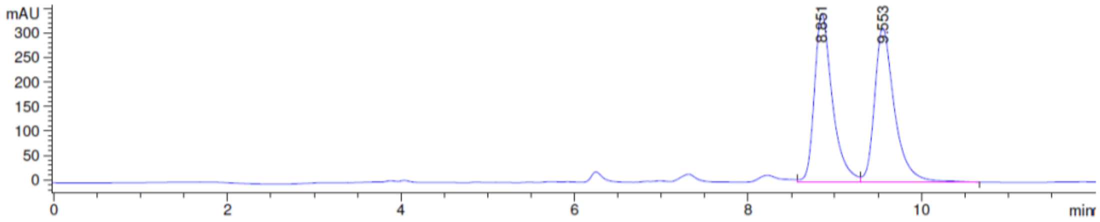

Peak #	RetTime [min]	Type	Width [min]	Area [mAU*s]	Height [mAU]	Area %
1	8.851	VV	0.2153	4969.12842	344.43909	49.5625
2	9.553	VB	0.2395	5056.84912	314.60724	50.4375

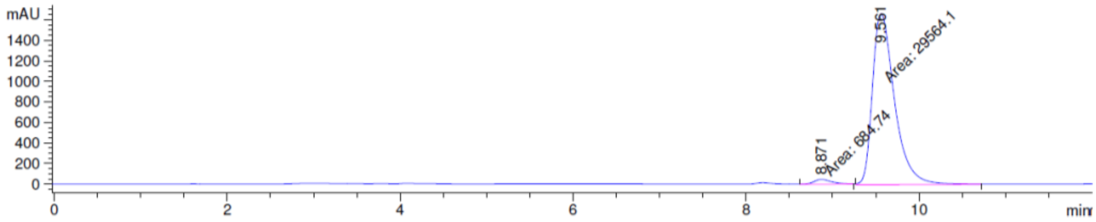

Peak #	RetTime [min]	Type	Width [min]	Area [mAU*s]	Height [mAU]	Area %
1	8.871	MM	0.2415	684.73975	47.24724	2.2637
2	9.561	MM	0.2962	2.95641e4	1663.80090	97.7363

b)

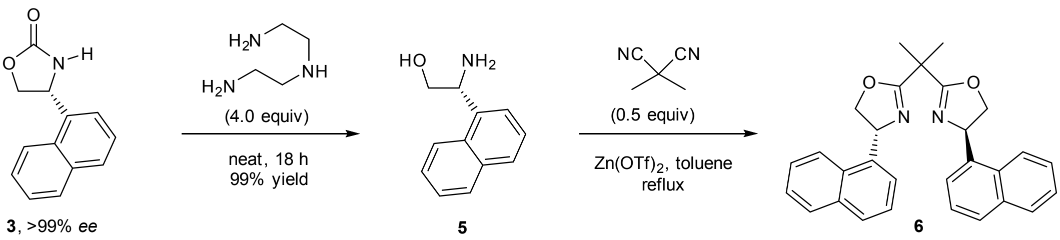

The cleavage of the 2-oxazolidinone **3** was conducted according to a modified literature procedure.[5] Under argon, **3** (1.5 mmol, 1.0 equiv.) and diethylenetriamine (6.0 mmol, 4.0 equiv.) were added to a dry vial. The mixture was stirred for 18 h at 140 °C and directly purified by column chromatography on silica gel ($CH_2Cl_2/CH_3OH/Et_3N$ = 10/1/0.1) to give pure amino alcohol **5** (99% yield).

(*R*)-2-Amino-2-(naphthalen-1-yl)ethan-1-ol (5)

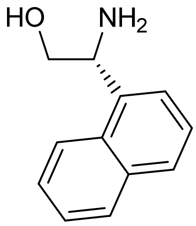

Colorless solid; $[\alpha]_D^{22}$ = –73.2 (c = 0.51 in MeOH). ^{1}H NMR (300 MHz, CD_3OD) δ = 8.13 (d, J = 8.4, 1H), 7.87 (d, J = 7.9, 1H), 7.79 (d, J = 8.2, 1H), 7.61 (d, J = 7.1, 1H), 7.57 – 7.39 (m, 3H), 4.94 – 4.85 (m, 1H), 3.89 (dd, J = 11.1, 3.8, 1H), 3.59 (dd, J = 11.0, 8.1, 1H). ^{13}C NMR (75 MHz, CD_3OD) δ = 139.0, 136.2, 133.2, 130.9, 129.8, 128.2, 127.5, 127.3, 125.1, 124.5, 69.1, 54.6. ESI-MS: calculated $[C_{12}H_{11}O]^+$ $[M – NH_2]^+$: 171.0804, found: 171.0806.

According to a modified literature procedure:[6] A flame-dried screw-capped tube was charged with 2,2-dimethylmalononitrile (0.6 mmol, 1.0 equiv.) and zinc triflate (0.6 mmol, 1.0 equiv.). The system was purged with argon and anhydrous toluene (6 mL, 0.1 M) was added. The solution was stirred for 5 min and the β-amino alcohol **4** (1.2 mmol, 2.0 equiv.) was added. The solution was heated at reflux (150 °C) for 60 h. The system was allowed to cool to room temperature and the mixture was diluted with 50 mL of EtOAc and brine (100 mL), extracted with EtOAc (5 × 75 mL), dried with $MgSO_4$ and purified by column chromatography on silica gel (EtOAc/*n*-pentane = 3/1, later 1.5/1) to give pure **5** (74% yield)

(*4R,4'R*)-2,2'-(Propane-2,2-diyl)bis(4-(naphthalen-1-yl)-4,5-dihydrooxazole) (6)

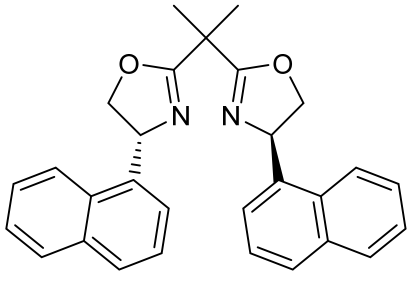

Colorless solid; $[\alpha]_D^{22}$ = –213.2 (c = 0.84 in $CHCl_3$). ^{1}H NMR (300 MHz, $CDCl_3$) δ = 7.94 – 7.87 (m, 2H), 7.86 – 7.75 (m, 4H), 7.61 (d, J = 7.0 Hz, 2H), 7.57 – 7.42 (m, 6H), 6.01 (dd, J = 10.2, 8.1 Hz, 2H), 4.98 (dd, J = 10.3, 8.2 Hz, 2H), 4.15 (t, J = 8.1 Hz, 2H), 1.83 (s, 6H). ^{13}C NMR (101 MHz, $CDCl_3$) δ = 170.6, 138.4, 133.9, 130.6, 129.1, 127.9, 126.3, 125.8, 125.6, 123.5, 122.8, 75.1, 66.3, 39.4, 24.7. ESI-MS: calculated $[C_{29}H_{26}N_2O_2+H]^+$: 435.2067, found: 435.2061.

c)

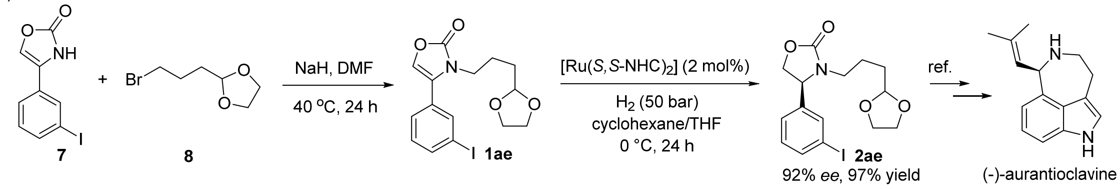

According to procedure A (Page S3), 4-(3-iodophenyl)oxazol-2(3H)-one **7** was synthesized. Then, sodium hydride (60% purity, 1.2 equiv.) was added portionwise to a solution of the oxazolone **7** (1.0 equiv.) in DMF (0.5 M) at 0 °C. The mixture was stirred at 0 °C for 45 min, before 2-(3-bromopropyl)-1,3-dioxolane **8** (1.2 equiv.) was added.[7] The mixture was then stirred at 40 °C and after full consumption of the starting material, as indicated by TLC analysis (24 h), the reaction was quenched with water. EtOAc was added and the organic layers were washed twice with 5wt% aqueous LiCl solution to remove DMF, followed by additional washing with brine. After drying over $MgSO_4$, the crude product was purified by column chromatography (*n*-pentane/EtOAc = 4/1, later 2/1) to give pure **1ae**.

3-(3-(1,3-Dioxolan-2-yl)propyl)-4-(3-iodophenyl)oxazol-2(3H)-one (1ae)

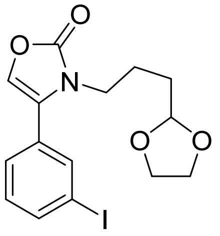

^{1}H NMR (400 MHz, $CDCl_3$) δ = 7.82 – 7.73 (m, 1H), 7.71 (t, J = 1.7 Hz, 1H), 7.36 – 7.28 (m, 1H), 7.19 (t, J = 7.8 Hz, 1H), 6.80 (s, 1H), 4.76 (t, J = 4.2 Hz, 1H), 3.87 – 3.83 (m, 2H), 3.78 (dt, J = 7.5, 4.5 Hz, 2H), 3.70 – 3.62 (m, 2H), 1.69 – 1.52 (m, 4H). ^{13}C NMR (101 MHz, $CDCl_3$) δ = 156.0, 138.6, 137.2, 130.7, 128.8, 128.1, 127.6, 124.6, 103.7, 94.7, 65.0, 42.3, 30.4, 23.0. ESI-MS: calculated $[C_{15}H_{16}INO_4+Na]^+$: 424.0016, found: 424.0019.

In a glove box, to a flame-dried screw-capped tube equipped with a magnetic stir bar was added [Ru(2-methylallyl)$_2$(COD)] (0.10 mmol; COD = cyclooctadiene), (*S,S*)-SINpEt·HBF_4 (0.20 mmol), and dry NaO*t*-Bu (0.24 mmol). The mixture was suspended in *n*-hexane (5.0 mL) and stirred at 70 °C for 16 h to form the catalyst mixture (0.02 M). To a glass vial, substrates **1ae** (0.20 mmol), cyclohexane/THF = 1.0 mL/1.0 mL, and 0.2 mL of the catalyst mixture was added under argon. The glass vial

was placed in a 150 mL stainless steel autoclave under an argon atmosphere. The autoclave was pressurized and depressurized with hydrogen gas five times before 50 bar was set. The hydrogenation was performed under 50 bar H_2 at 0 °C for 24 h. After the autoclave was carefully depressurized, the mixture was directly purified by flash column chromatography on silica gel (*n*-pentane/EtOAc = 3/1, later 1/1) to afford the desired product **2ae** with 92% *ee* and in 97% yield.[8]

(*S*)-3-(3-(1,3-Dioxolan-2-yl)propyl)-4-(3-iodophenyl)oxazolidin-2-one (2ae)

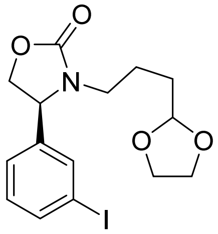

Colorless oil; cyclohexane/THF = 1.0 mL/1.0 mL, 0 °C, 97% yield, 92% *ee*. $[\alpha]_D^{20}$ = +49.6 (c = 1.0 in $CHCl_3$). HPLC DAICEL CHIRALCEL AD-H, *n*-hexane/2-propanol = 80/20, flow rate = 1 mL/min, λ = 230 nm, retention time: 11.36 min (minor), 13.19 min (major). ^{1}H NMR (300 MHz, $CDCl_3$) δ = 7.70 (ddd, *J* = 7.8, 1.6, 1.2 Hz, 1H), 7.62 (t, *J* = 1.7 Hz, 1H), 7.30 – 7.21 (m, 1H), 7.14 (t, *J* = 7.7 Hz, 1H), 4.82 (t, *J* = 4.0 Hz, 1H), 4.72 (dd, *J* = 8.9, 6.2 Hz, 1H), 4.59 (t, *J* = 8.8 Hz, 1H), 4.06 (dd, *J* = 8.7, 6.2 Hz, 1H), 3.96 – 3.76 (m, 4H), 3.60 – 3.37 (m, 1H), 2.88 – 2.68 (m, 1H), 1.68 – 1.50 (m, 4H). ^{13}C NMR (75 MHz, $CDCl_3$) δ = 158.1, 140.4, 138.2, 136.0, 131.1, 126.1, 103.7, 95.0, 69.6, 64.96, 64.94, 58.8, 41.9, 30.6, 21.1. ESI-MS: calculated $[C_{15}H_{18}INO_4+Na]^+$: 426.0173, found: 426.0178.

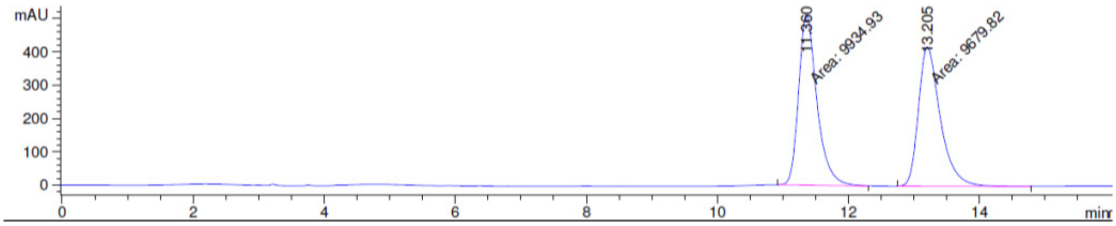

Peak #	RetTime [min]	Type	Width [min]	Area [mAU*s]	Height [mAU]	Area %
1	11.360	MM	0.3238	9934.93066	511.30988	50.6503
2	13.205	MM	0.3868	9679.81641	417.11905	49.3497
Totals :				1.96147e4	928.42892	

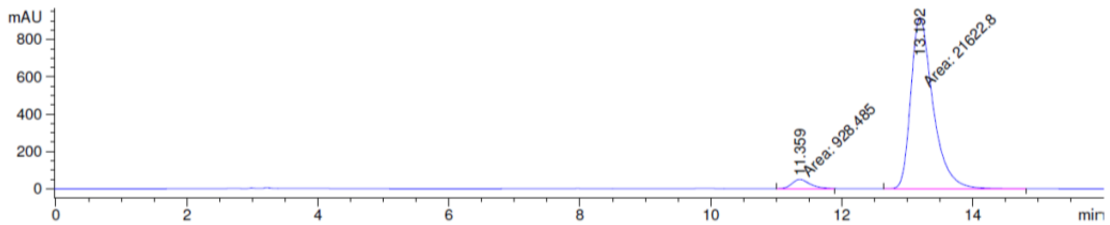

Peak #	RetTime [min]	Type	Width [min]	Area [mAU*s]	Height [mAU]	Area %
1	11.359	MM	0.3140	928.48523	49.28015	4.1172
2	13.192	MM	0.3921	2.16228e4	918.98822	95.8828
Totals :				2.25513e4	968.26837	

(E) References

(1) Urban, S.; Ortega, N.; Glorius, F. *Angew. Chem., Int. Ed.* **2011**, *50*, 3803.

(2) Wang, Q.; Tan, X.; Zhu, Z.; Dong, X.-Q.; Zhang, X. *Tetrahedron Lett.* **2016**, *57*, 658.

(3) Saxena, A.; Perez, F.; Krische, M. J. *J. Am. Chem. Soc.* **2015**, *137*, 5883.

(4) Li, W.; Schlepphorst, C.; Daniliuc, C.; Glorius, F. *Angew. Chem., Int. Ed.* **2016**, *55*, 3300.

(5) Noshita, M.; Shimizu, Y.; Morimoto, H.; Ohshima, T. *Org. Lett.* **2016**, *18*, 6062.

(6) Cornejo, A.; Fraile, J. M.; García, J. I.; Gil, M. J.; Martínez-Merino, V.; Mayoral, J. A.; Pires, E.; Villalbaa, I. *Synlett*, **2005**, 2321.

(7) Varseev, G. N.; Maier, M. E. *Org. Lett.* **2005**, *7*, 3881.

(8) Park, J.; Kim, D.-H.; Das, T.; Cho, C.-G. *Org. Lett.* **2016**, *18*, 5098.

(F) Copies of NMR spectra

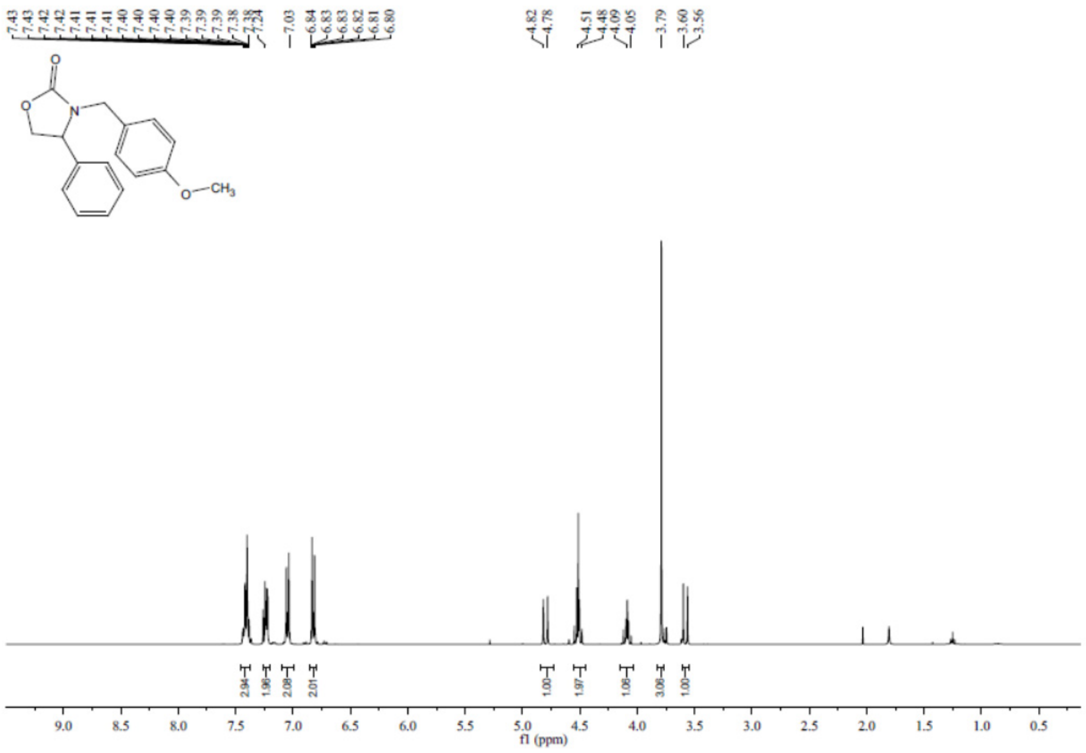

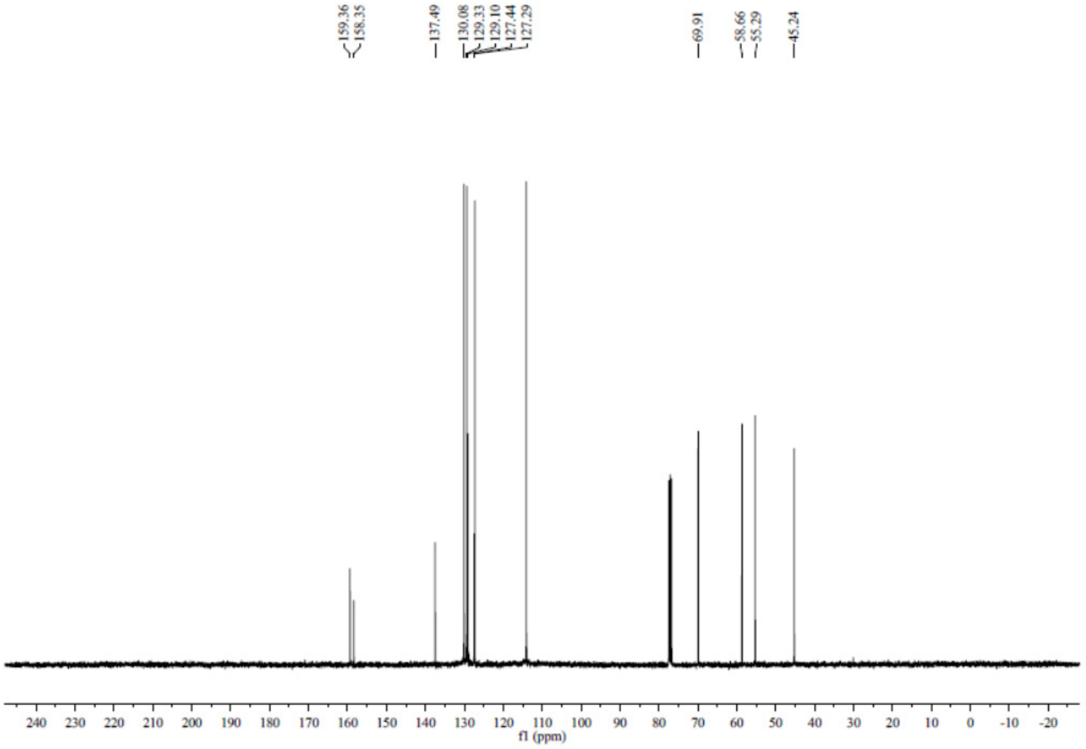

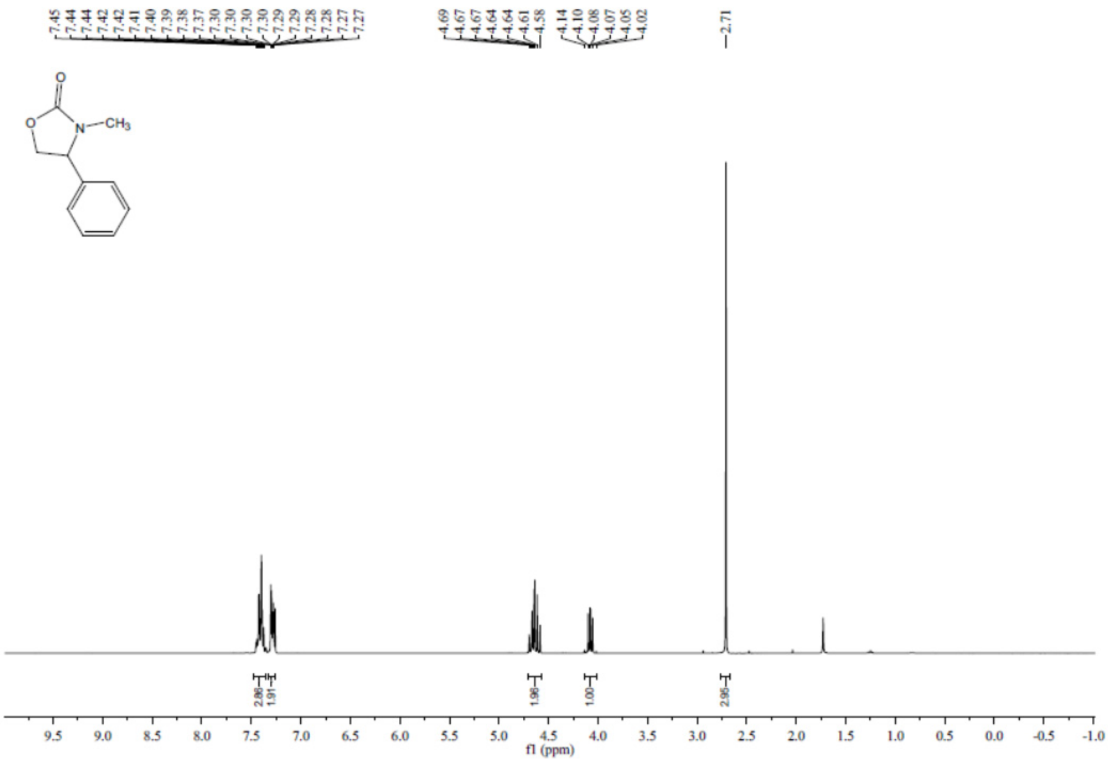
7.45 7.44 7.44 7.42 7.42 7.41 7.40 7.39 7.38 7.37 7.30 7.30 7.30 7.30 7.30 7.29 7.29 7.28 7.28 7.27 7.27
4.69 4.67 4.67 4.64 4.64 4.61 4.58
4.14 4.10 4.08 4.07 4.05 4.02
2.71
O
N
CH3
2.86
1.91
1.98
1.00
2.95
9.5 9.0 8.5 8.0 7.5 7.0 6.5 6.0 5.5 5.0 4.5 4.0 3.5 3.0 2.5 2.0 1.5 1.0 0.5 0.0 -0.5 -1.0
f1 (ppm)

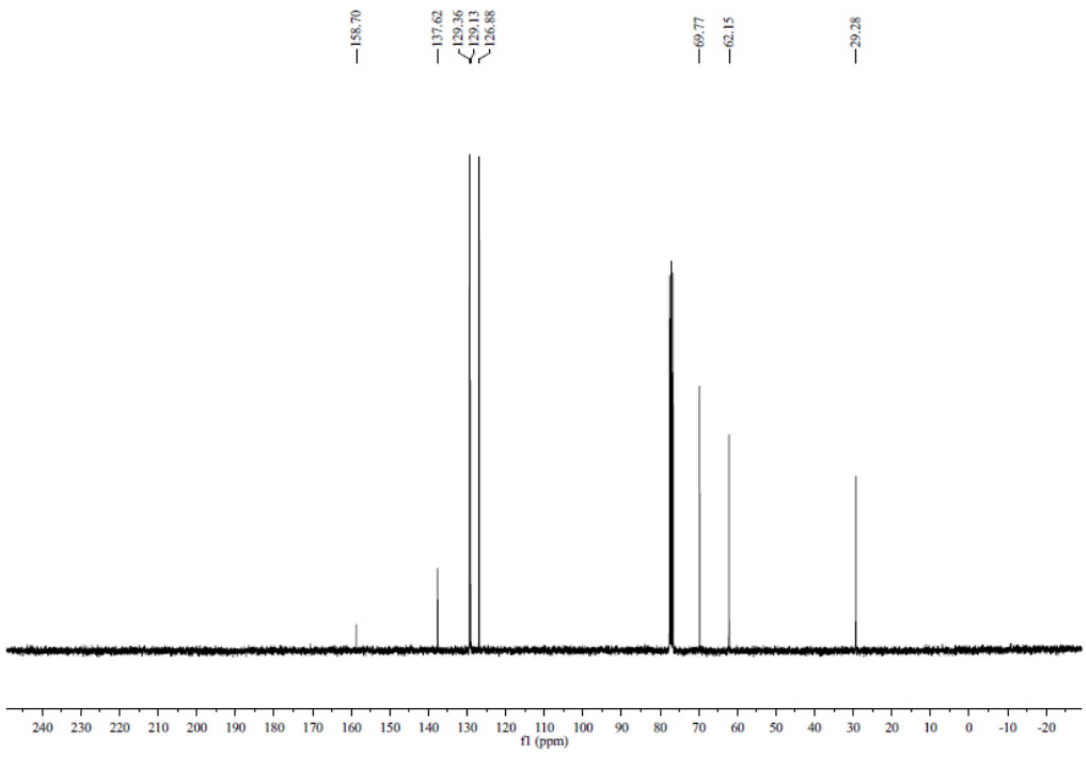
158.70
137.62
129.36
129.13
126.88
69.77
62.15
29.28
240 230 220 210 200 190 180 170 160 150 140 130 120 110 100 90 80 70 60 50 40 30 20 10 0 -10 -20
f1 (ppm)

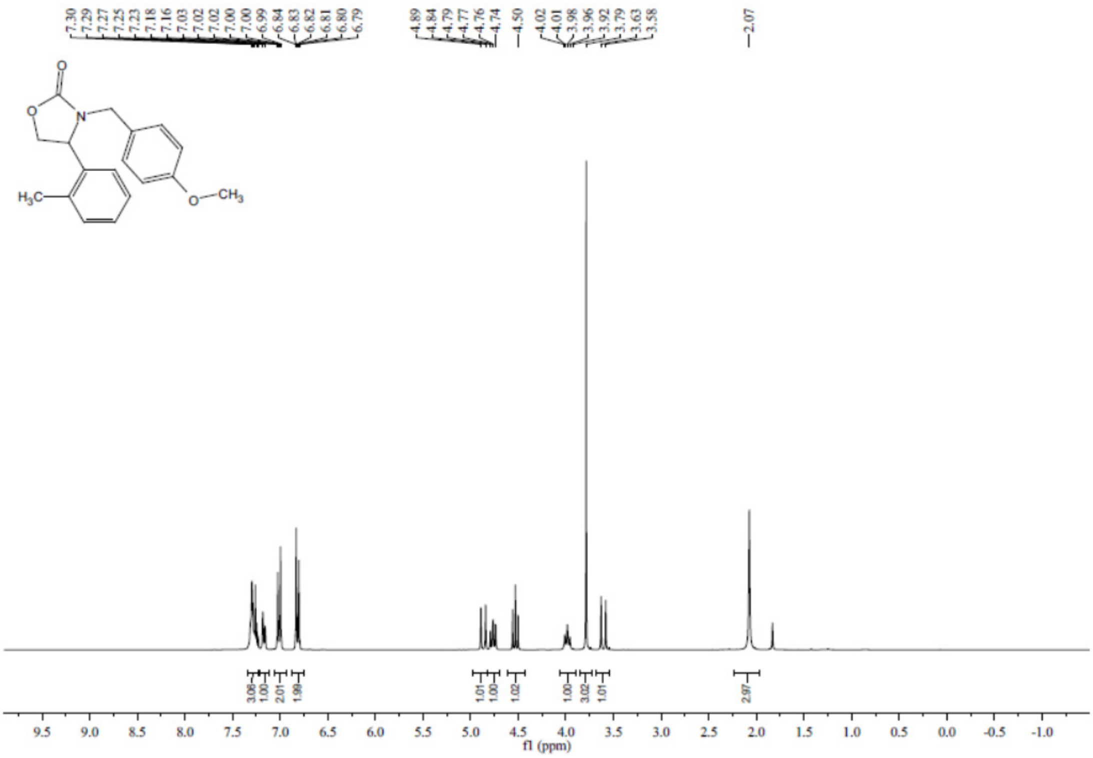
7.30 7.29 7.27 7.25 7.23 7.18 7.16 7.03 7.02 7.02 7.00 7.00 6.99 6.84 6.83 6.82 6.81 6.80 6.79
4.89 4.84 4.79 4.77 4.76 4.74 4.50 4.02 4.01 3.98 3.96 3.92 3.79 3.63 3.58 2.07
O
O
N
H_3C
O—CH_3
3.06 1.00 2.01 1.99 1.01 1.00 1.02 1.00 3.02 1.01 2.97
9.5 9.0 8.5 8.0 7.5 7.0 6.5 6.0 5.5 5.0 4.5 4.0 3.5 3.0 2.5 2.0 1.5 1.0 0.5 0.0 -0.5 -1.0
f1 (ppm)

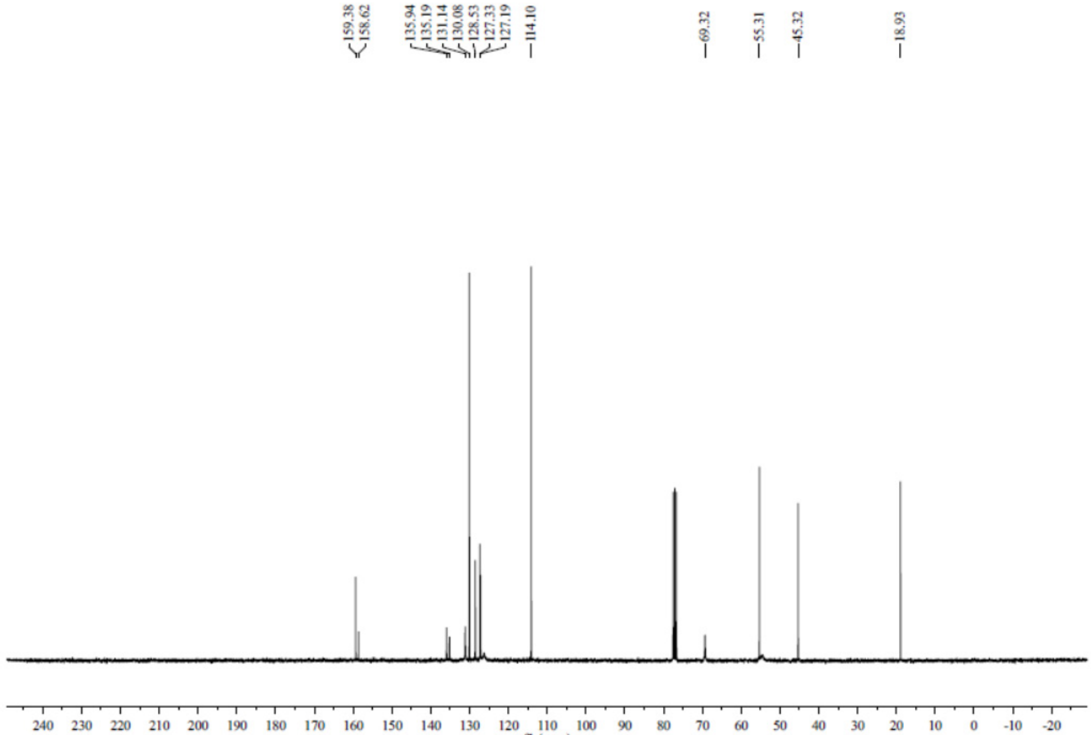
159.38 158.62 135.94 135.19 131.14 130.08 128.53 127.33 127.19 114.10 69.32 55.31 45.32 18.93
240 230 220 210 200 190 180 170 160 150 140 130 120 110 100 90 80 70 60 50 40 30 20 10 0 -10 -20
f1 (ppm)

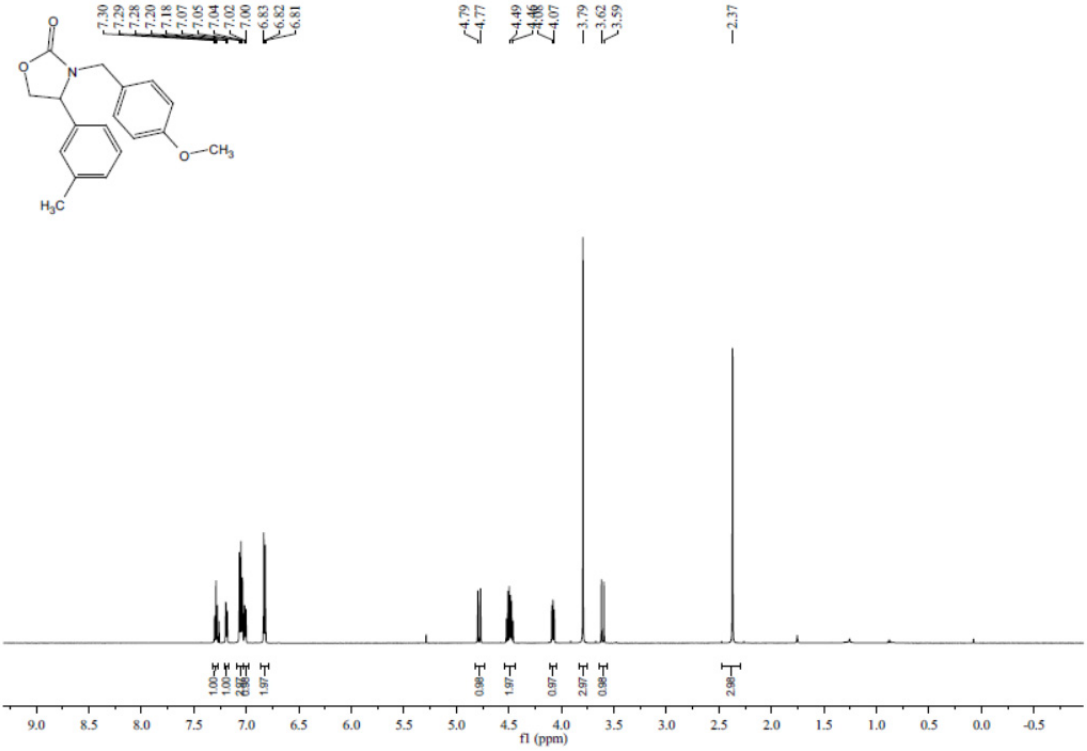
7.30
7.29
7.28
7.20
7.18
7.07
7.05
7.04
7.02
7.00
6.83
6.82
6.81
4.79
4.77
4.49
4.07
3.79
3.62
3.59
2.37
O
N
O
O—CH3
H3C
1.00
1.00
2.97
1.97
0.98
1.97
0.97
2.97
0.98
2.98
9.0 8.5 8.0 7.5 7.0 6.5 6.0 5.5 5.0 4.5 4.0 3.5 3.0 2.5 2.0 1.5 1.0 0.5 0.0 -0.5
f1 (ppm)

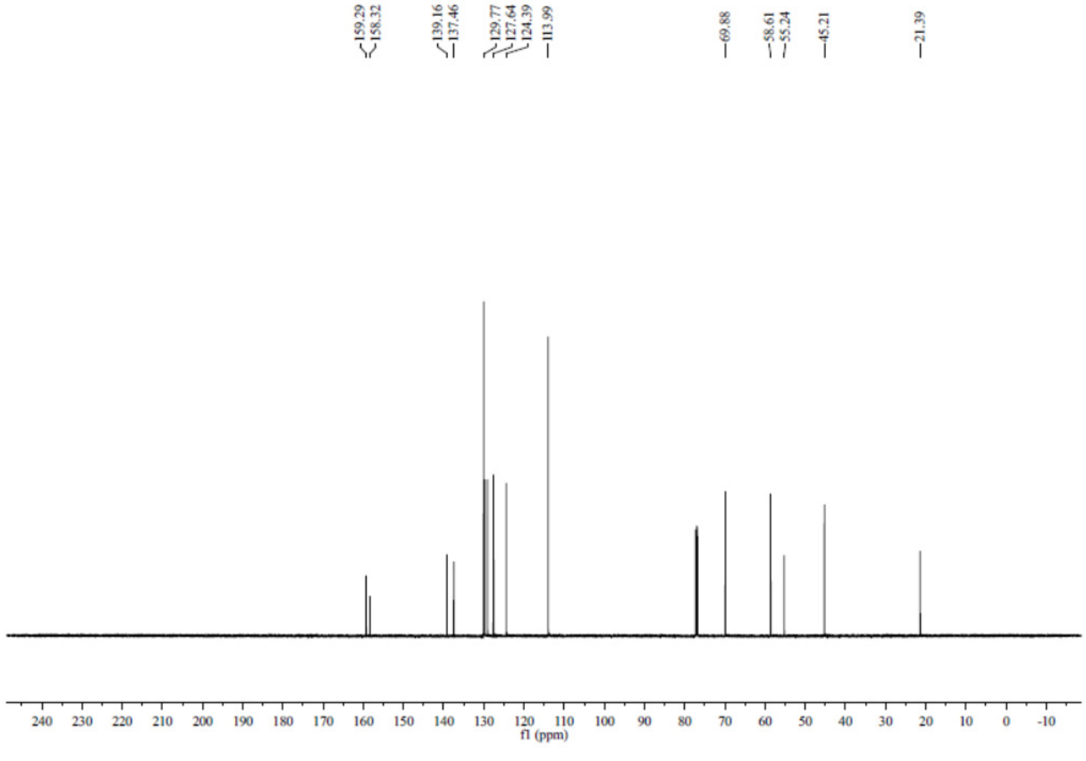
159.29
158.32
139.16
137.46
129.77
127.64
124.39
113.99
69.88
58.61
55.24
45.21
21.39
240 230 220 210 200 190 180 170 160 150 140 130 120 110 100 90 80 70 60 50 40 30 20 10 0 -10
f1 (ppm)

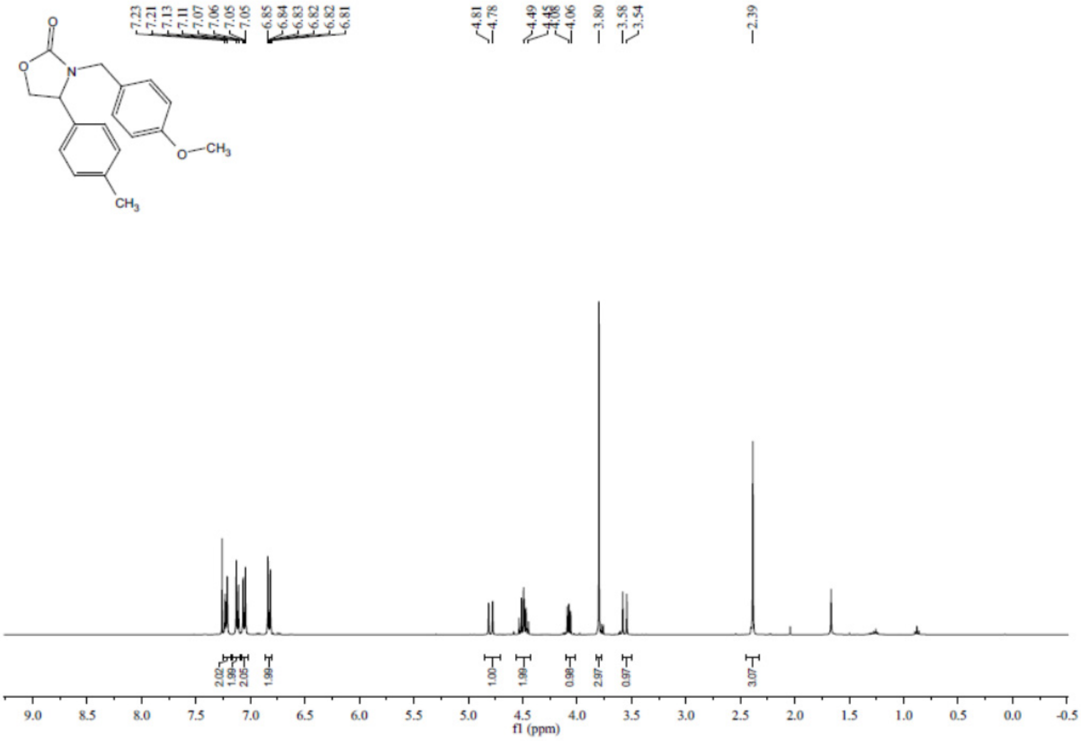
O
N
O
O
CH3
CH3
7.23
7.21
7.13
7.11
7.07
7.06
7.05
7.05
6.85
6.84
6.83
6.82
6.82
6.81
4.81
4.78
4.49
4.45
4.08
4.06
3.80
3.58
3.54
2.39
2.02
1.99
2.05
1.99
1.00
1.99
0.98
2.97
0.97
3.07
9.0 8.5 8.0 7.5 7.0 6.5 6.0 5.5 5.0 4.5 4.0 3.5 3.0 2.5 2.0 1.5 1.0 0.5 0.0 -0.5
f1 (ppm)

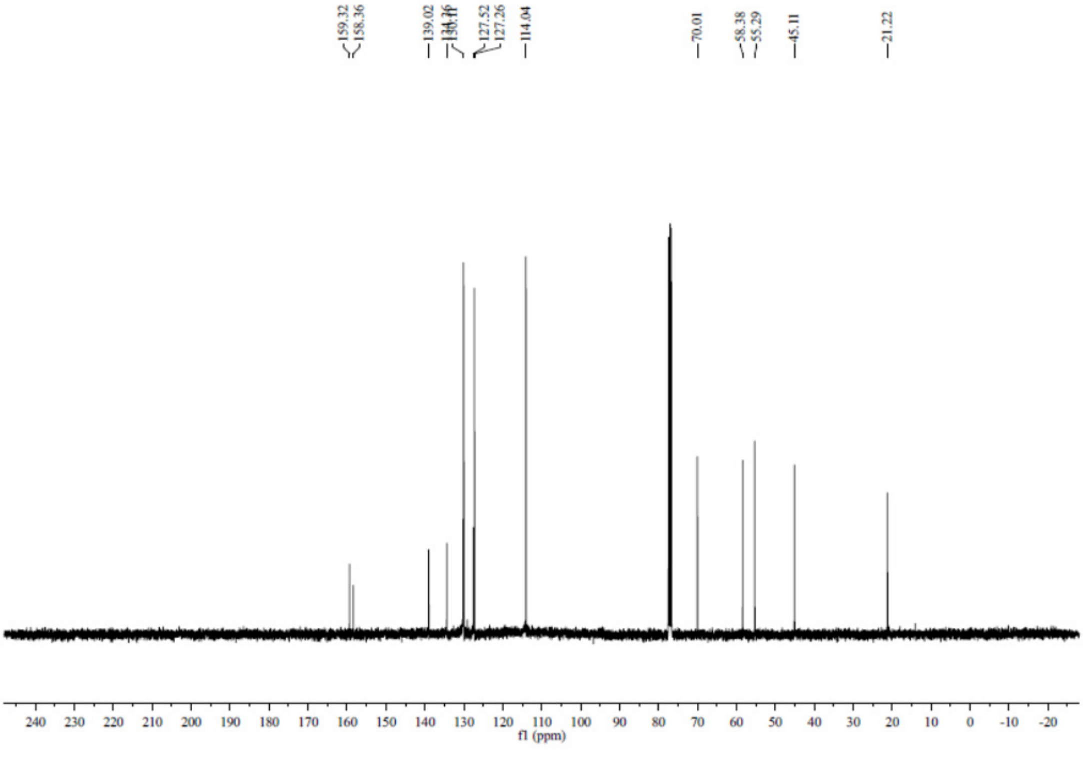
159.32
158.36
139.02
127.52
127.26
114.04
70.01
58.38
55.29
45.11
21.22
240 230 220 210 200 190 180 170 160 150 140 130 120 110 100 90 80 70 60 50 40 30 20 10 0 -10 -20
f1 (ppm)

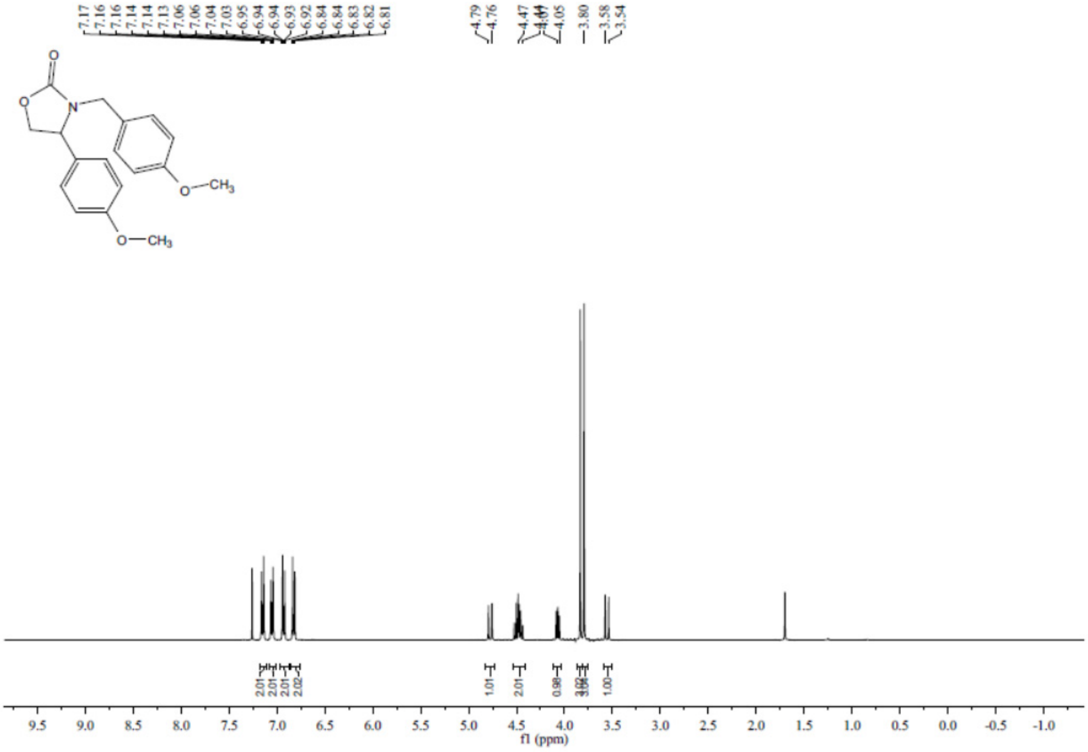
7.17
7.16
7.16
7.14
7.14
7.13
7.06
7.04
7.03
6.95
6.94
6.94
6.93
6.92
6.84
6.84
6.83
6.82
6.81
4.79
4.76
4.47
4.05
3.80
3.58
3.54
O
N
O
O—CH3
O—CH3
2.01
2.01
2.01
2.02
1.01
2.01
0.98
1.00
9.5 9.0 8.5 8.0 7.5 7.0 6.5 6.0 5.5 5.0 4.5 4.0 3.5 3.0 2.5 2.0 1.5 1.0 0.5 0.0 -0.5 -1.0
f1 (ppm)

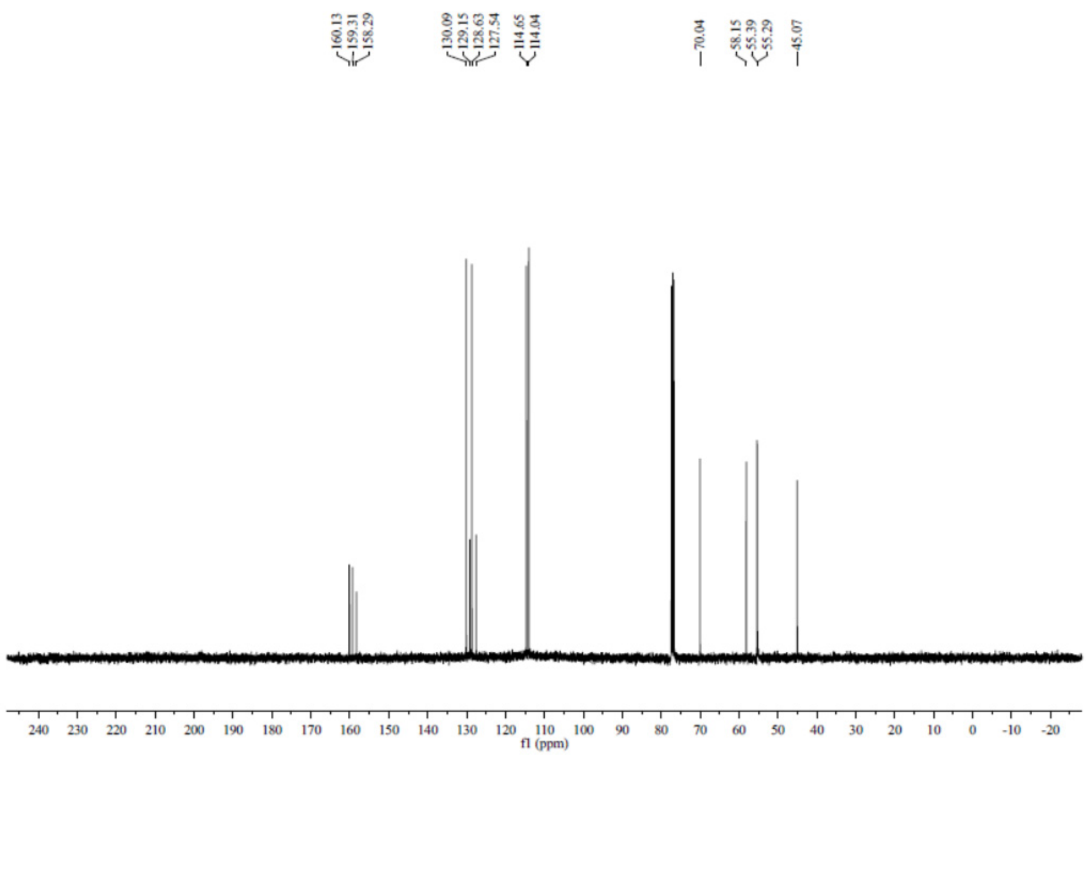
160.13
159.31
158.29
130.09
129.15
128.63
127.54
114.65
114.04
70.04
58.15
55.39
55.29
45.07
240 230 220 210 200 190 180 170 160 150 140 130 120 110 100 90 80 70 60 50 40 30 20 10 0 -10 -20
f1 (ppm)

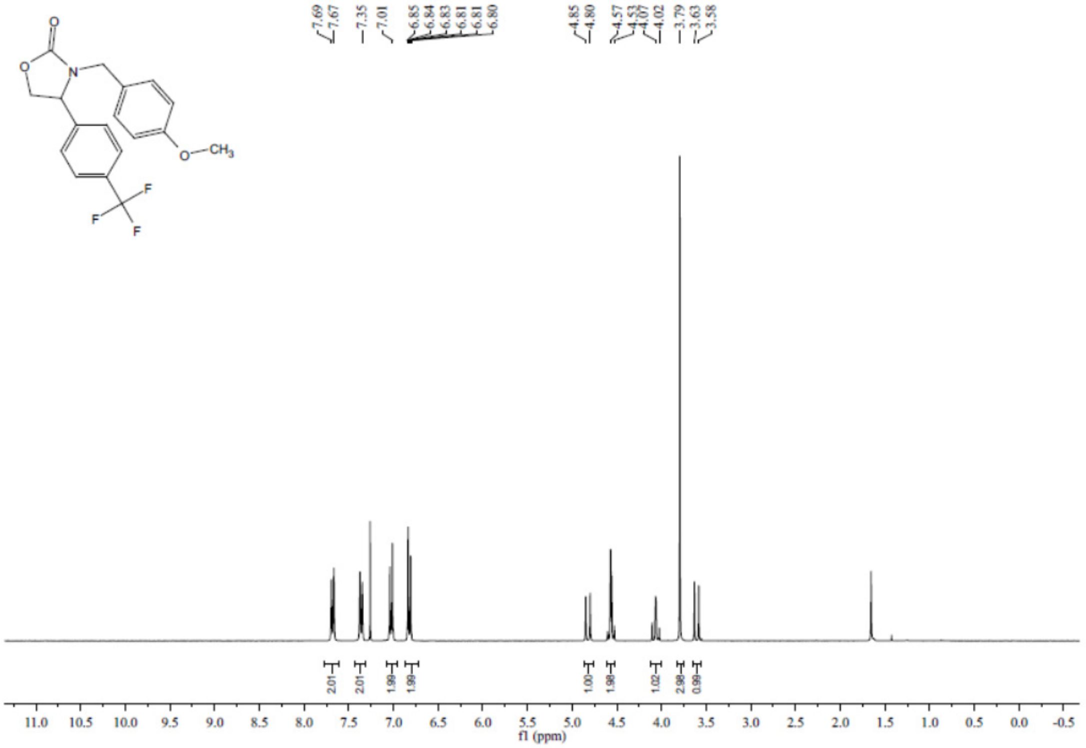
7.69
7.67
7.35
7.01
6.85
6.84
6.83
6.81
6.81
6.80
4.85
4.80
4.57
4.53
4.07
4.02
3.79
3.63
3.58
O
N
O
O
CH3
F
F
F
2.01
2.01
1.99
1.99
1.00
1.98
1.02
2.98
0.99
11.0 10.5 10.0 9.5 9.0 8.5 8.0 7.5 7.0 6.5 6.0 5.5 5.0 4.5 4.0 3.5 3.0 2.5 2.0 1.5 1.0 0.5 0.0 -0.5
f1 (ppm)

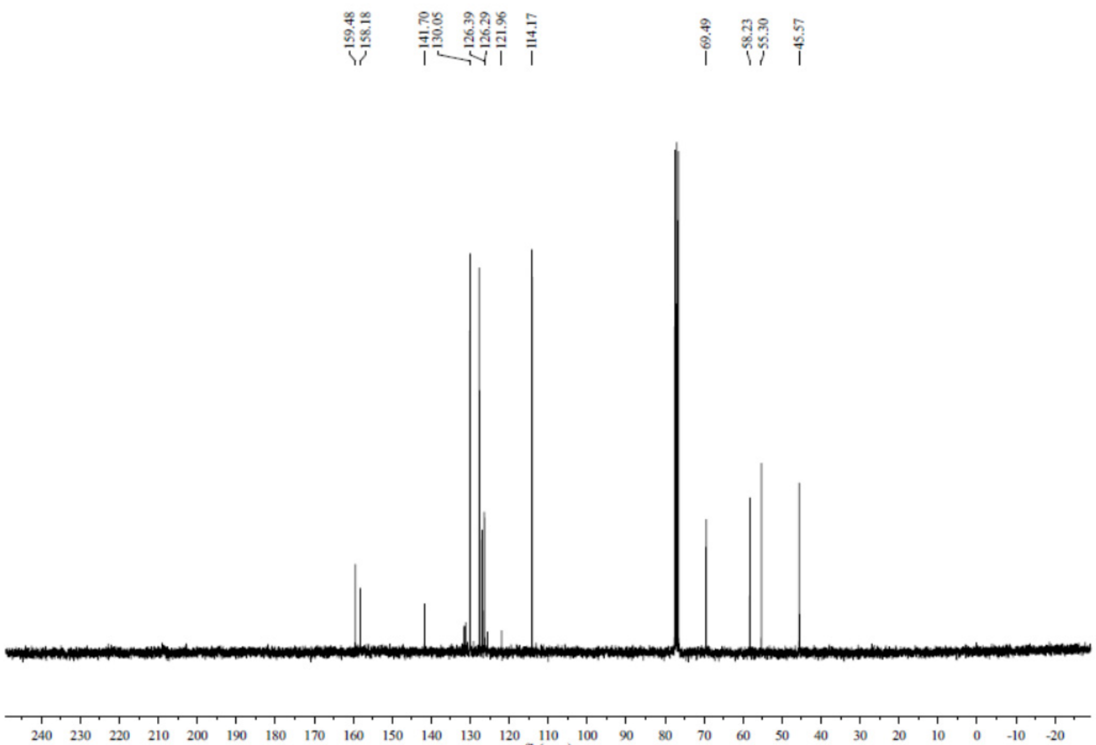
159.48
158.18
141.70
130.05
126.39
126.29
121.96
114.17
69.49
58.23
55.30
45.57
240 230 220 210 200 190 180 170 160 150 140 130 120 110 100 90 80 70 60 50 40 30 20 10 0 -10 -20
f1 (ppm)

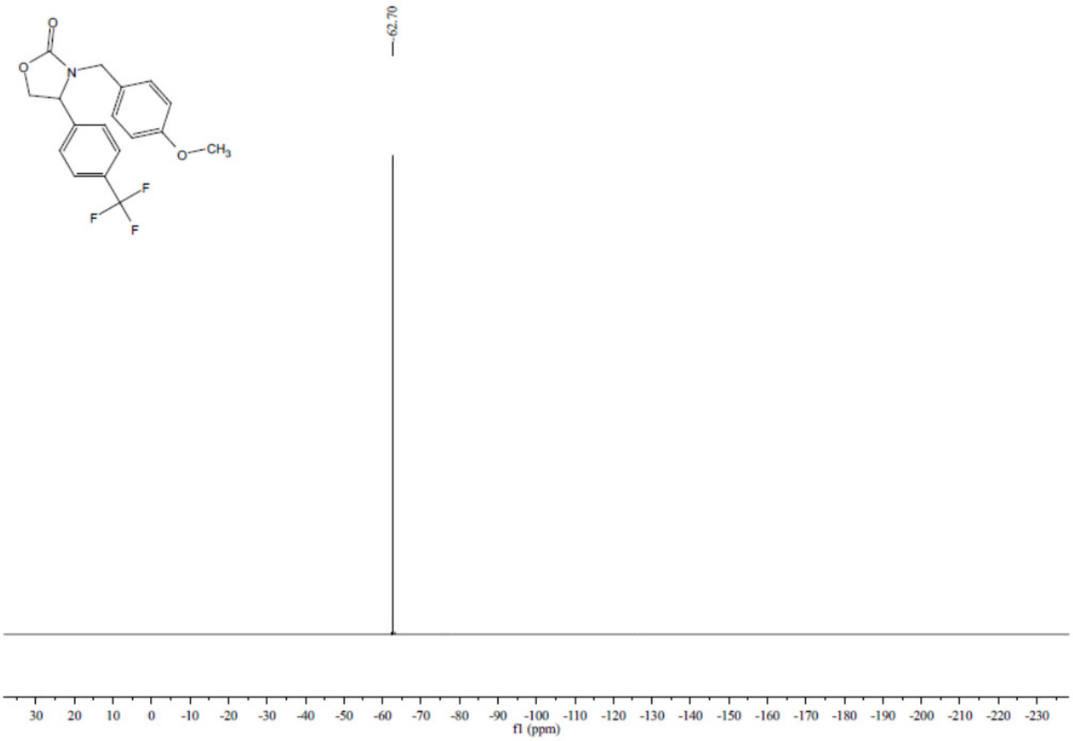

-62.70
30 20 10 0 -10 -20 -30 -40 -50 -60 -70 -80 -90 -100 -110 -120 -130 -140 -150 -160 -170 -180 -190 -200 -210 -220 -230
f1 (ppm)
O
N
O
O—CH3
F
F
F

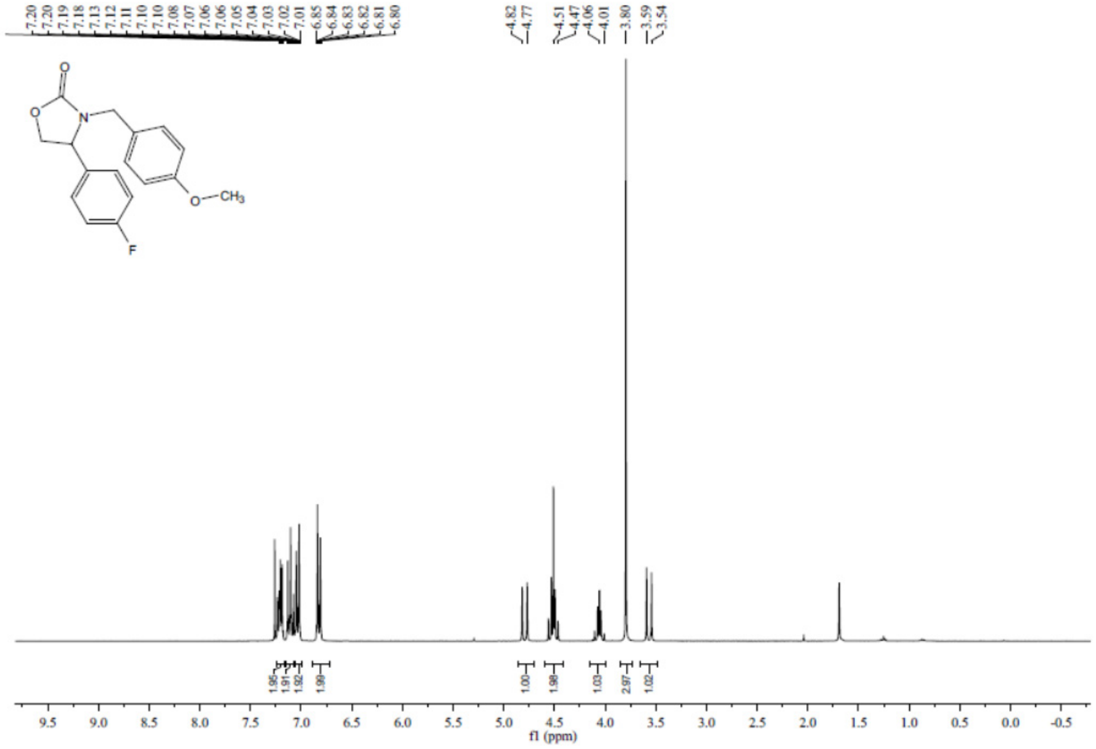
7.20
7.20
7.19
7.18
7.13
7.12
7.11
7.10
7.10
7.08
7.07
7.06
7.06
7.05
7.04
7.03
7.02
7.01
6.85
6.84
6.83
6.82
6.81
6.80
4.82
4.77
4.51
4.47
4.06
4.01
3.80
3.59
3.54
O
O
N
O
CH3
F
1.95
1.91
1.92
1.99
1.00
1.98
1.03
2.97
1.02
9.5 9.0 8.5 8.0 7.5 7.0 6.5 6.0 5.5 5.0 4.5 4.0 3.5 3.0 2.5 2.0 1.5 1.0 0.5 0.0 -0.5
f1 (ppm)

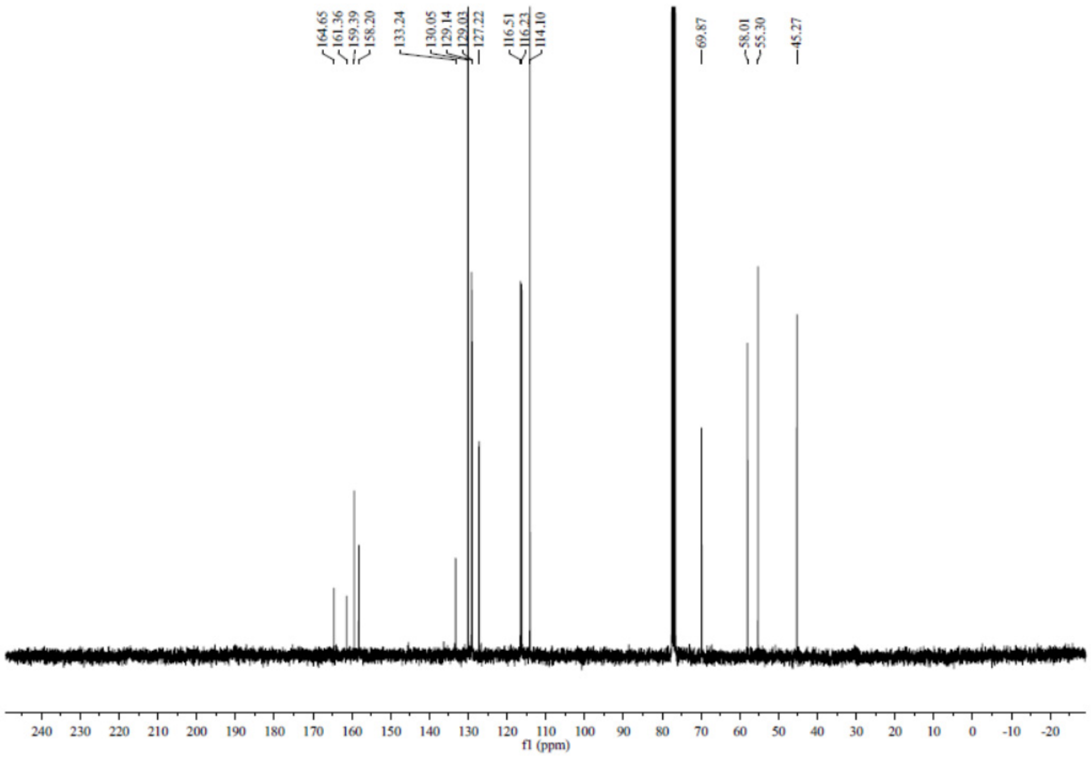
164.65
161.36
159.39
158.20
133.24
130.05
129.14
129.03
127.22
116.51
116.23
114.10
69.87
58.01
55.30
45.27
240 230 220 210 200 190 180 170 160 150 140 130 120 110 100 90 80 70 60 50 40 30 20 10 0 -10 -20
f1 (ppm)

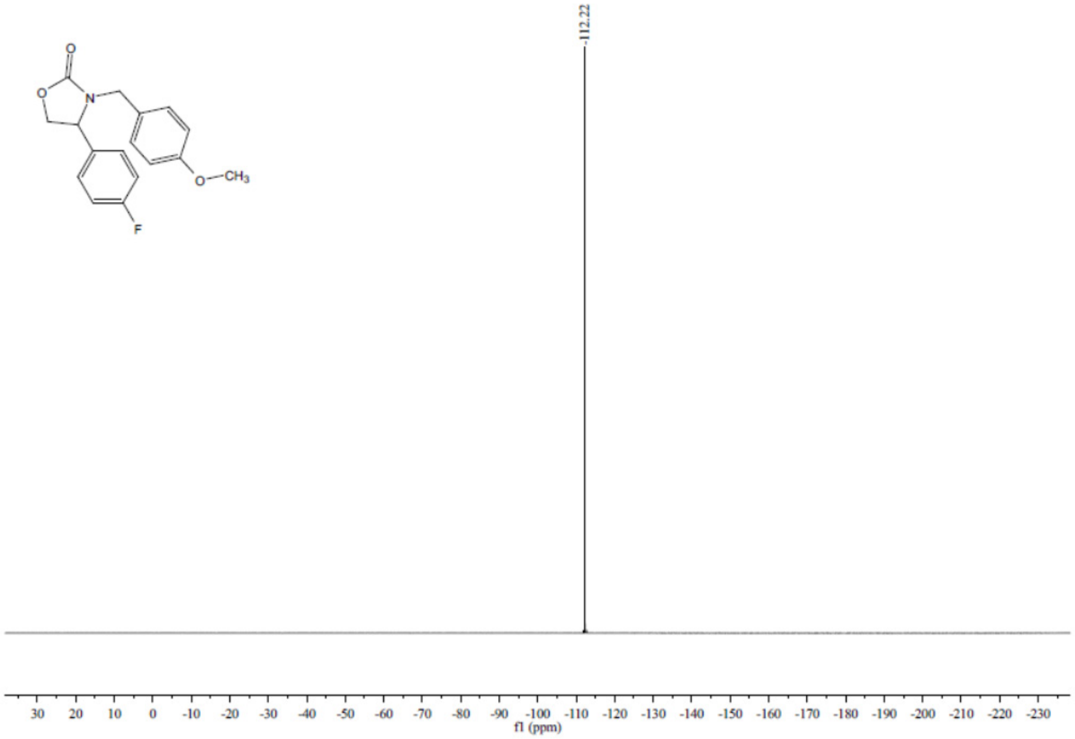

-112.22
O
N
O
O—CH3
F
30 20 10 0 -10 -20 -30 -40 -50 -60 -70 -80 -90 -100 -110 -120 -130 -140 -150 -160 -170 -180 -190 -200 -210 -220 -230
f1 (ppm)

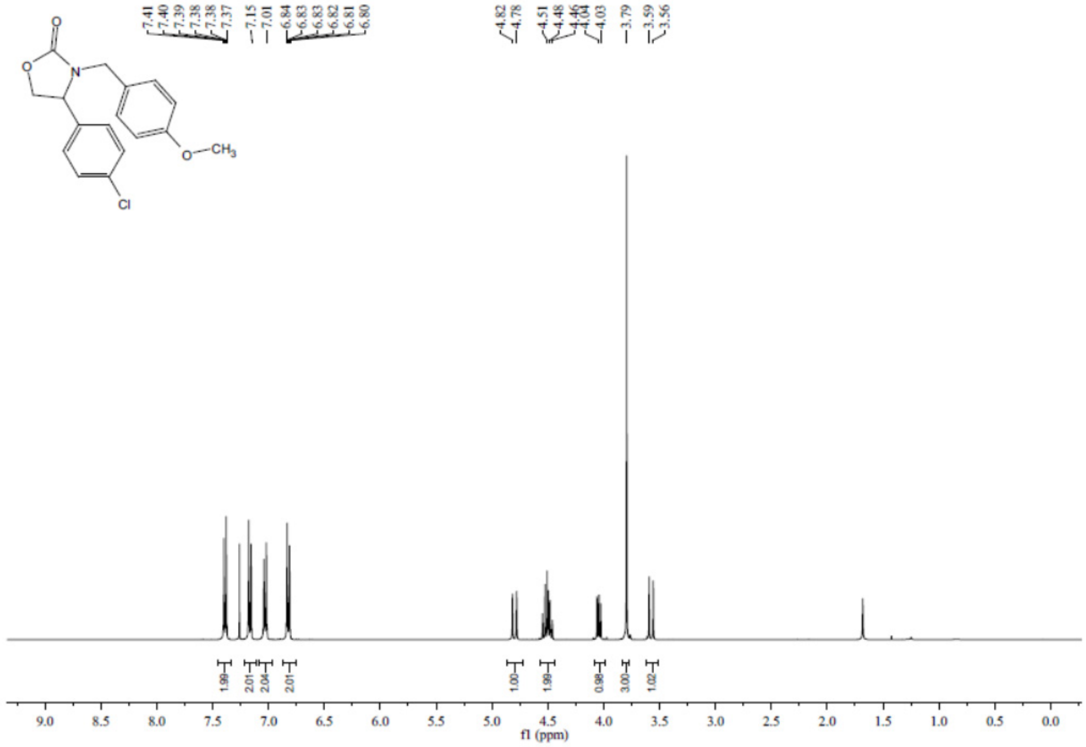

O
N
O
O—CH3
Cl
7.41
7.40
7.39
7.38
7.38
7.37
7.15
7.01
6.84
6.83
6.83
6.82
6.81
6.80
4.82
4.78
4.51
4.48
4.46
4.04
4.03
3.79
3.59
3.56
1.99
2.01
2.04
2.01
1.00
1.99
0.98
3.00
1.02
9.0 8.5 8.0 7.5 7.0 6.5 6.0 5.5 5.0 4.5 4.0 3.5 3.0 2.5 2.0 1.5 1.0 0.5 0.0
f1 (ppm)

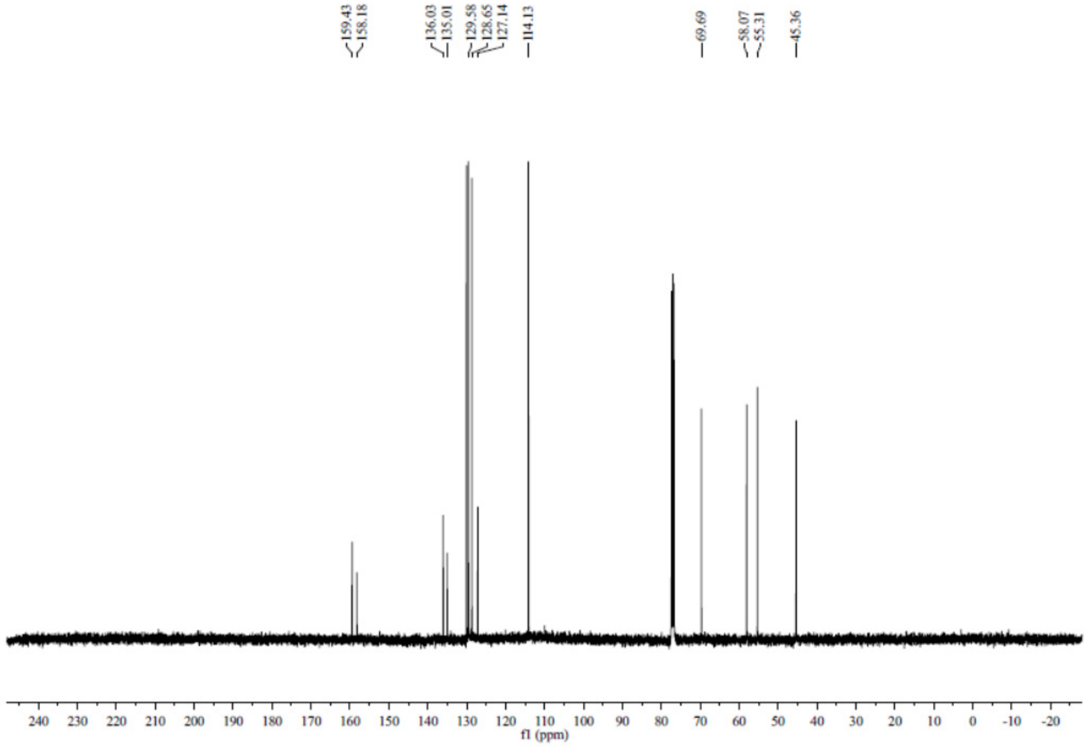

159.43
158.18
136.03
135.01
129.58
128.65
127.14
114.13
69.69
58.07
55.31
45.36
240 230 220 210 200 190 180 170 160 150 140 130 120 110 100 90 80 70 60 50 40 30 20 10 0 -10 -20
f1 (ppm)

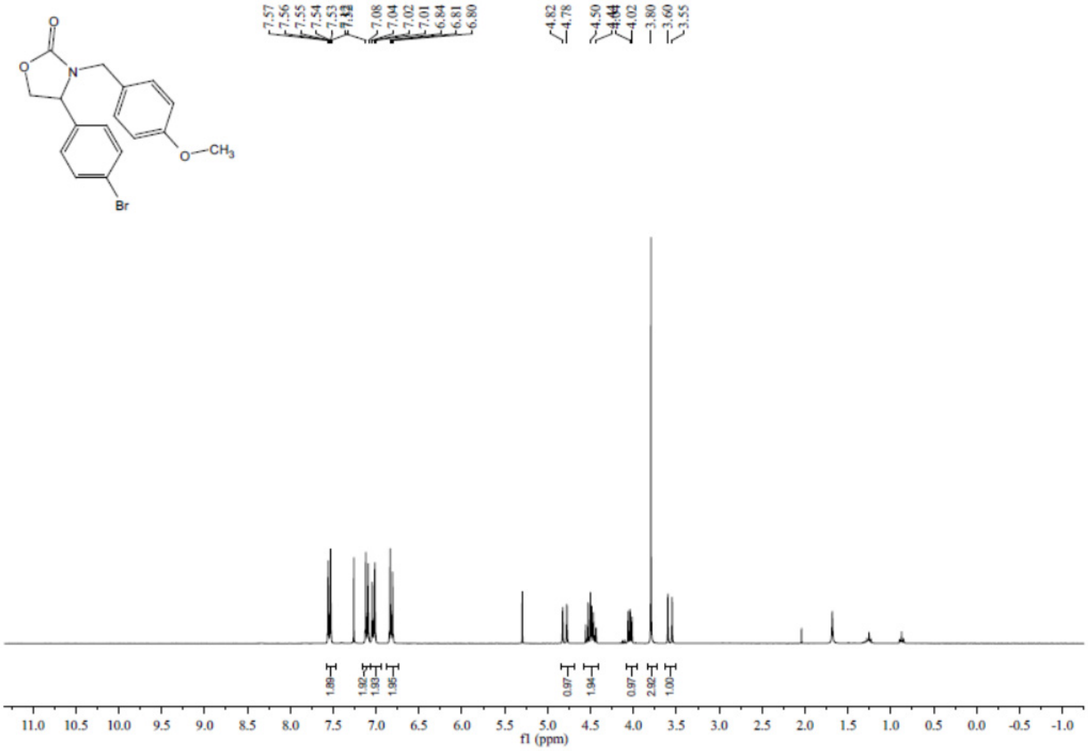
O
N
O
O—CH3
Br
7.57 7.56 7.55 7.54 7.53 7.08 7.04 7.02 7.01 6.84 6.81 6.80
4.82 4.78 4.50 4.02 3.80 3.60 3.55
1.89 1.92 1.93 1.95 0.97 1.94 0.97 2.92 1.00
11.0 10.5 10.0 9.5 9.0 8.5 8.0 7.5 7.0 6.5 6.0 5.5 5.0 4.5 4.0 3.5 3.0 2.5 2.0 1.5 1.0 0.5 0.0 -0.5 -1.0
f1 (ppm)

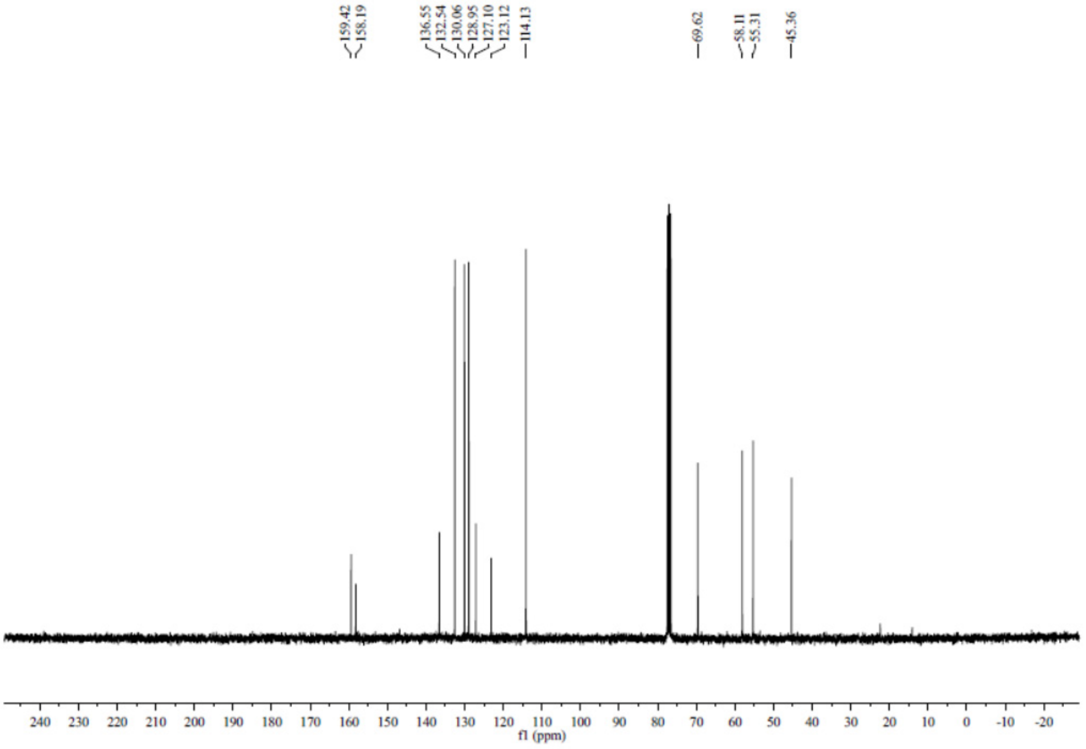
159.42 158.19
136.55 132.54 130.06 128.95 127.10 123.12 114.13
69.62 58.11 55.31 45.36
240 230 220 210 200 190 180 170 160 150 140 130 120 110 100 90 80 70 60 50 40 30 20 10 0 -10 -20
f1 (ppm)

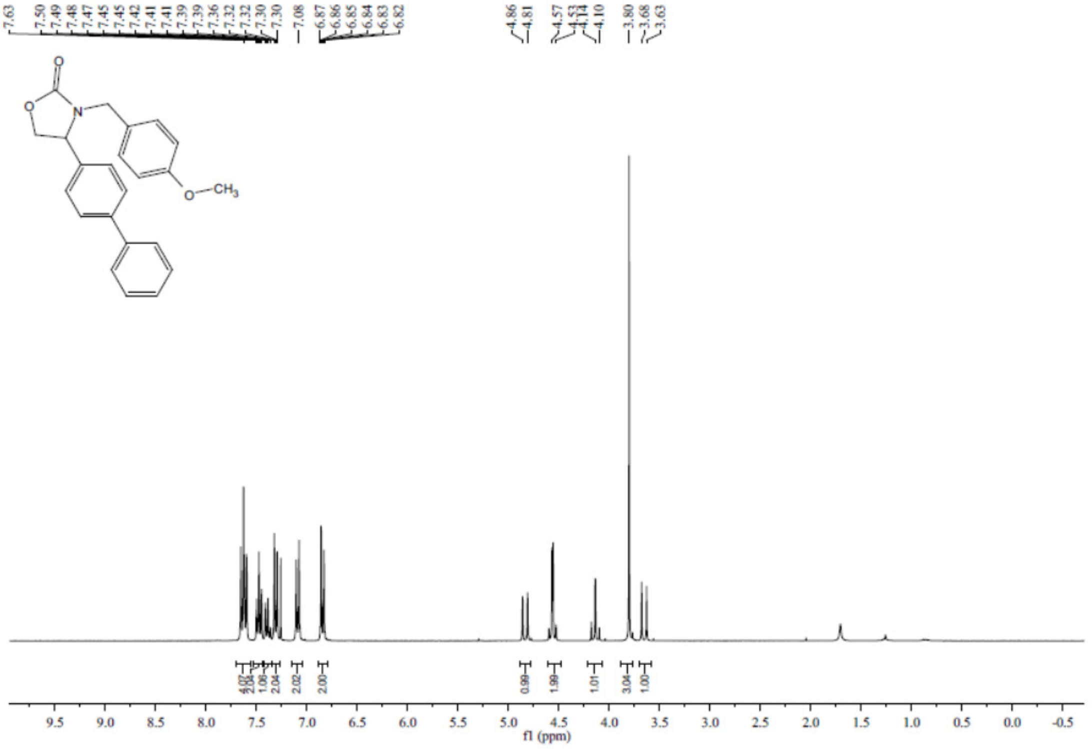
7.63
7.50
7.49
7.48
7.47
7.45
7.45
7.42
7.41
7.41
7.39
7.39
7.36
7.32
7.32
7.30
7.30
7.08
6.87
6.86
6.85
6.84
6.83
6.82
4.86
4.81
4.57
4.53
4.14
4.10
3.80
3.68
3.63
O
N
O
O
CH3
4.07
2.04
1.06
2.04
2.02
2.00
0.99
1.99
1.01
3.04
1.00
9.5 9.0 8.5 8.0 7.5 7.0 6.5 6.0 5.5 5.0 4.5 4.0 3.5 3.0 2.5 2.0 1.5 1.0 0.5 0.0 -0.5
f1 (ppm)

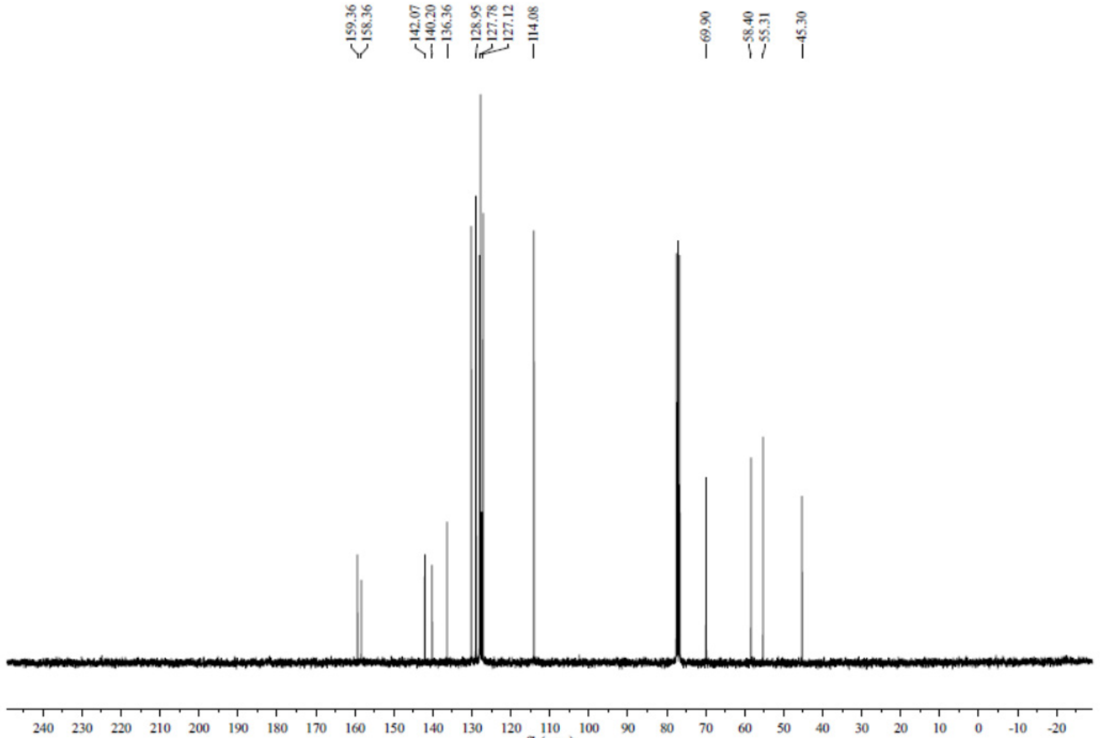
159.36
158.36
142.07
140.20
136.36
128.95
127.78
127.12
114.08
69.90
58.40
55.31
45.30
240 230 220 210 200 190 180 170 160 150 140 130 120 110 100 90 80 70 60 50 40 30 20 10 0 -10 -20
f1 (ppm)

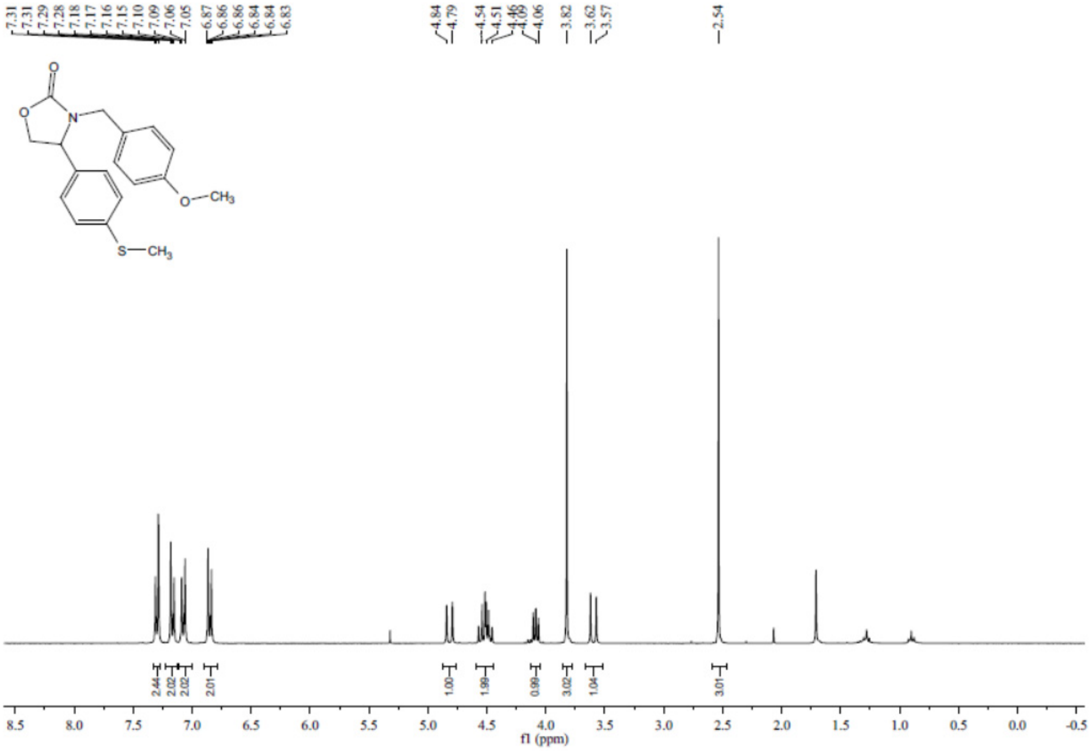
7.31
7.31
7.29
7.28
7.18
7.17
7.16
7.15
7.10
7.09
7.06
7.05
6.87
6.86
6.86
6.84
6.84
6.83
4.84
4.79
4.54
4.51
4.46
4.09
4.06
3.82
3.62
3.57
2.54
O
N
O
O
CH3
S
CH3
2.44
2.02
2.02
2.01
1.00
1.99
0.99
3.02
1.04
3.01
f1 (ppm)

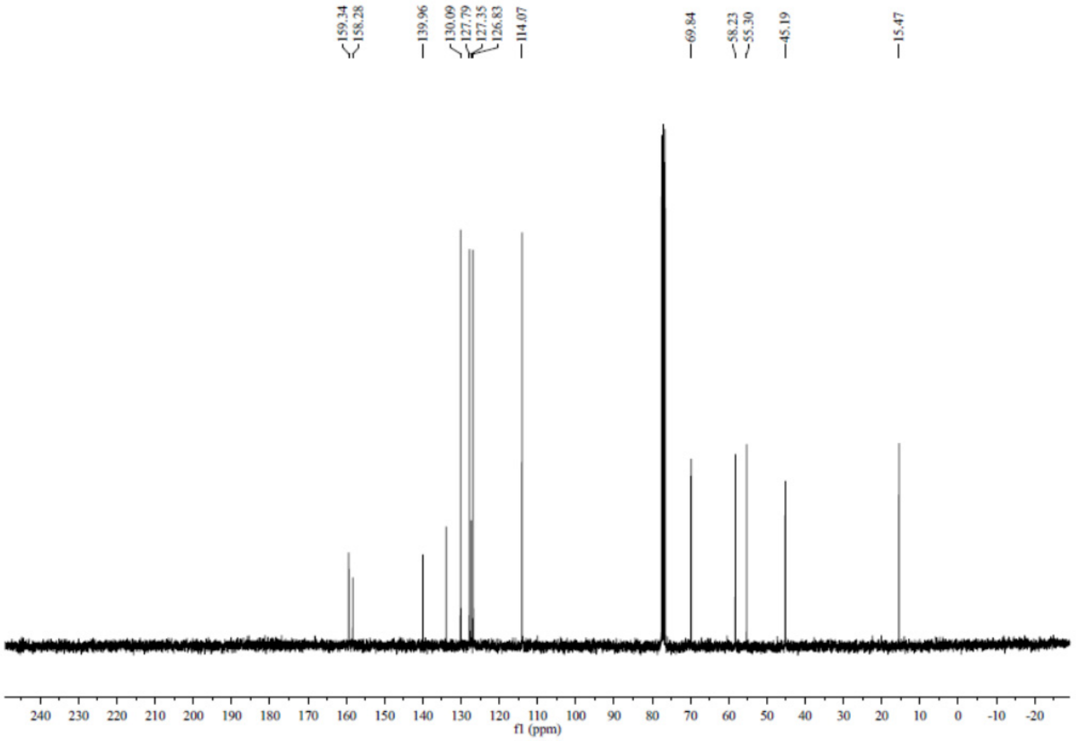
159.34
158.28
139.96
130.09
127.79
127.35
126.83
114.07
69.84
58.23
55.30
45.19
15.47
f1 (ppm)

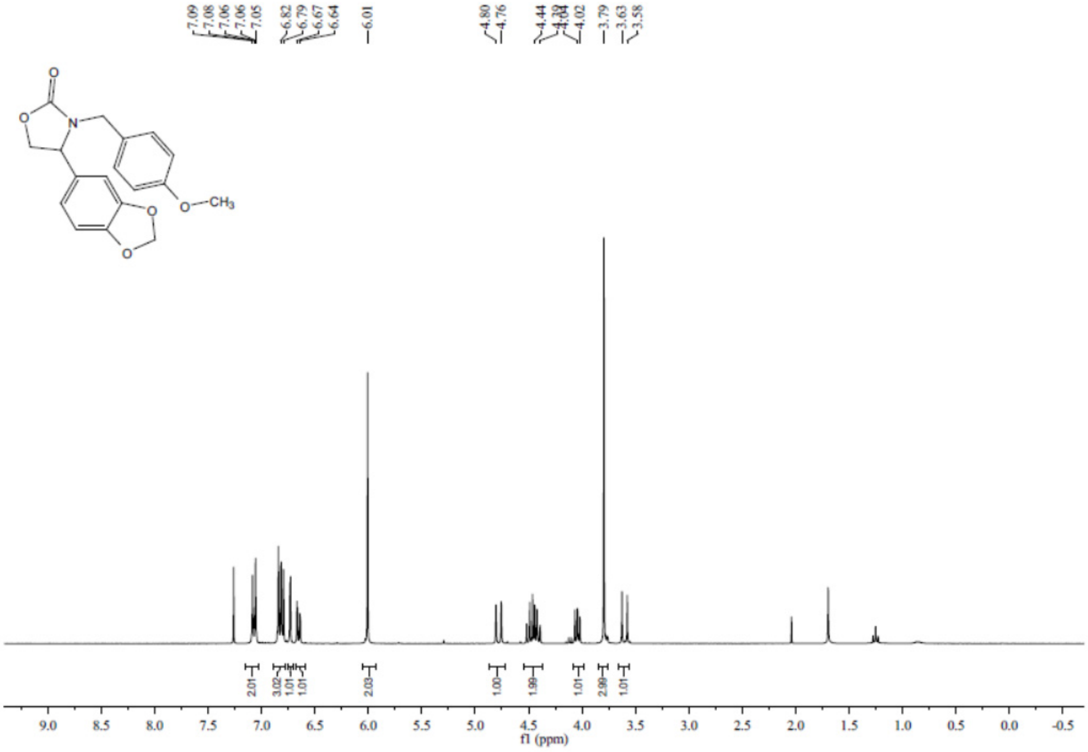

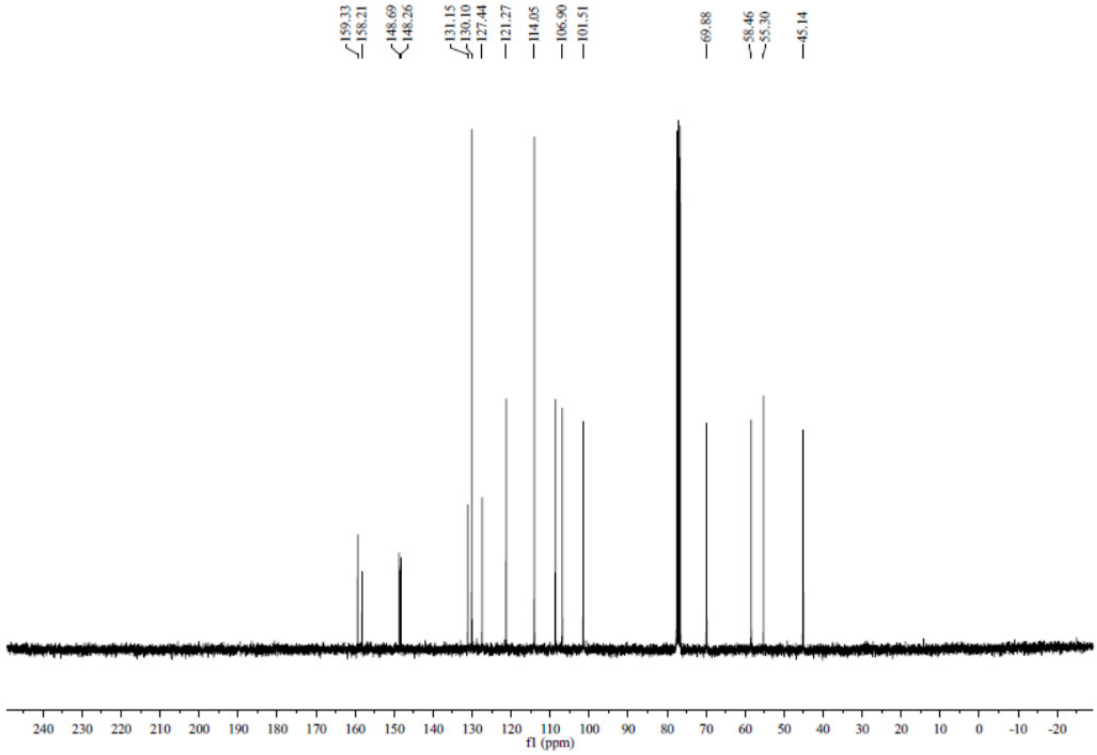

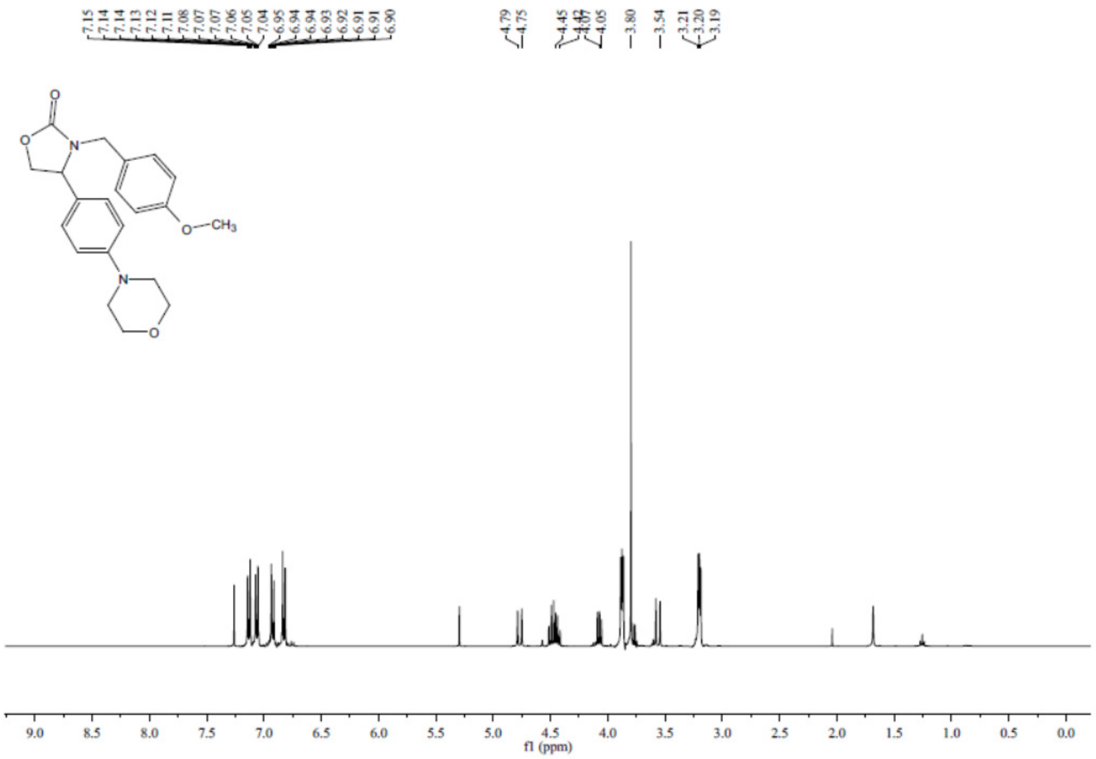
7.15
7.14
7.13
7.12
7.11
7.08
7.07
7.07
7.06
7.05
7.04
6.95
6.94
6.94
6.93
6.92
6.91
6.91
6.90
4.79
4.75
4.45
4.05
3.80
3.54
3.21
3.20
3.19
O
N
O—CH3
N
O
9.0 8.5 8.0 7.5 7.0 6.5 6.0 5.5 5.0 4.5 4.0 3.5 3.0 2.5 2.0 1.5 1.0 0.5 0.0
f1 (ppm)

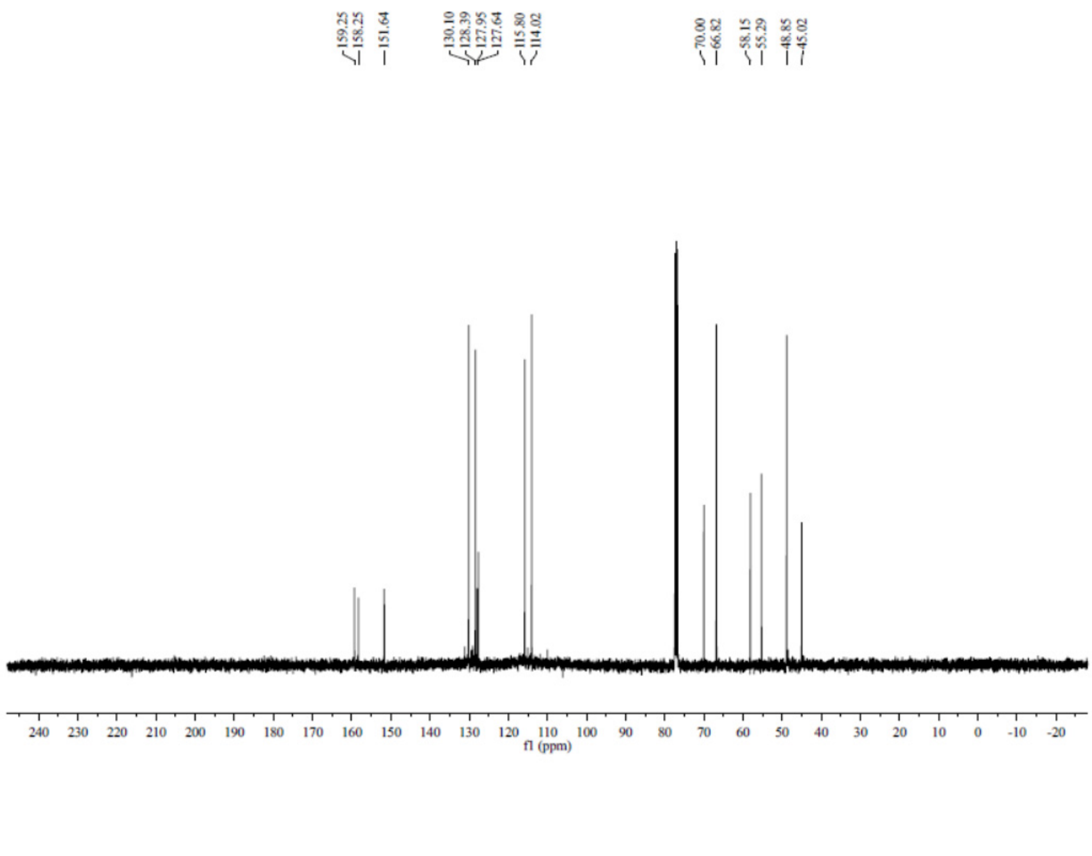
159.25
158.25
151.64
130.10
128.39
127.95
127.64
115.80
114.02
70.00
66.82
58.15
55.29
48.85
45.02
240 230 220 210 200 190 180 170 160 150 140 130 120 110 100 90 80 70 60 50 40 30 20 10 0 -10 -20
f1 (ppm)

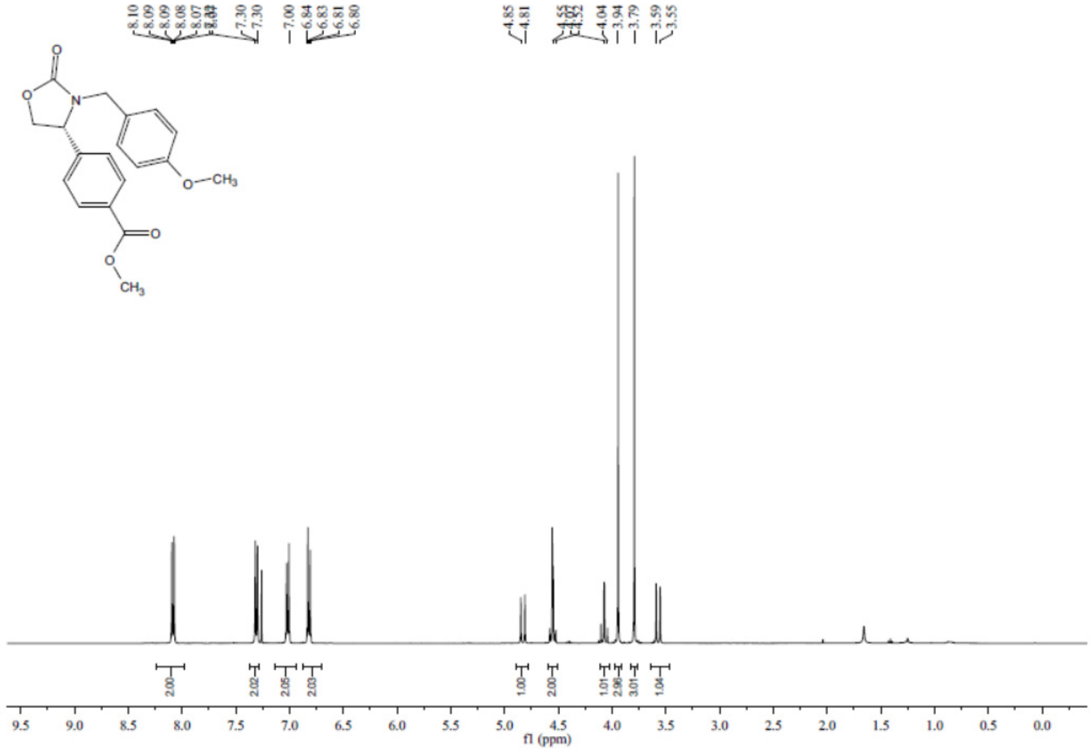
O
N
O
O—CH3
O
O
CH3
8.10
8.09
8.09
8.08
8.07
7.30
7.30
7.00
6.84
6.83
6.81
6.80
4.85
4.81
4.55
4.52
4.04
3.94
3.79
3.59
3.55
2.00
2.02
2.05
2.03
1.00
2.00
1.01
2.98
3.01
1.04
9.5 9.0 8.5 8.0 7.5 7.0 6.5 6.0 5.5 5.0 4.5 4.0 3.5 3.0 2.5 2.0 1.5 1.0 0.5 0.0
f1 (ppm)

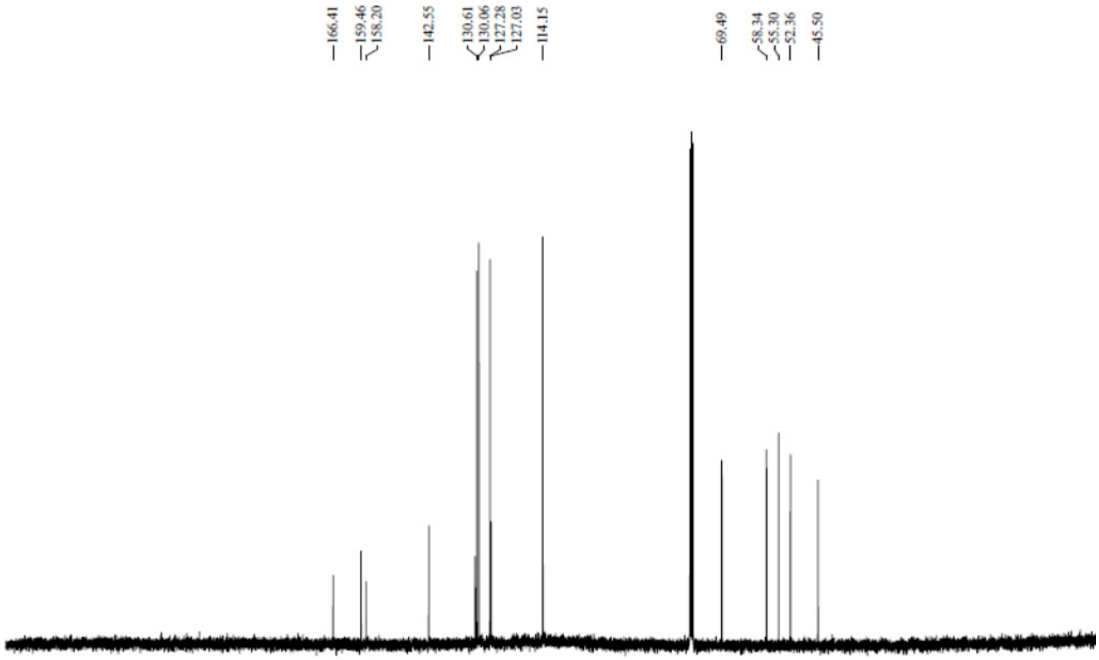
166.41
159.46
158.20
142.55
130.61
130.06
127.28
127.03
114.15
69.49
58.34
55.30
52.36
45.50

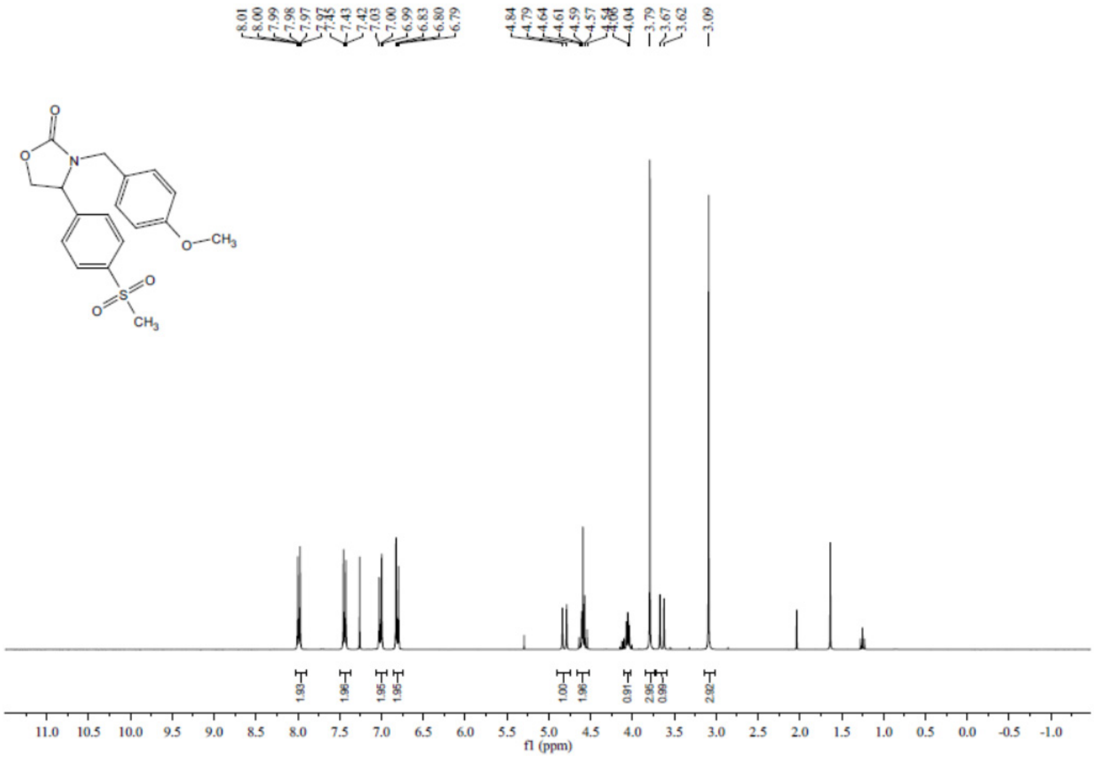
8.01
8.00
7.99
7.98
7.97
7.45
7.43
7.42
7.03
7.00
6.99
6.83
6.80
6.79
4.84
4.79
4.64
4.61
4.59
4.57
4.04
3.79
3.67
3.62
3.09
O
N
O
O—CH3
S
O
O
CH3
1.93
1.98
1.95
1.95
1.00
1.98
0.91
2.95
0.99
2.92
f1 (ppm)

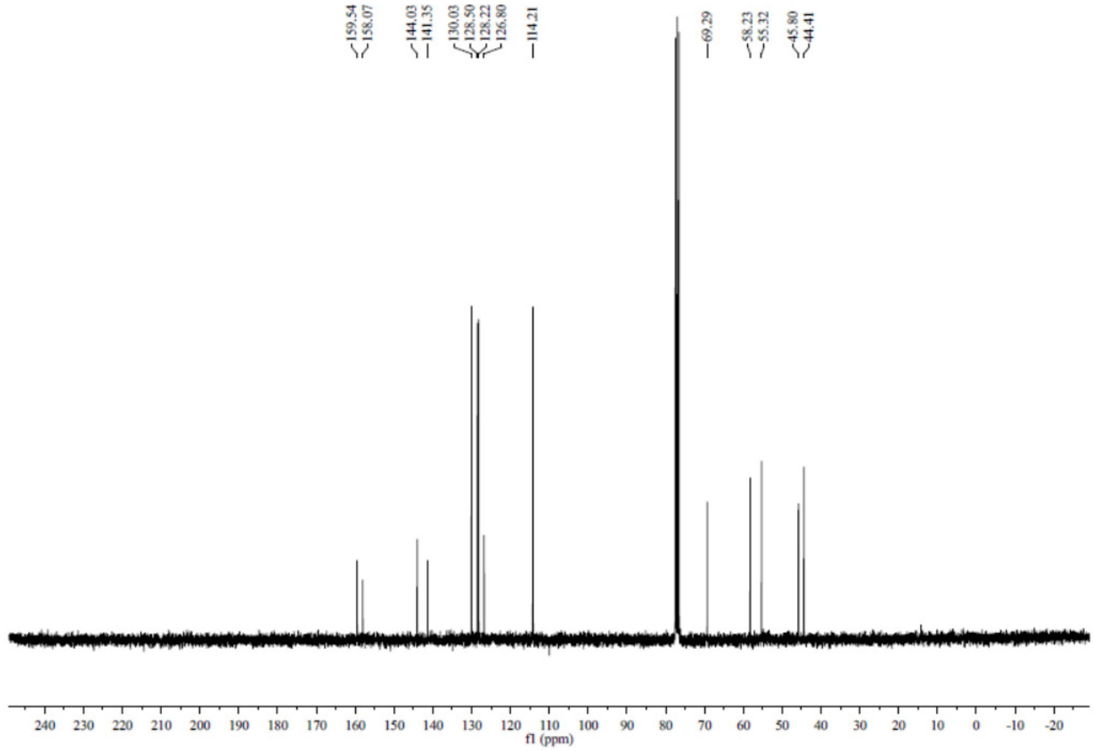
159.54
158.07
144.03
141.35
130.03
128.50
128.22
126.80
114.21
69.29
58.23
55.32
45.80
44.41
f1 (ppm)

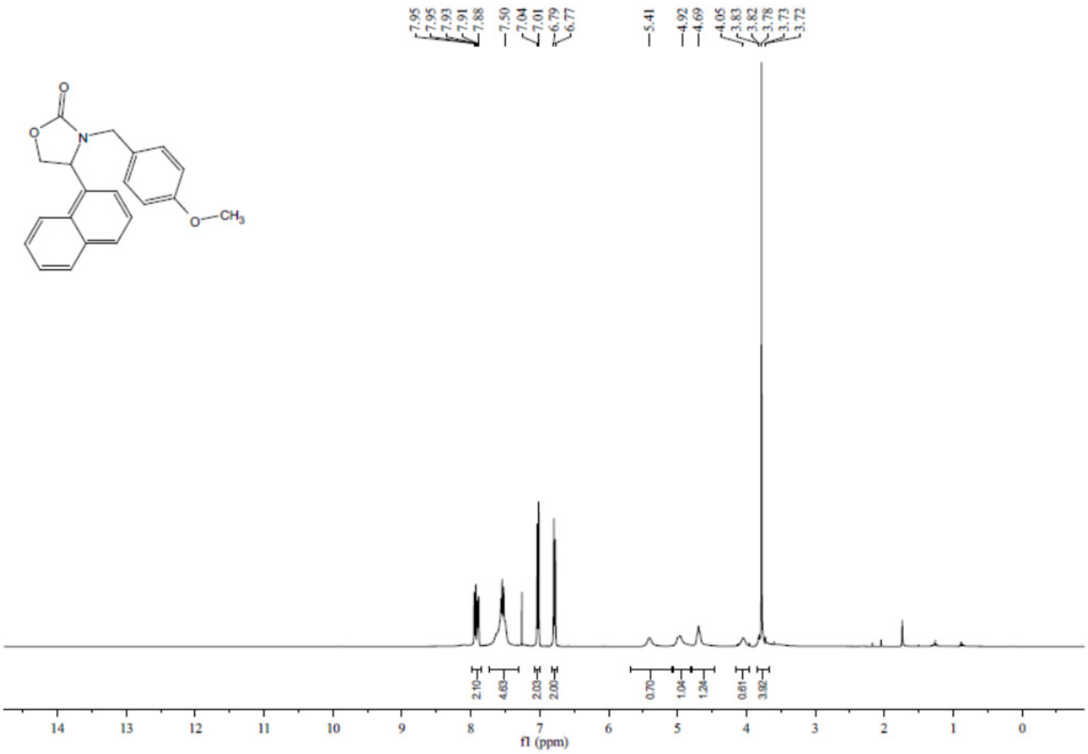
O
O
N
O
CH3
7.95
7.95
7.93
7.91
7.88
7.50
7.04
7.01
6.79
6.77
5.41
4.92
4.69
4.05
3.83
3.82
3.78
3.73
3.72
2.10
4.63
2.03
2.00
0.70
1.04
1.24
0.61
3.92
f1 (ppm)

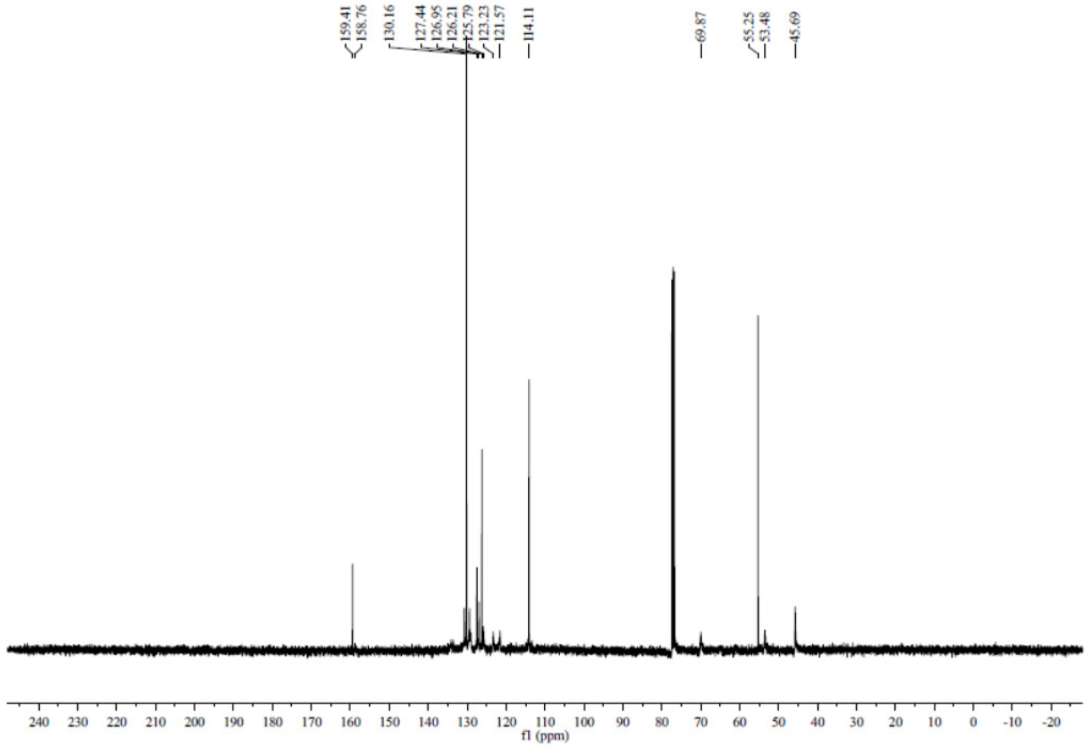
159.41
158.76
130.16
127.44
126.95
126.21
125.79
123.23
121.57
114.11
69.87
55.25
53.48
45.69
f1 (ppm)

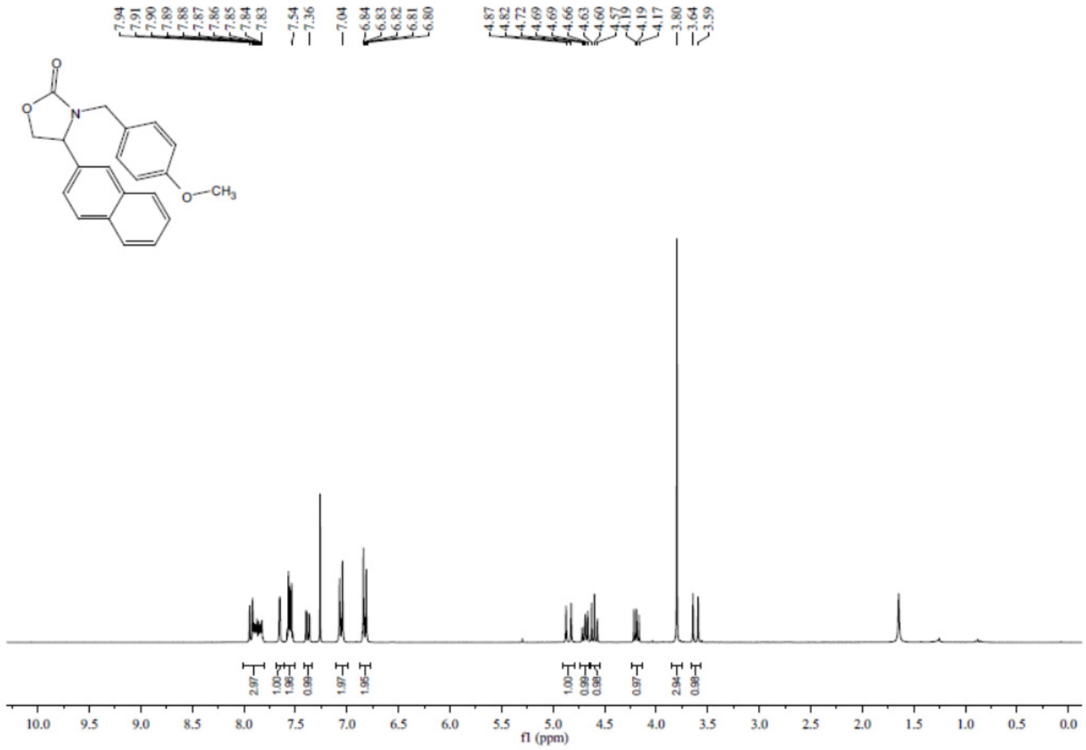
7.94
7.91
7.90
7.89
7.88
7.87
7.86
7.85
7.84
7.83
7.54
7.36
7.04
6.84
6.83
6.82
6.81
6.80
4.87
4.82
4.72
4.69
4.69
4.66
4.63
4.60
4.57
4.19
4.19
4.17
3.80
3.64
3.59
O
N
O
O
CH3
2.97
1.00
1.98
0.99
1.97
1.95
1.00
0.99
0.98
0.97
2.94
0.98
10.0 9.5 9.0 8.5 8.0 7.5 7.0 6.5 6.0 5.5 5.0 4.5 4.0 3.5 3.0 2.5 2.0 1.5 1.0 0.5 0.0
f1 (ppm)

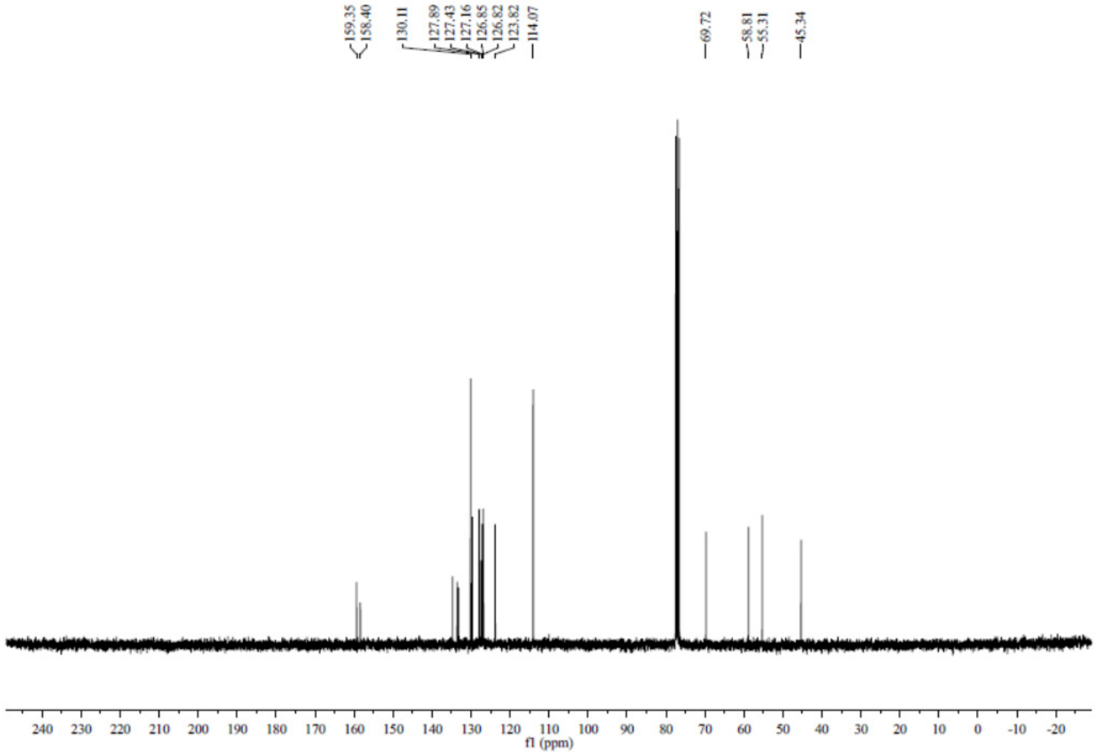
159.35
158.40
130.11
127.89
127.43
127.16
126.85
126.82
123.82
114.07
69.72
58.81
55.31
45.34
240 230 220 210 200 190 180 170 160 150 140 130 120 110 100 90 80 70 60 50 40 30 20 10 0 -10 -20
f1 (ppm)

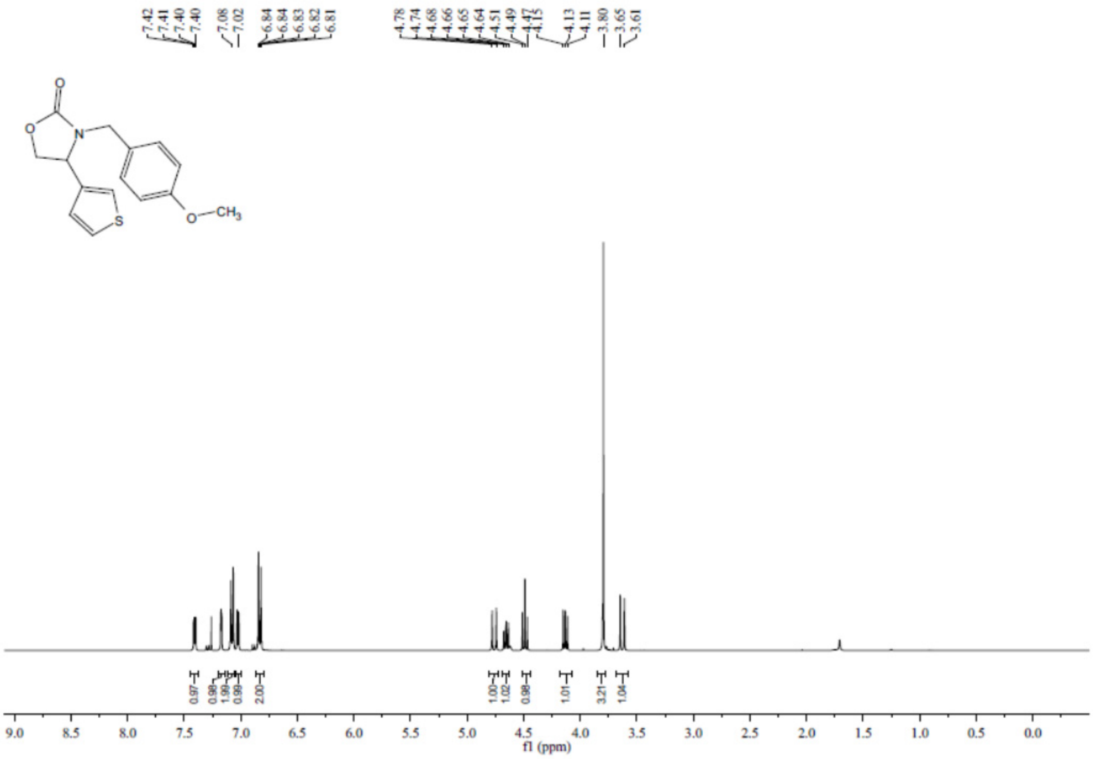
7.42
7.41
7.40
7.40
7.08
7.02
6.84
6.84
6.83
6.82
6.81
4.78
4.74
4.68
4.66
4.65
4.64
4.51
4.49
4.47
4.15
4.13
4.11
3.80
3.65
3.61
O
N
S
O—CH3
0.97
0.98
1.99
0.99
2.00
1.00
1.02
0.98
1.01
3.21
1.04
9.0 8.5 8.0 7.5 7.0 6.5 6.0 5.5 5.0 4.5 4.0 3.5 3.0 2.5 2.0 1.5 1.0 0.5 0.0
f1 (ppm)

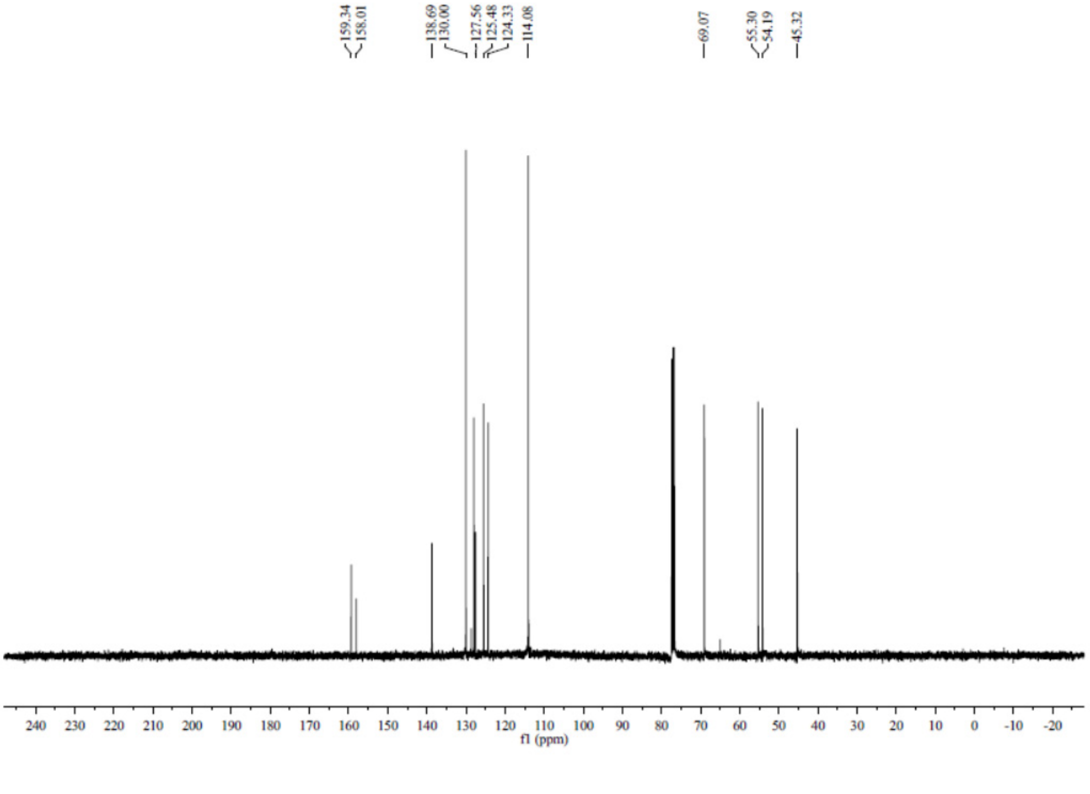
159.34
158.01
138.69
130.00
127.56
125.48
124.33
114.08
69.07
55.30
54.19
45.32
240 230 220 210 200 190 180 170 160 150 140 130 120 110 100 90 80 70 60 50 40 30 20 10 0 -10 -20
f1 (ppm)

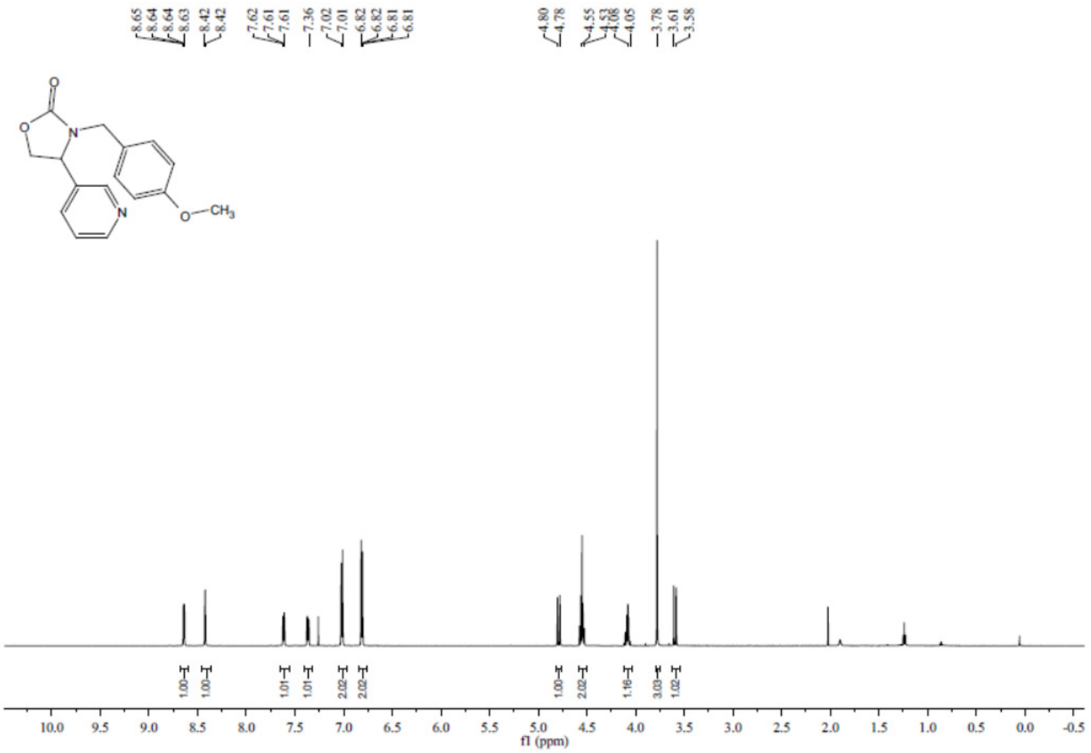
8.65
8.64
8.64
8.63
8.42
8.42
7.62
7.61
7.61
7.36
7.02
7.01
6.82
6.82
6.81
6.81
4.80
4.78
4.55
4.53
4.08
4.05
3.78
3.61
3.58
O
N
N
O
CH3
1.00
1.00
1.01
1.01
2.02
2.02
1.00
2.02
1.16
3.03
1.02
10.0 9.5 9.0 8.5 8.0 7.5 7.0 6.5 6.0 5.5 5.0 4.5 4.0 3.5 3.0 2.5 2.0 1.5 1.0 0.5 0.0 -0.5
f1 (ppm)

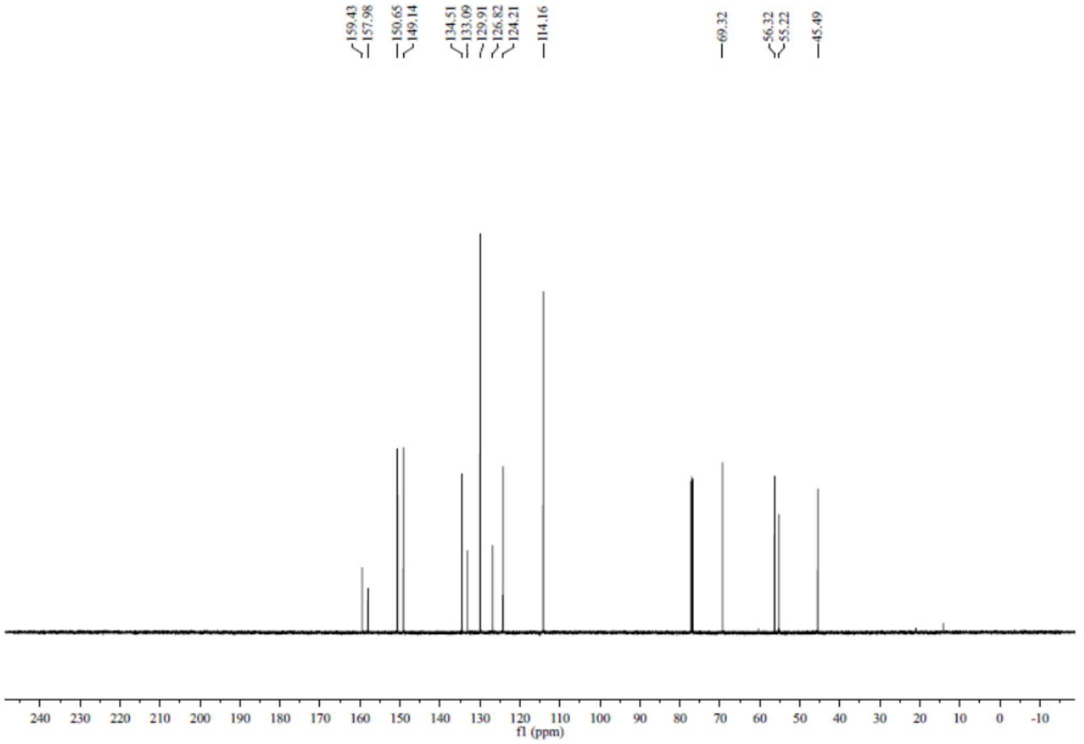
159.43
157.98
150.65
149.14
134.51
133.09
129.91
126.82
124.21
114.16
69.32
56.32
55.22
45.49
240 230 220 210 200 190 180 170 160 150 140 130 120 110 100 90 80 70 60 50 40 30 20 10 0 -10
f1 (ppm)

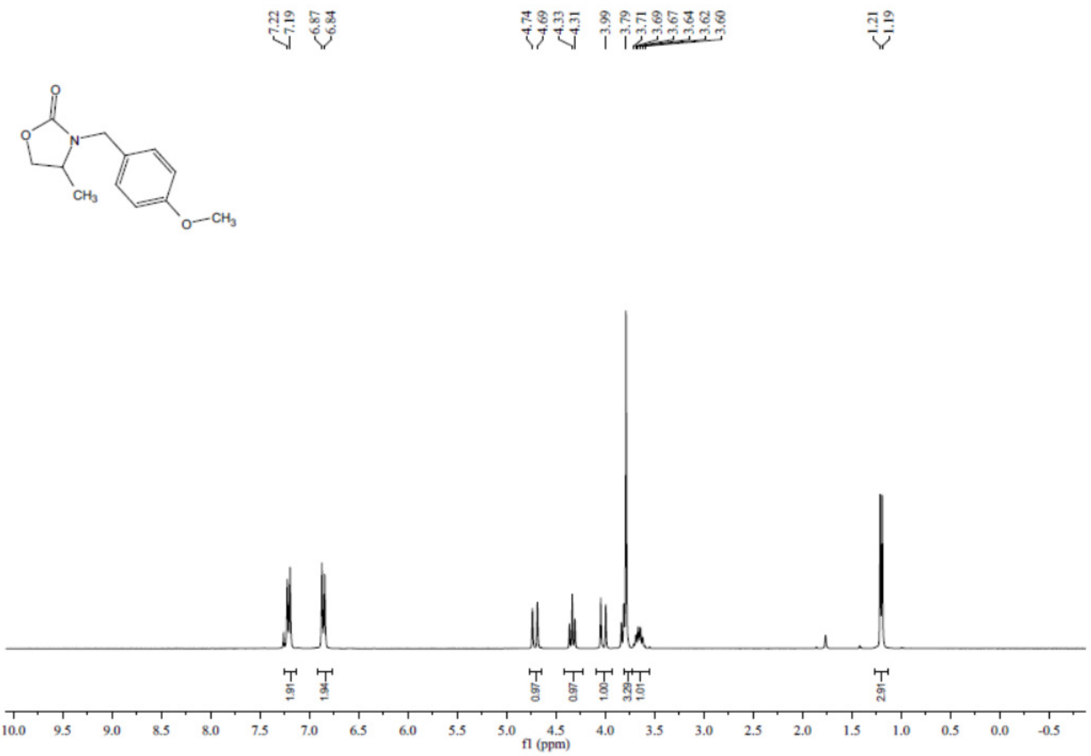
7.22
7.19
6.87
6.84
4.74
4.69
4.33
4.31
3.99
3.79
3.71
3.69
3.67
3.64
3.62
3.60
1.21
1.19
1.91
1.94
0.97
0.97
1.00
3.29
1.01
2.91
10.0 9.5 9.0 8.5 8.0 7.5 7.0 6.5 6.0 5.5 5.0 4.5 4.0 3.5 3.0 2.5 2.0 1.5 1.0 0.5 0.0 -0.5
f1 (ppm)

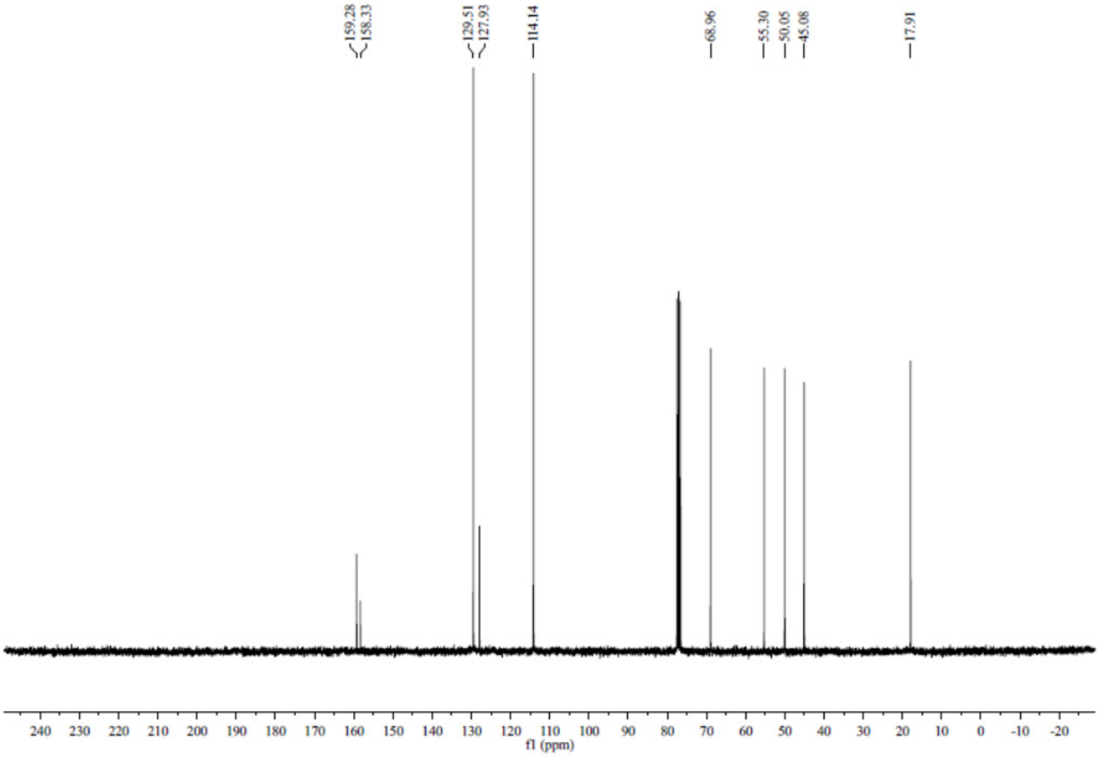
159.28
158.33
129.51
127.93
114.14
68.96
55.30
50.05
45.08
17.91
240 230 220 210 200 190 180 170 160 150 140 130 120 110 100 90 80 70 60 50 40 30 20 10 0 -10 -20
f1 (ppm)

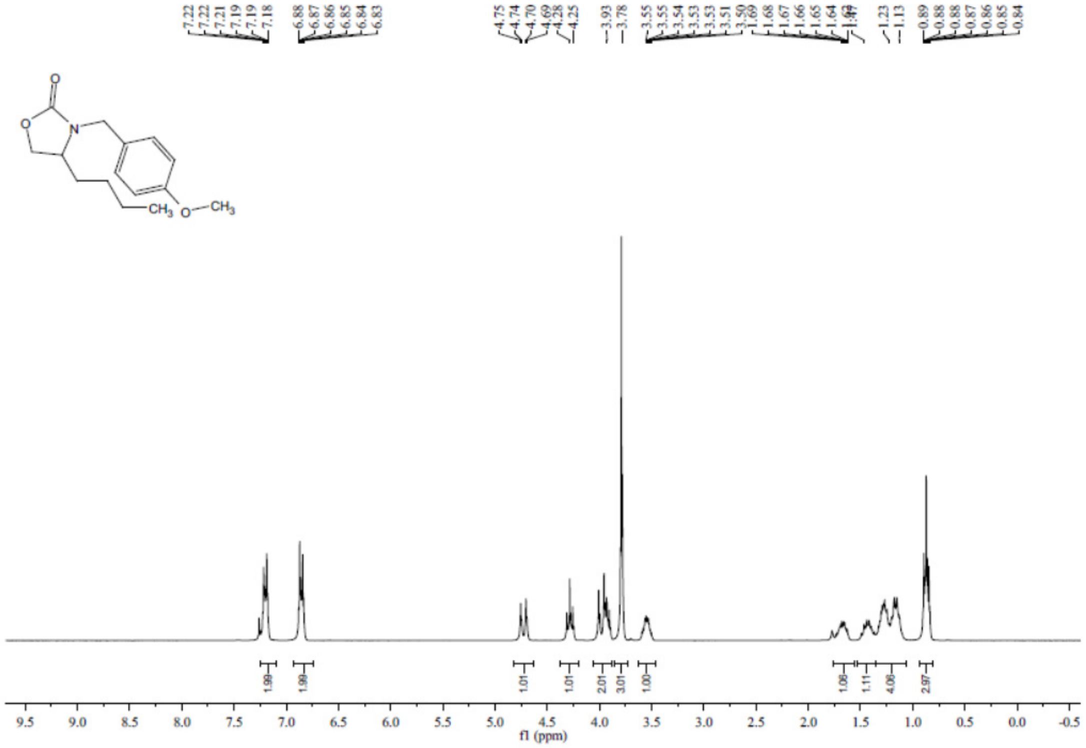
O
N
O
CH3
O
CH3
f1 (ppm)

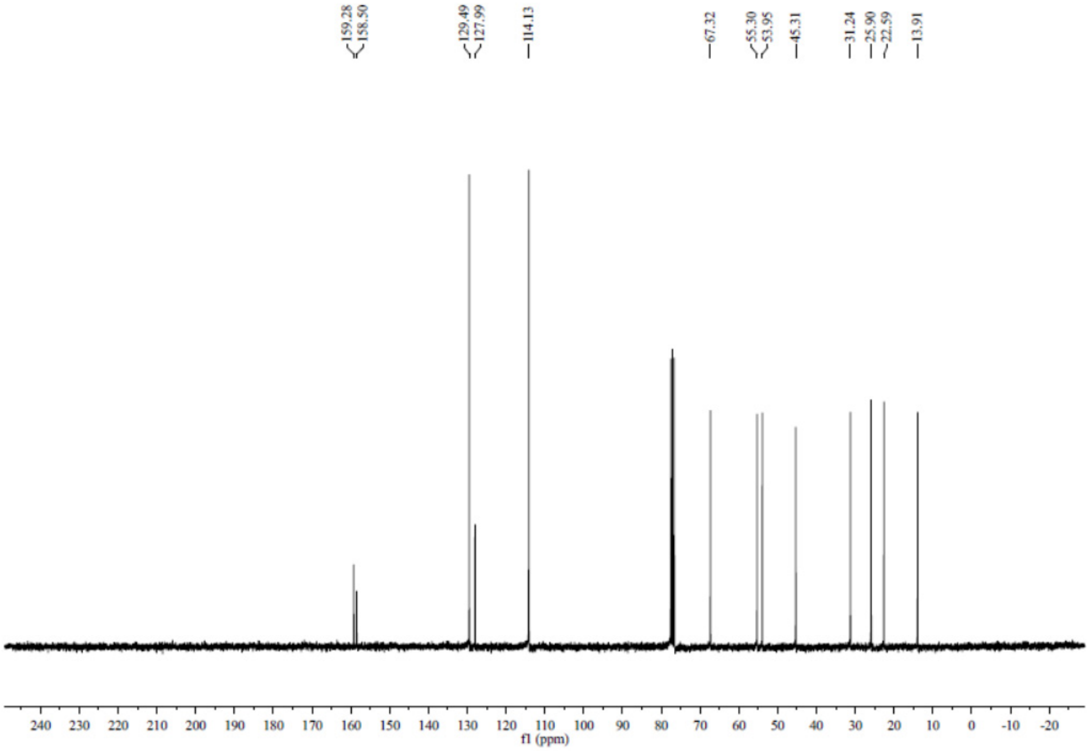
159.28
158.50
129.49
127.99
114.13
67.32
55.30
53.95
45.31
31.24
25.90
22.59
13.91
f1 (ppm)

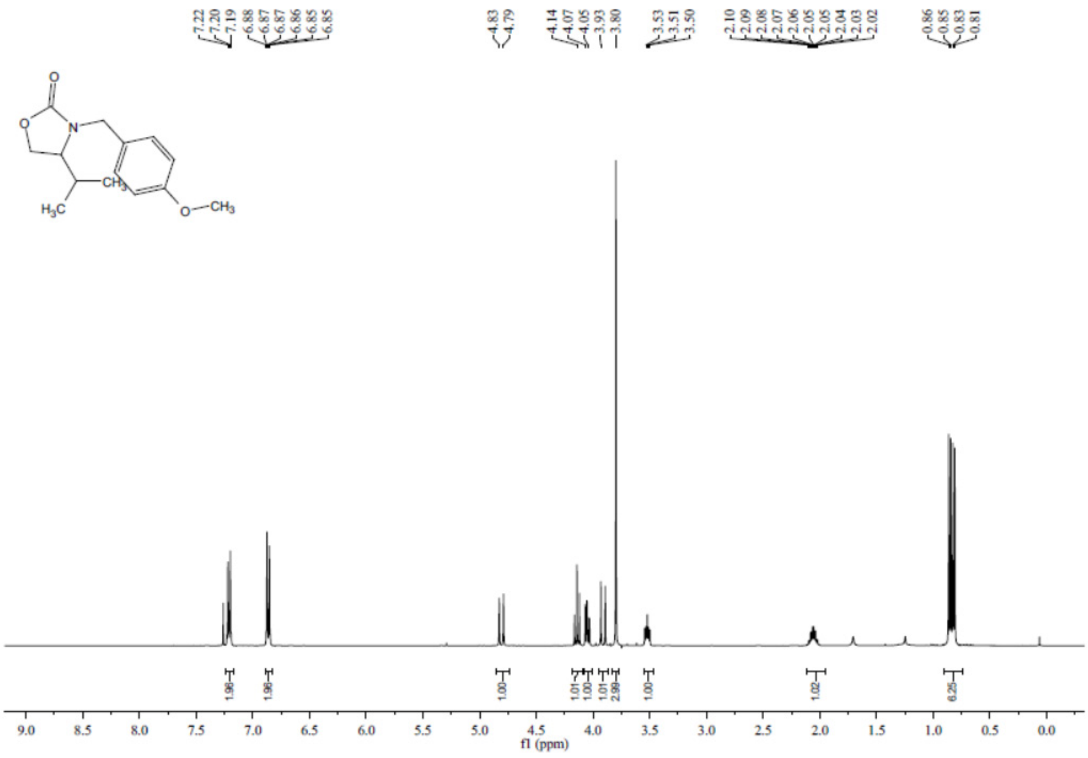
7.22
7.20
7.19
6.88
6.87
6.87
6.86
6.85
6.85
4.83
4.79
4.14
4.07
4.05
3.93
3.80
3.53
3.51
3.50
2.10
2.09
2.08
2.07
2.06
2.05
2.05
2.04
2.03
2.02
0.86
0.85
0.83
0.81
1.96
1.96
1.00
1.01
1.00
1.01
2.99
1.00
1.02
6.25
f1 (ppm)

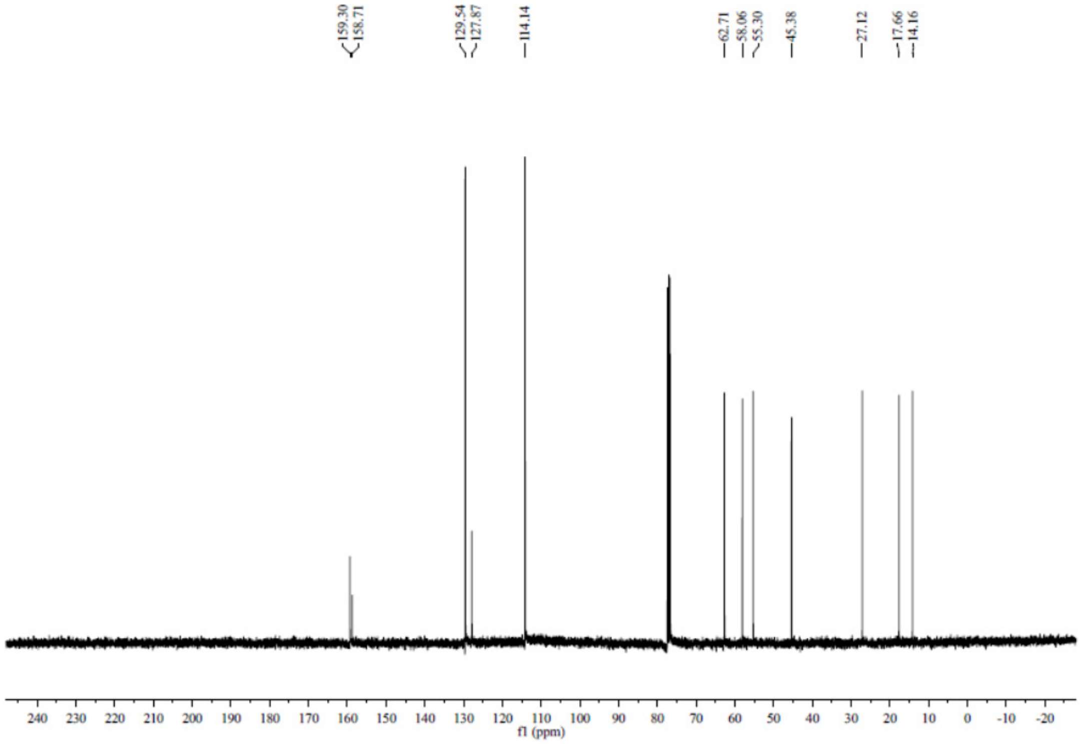
159.30
158.71
129.54
127.87
114.14
62.71
58.06
55.30
45.38
27.12
17.66
14.16
f1 (ppm)

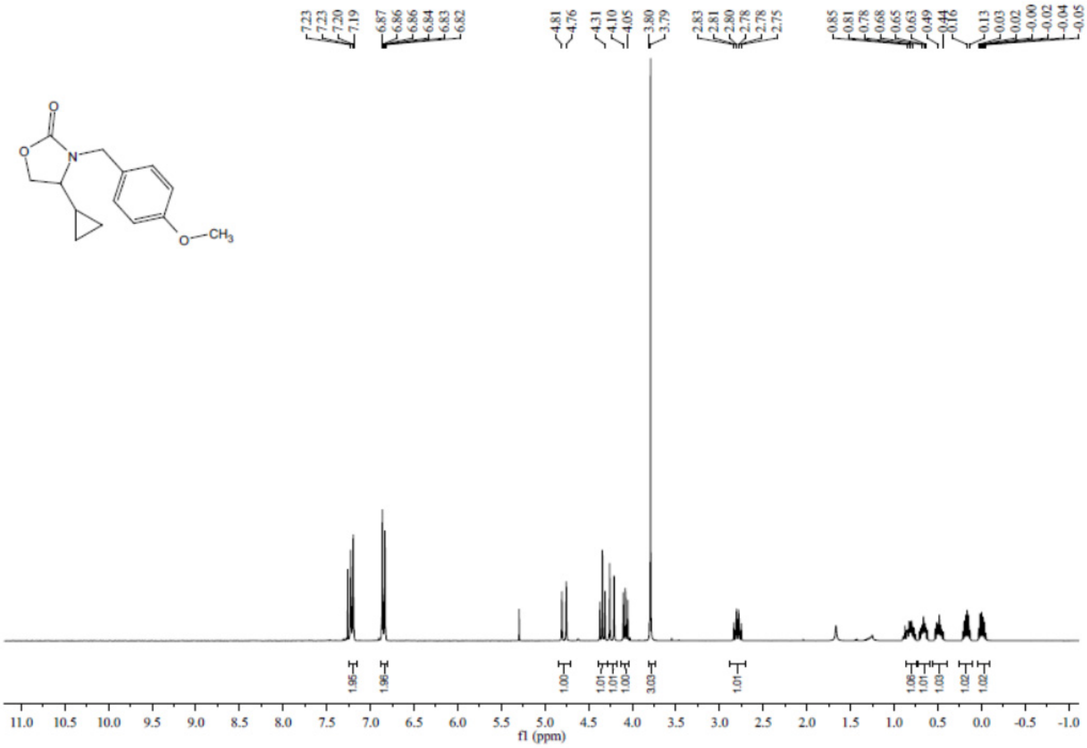
7.23
7.23
7.20
7.19
6.87
6.86
6.86
6.84
6.83
6.82
4.81
4.76
4.31
4.10
4.05
3.80
3.79
2.83
2.81
2.80
2.78
2.78
2.75
0.85
0.81
0.78
0.68
0.65
0.63
0.49
0.16
0.13
0.03
0.02
-0.00
-0.02
-0.04
-0.05
1.95
1.98
1.00
1.01
1.01
1.00
3.03
1.01
1.08
1.01
1.03
1.02
1.02
f1 (ppm)

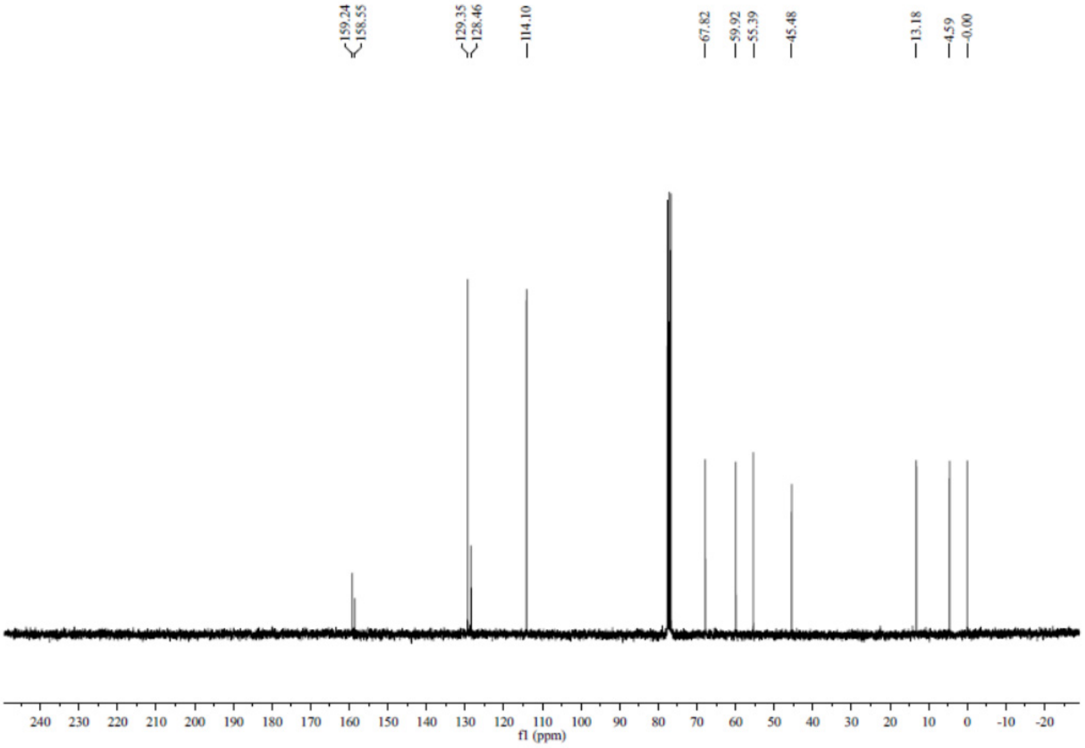
159.24
158.55
129.35
128.46
114.10
67.82
59.92
55.39
45.48
13.18
4.59
0.00
f1 (ppm)

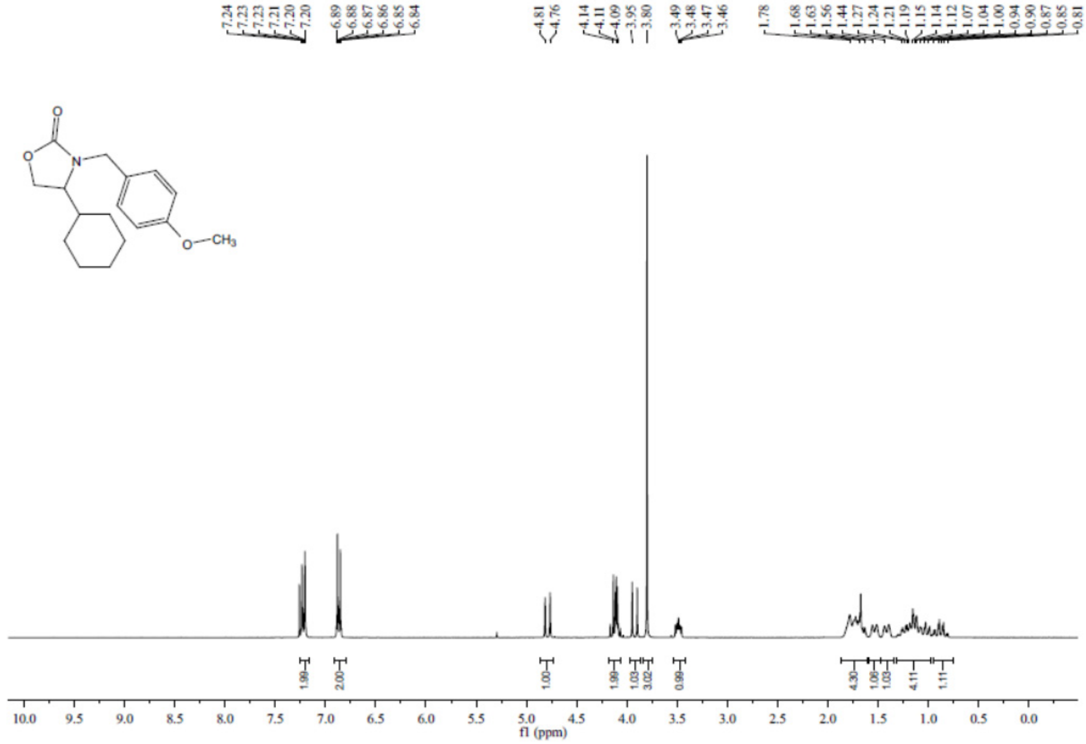
7.24 7.23 7.23 7.21 7.20 7.20
6.89 6.88 6.87 6.86 6.85 6.84
4.81 4.76
4.14 4.11 4.09
3.95
3.80
3.49 3.48 3.47 3.46
1.78 1.68 1.63 1.56 1.44 1.27 1.24 1.21 1.19 1.15 1.14 1.12 1.07 1.04 1.00 0.94 0.90 0.87 0.85 0.81
O
N
O
O
CH3
1.99 2.00 1.00 1.98 1.03 3.02 0.99 4.30 1.08 1.03 4.11 1.11
f1 (ppm)

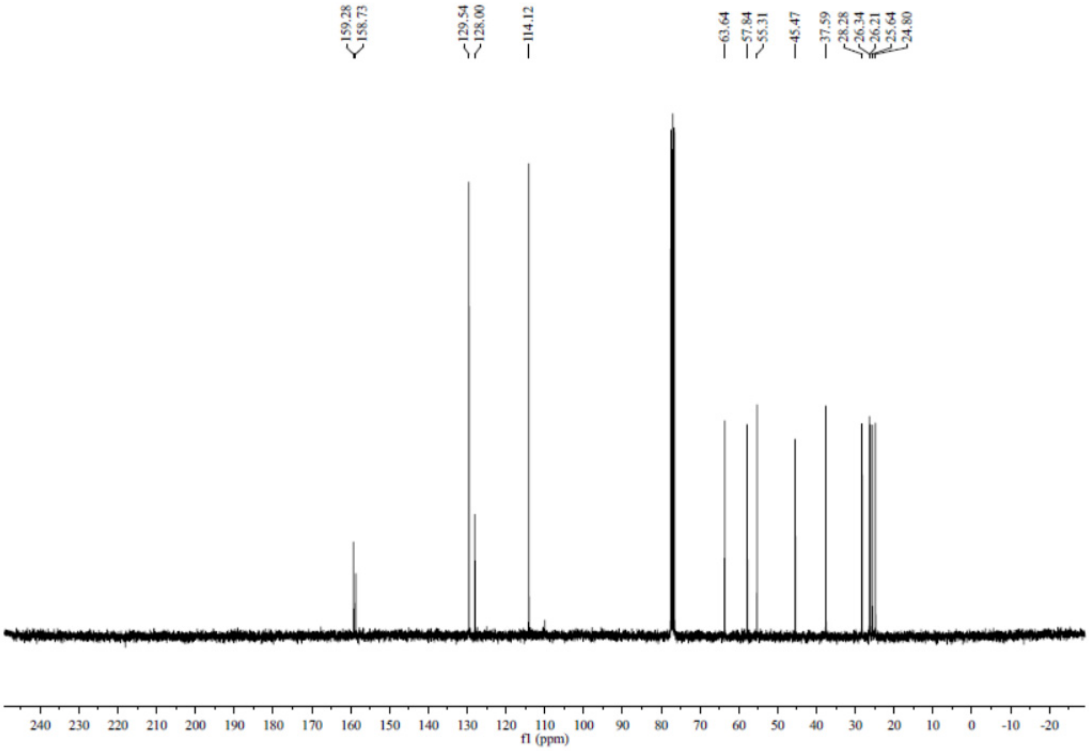
159.28 158.73
129.54 128.00
114.12
63.64 57.84 55.31
45.47
37.59 28.28 26.34 26.21 25.64 24.80
f1 (ppm)

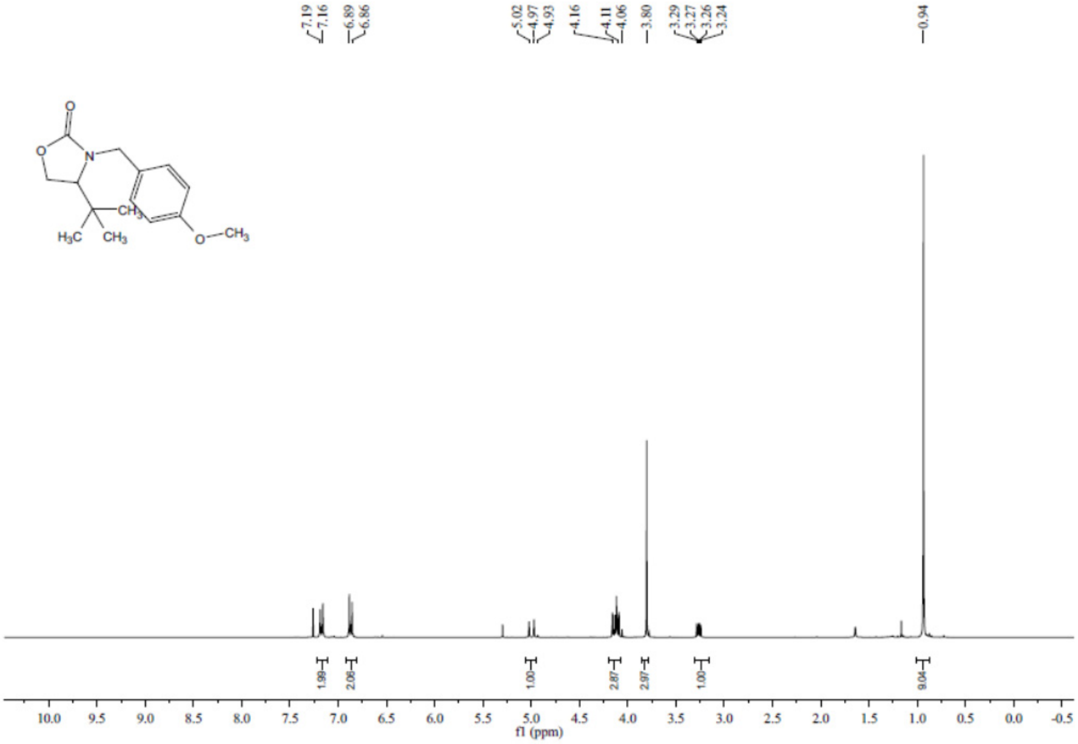
7.19
7.16
6.89
6.86
5.02
4.97
4.93
4.16
4.11
4.06
3.80
3.29
3.27
3.26
3.24
0.94
1.99
2.08
1.00
2.87
2.97
1.00
9.04
f1 (ppm)

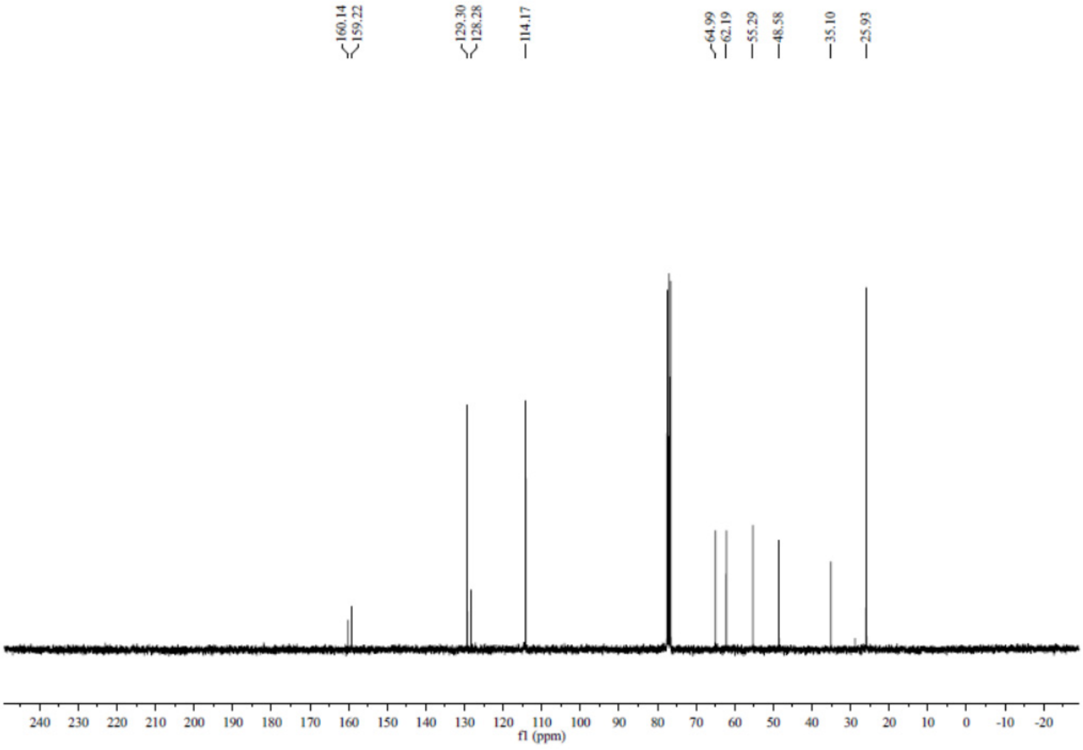
160.14
159.22
129.30
128.28
114.17
64.99
62.19
55.29
48.58
35.10
25.93
f1 (ppm)

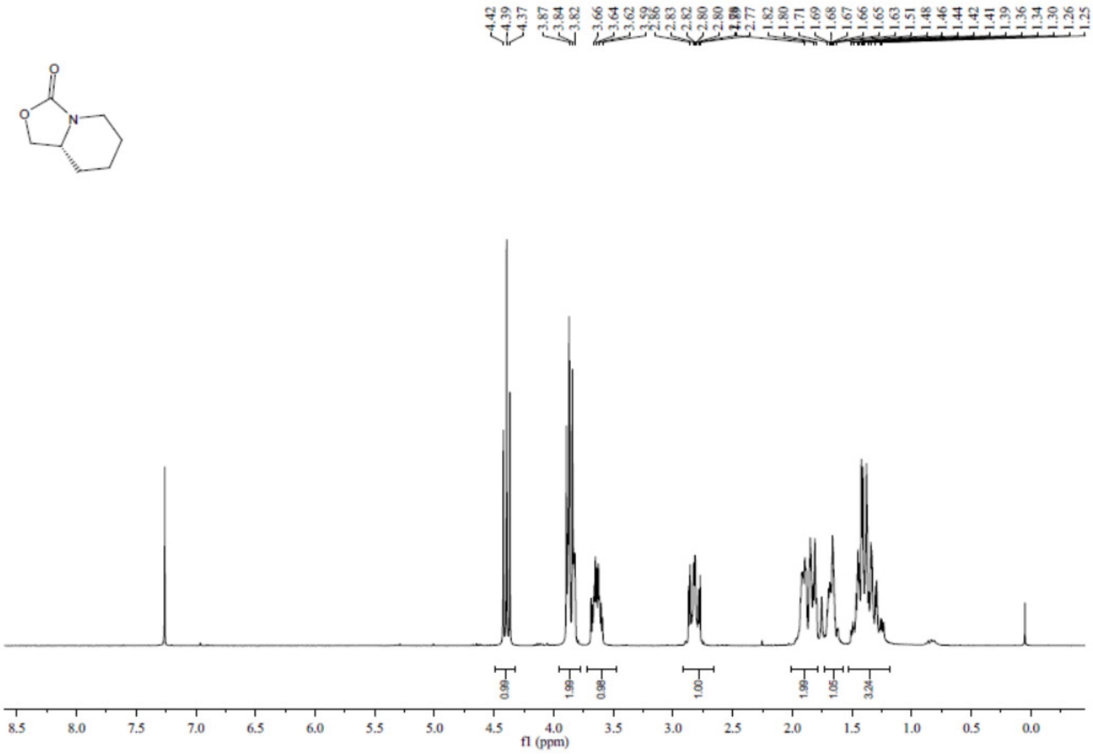
O
O
N
4.42
4.39
4.37
3.87
3.84
3.82
3.66
3.64
3.62
2.83
2.82
2.80
2.80
2.77
1.82
1.80
1.71
1.69
1.68
1.67
1.66
1.65
1.63
1.51
1.48
1.46
1.44
1.42
1.41
1.39
1.36
1.34
1.30
1.26
1.25
0.99
1.99
0.98
1.00
1.99
1.05
3.24
8.5 8.0 7.5 7.0 6.5 6.0 5.5 5.0 4.5 4.0 3.5 3.0 2.5 2.0 1.5 1.0 0.5 0.0
f1 (ppm)

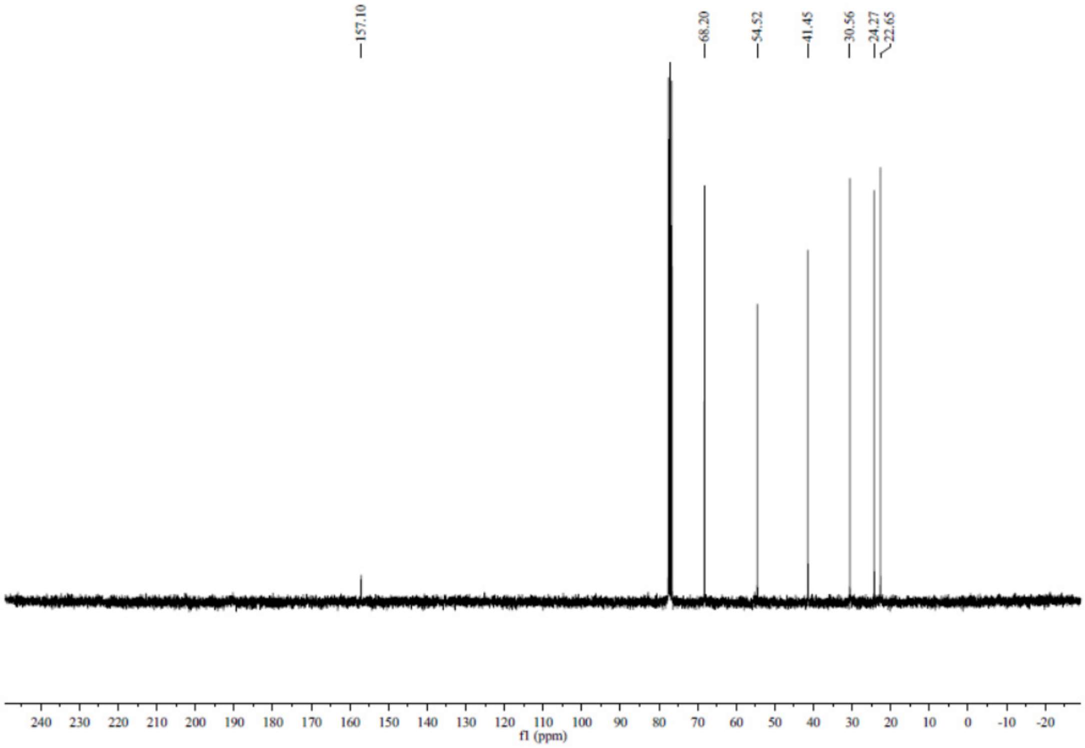
157.10
68.20
54.52
41.45
30.56
24.27
22.65
240 230 220 210 200 190 180 170 160 150 140 130 120 110 100 90 80 70 60 50 40 30 20 10 0 -10 -20
f1 (ppm)

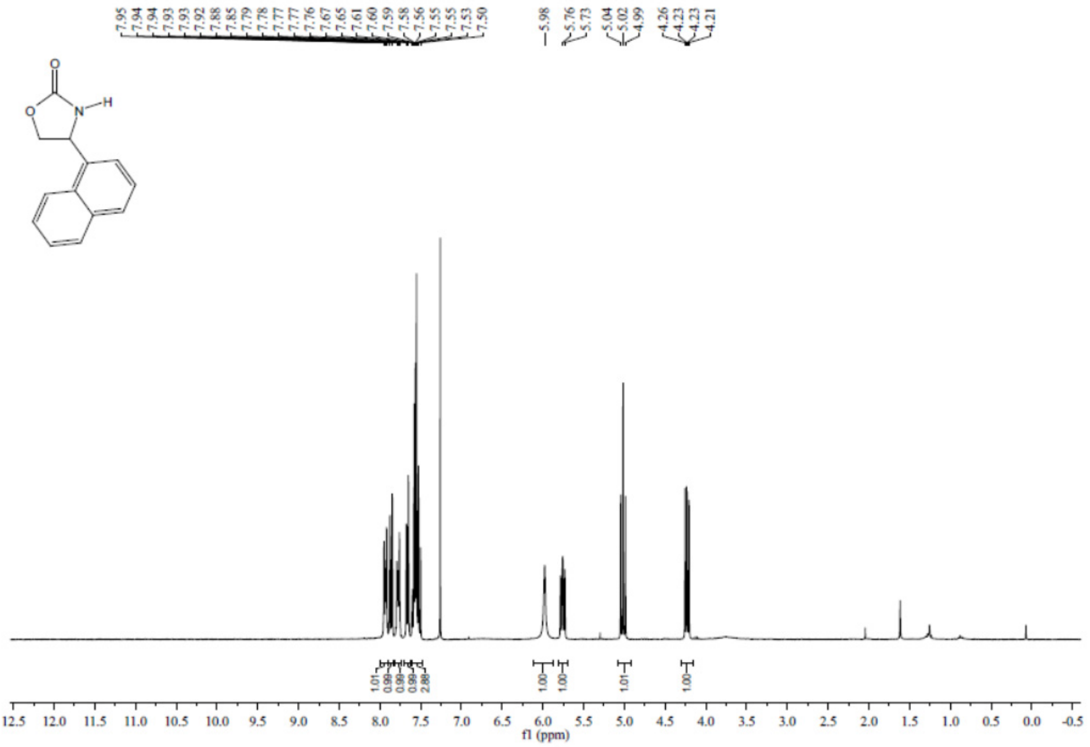

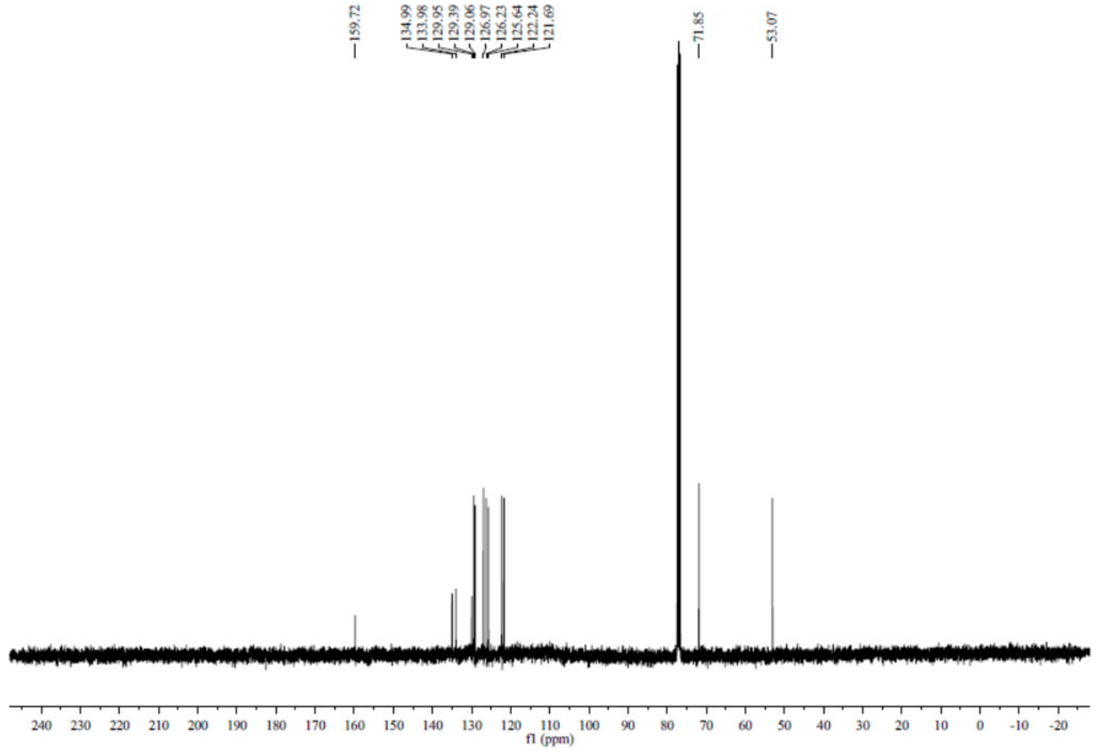

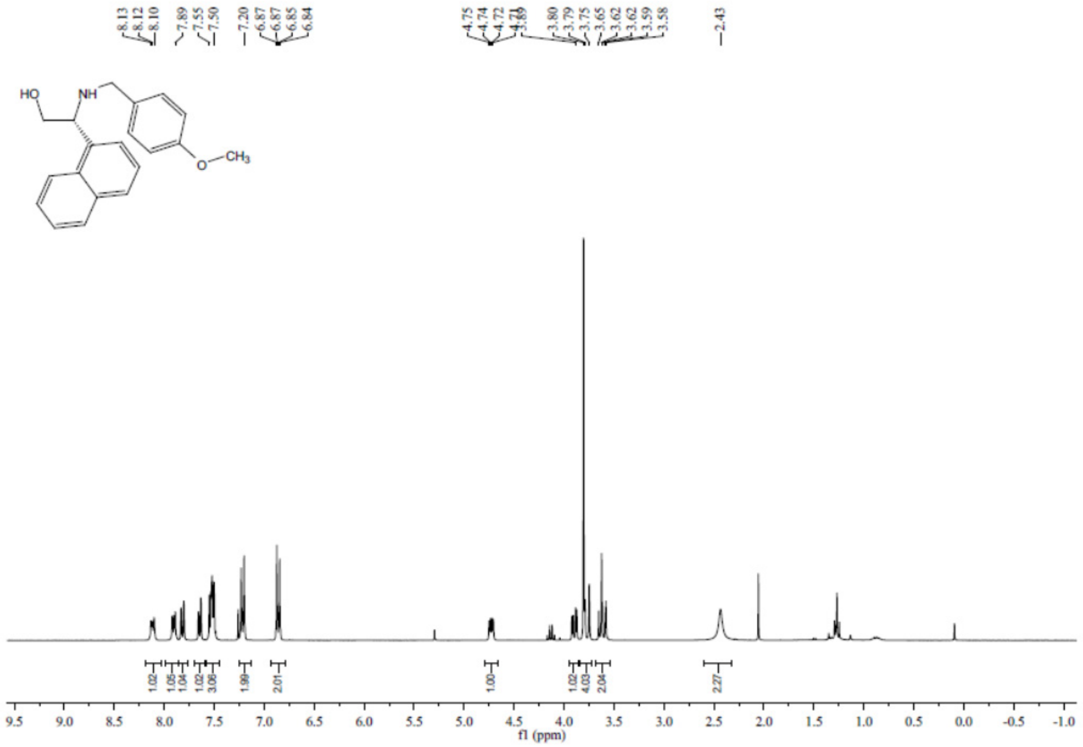
HO
NH
O
CH3
8.13
8.12
8.10
7.89
7.55
7.50
7.20
6.87
6.87
6.85
6.84
4.75
4.74
4.72
4.71
3.89
3.80
3.79
3.75
3.65
3.62
3.62
3.59
3.58
2.43
1.02
1.05
1.04
1.02
3.06
1.99
2.01
1.00
1.02
4.03
2.04
2.27
f1 (ppm)

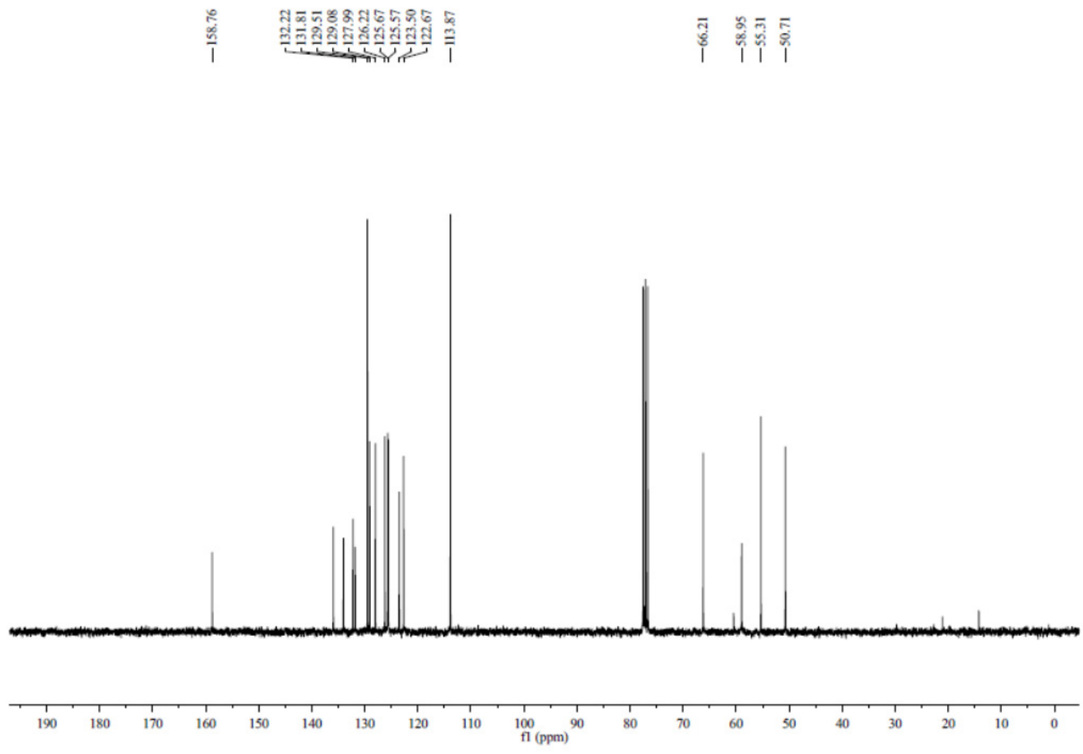
158.76
132.22
131.81
129.51
129.08
127.99
126.22
125.67
125.57
123.50
122.67
113.87
66.21
58.95
55.31
50.71
f1 (ppm)

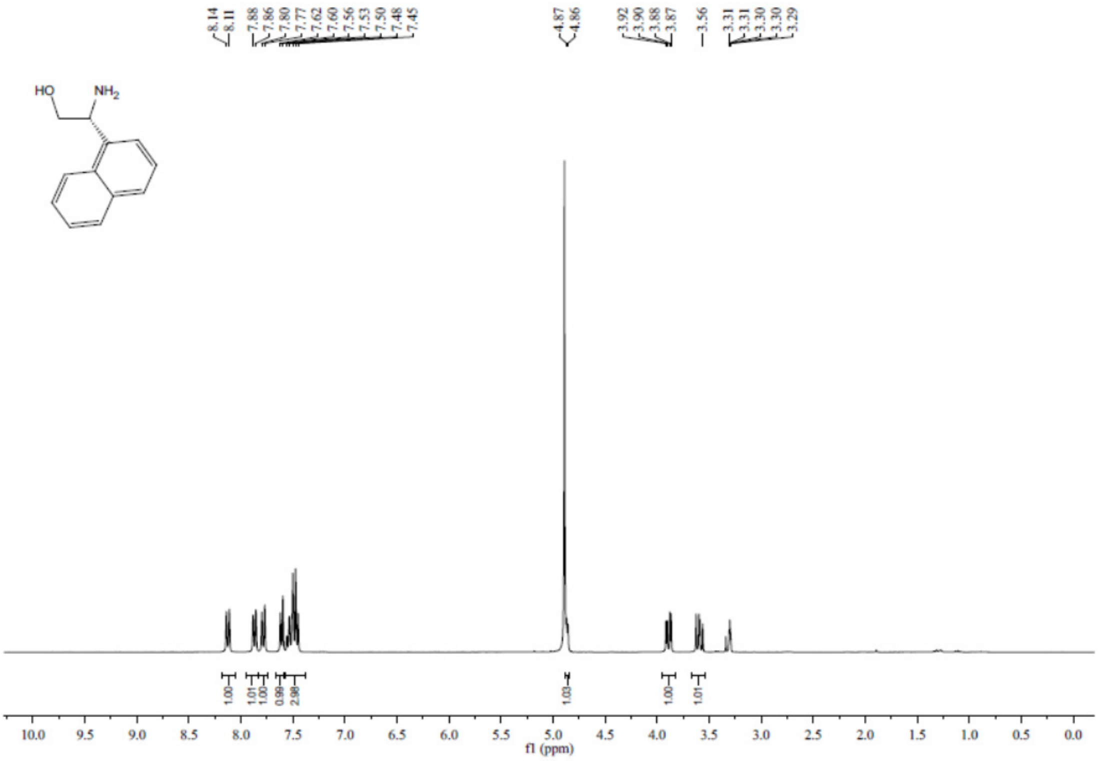
HO
NH2
8.14
8.11
7.88
7.86
7.80
7.77
7.62
7.60
7.56
7.53
7.50
7.48
7.45
4.87
4.86
3.92
3.90
3.88
3.87
3.56
3.31
3.31
3.30
3.30
3.29
1.00
1.01
1.00
0.99
2.98
1.03
1.00
1.01
f1 (ppm)

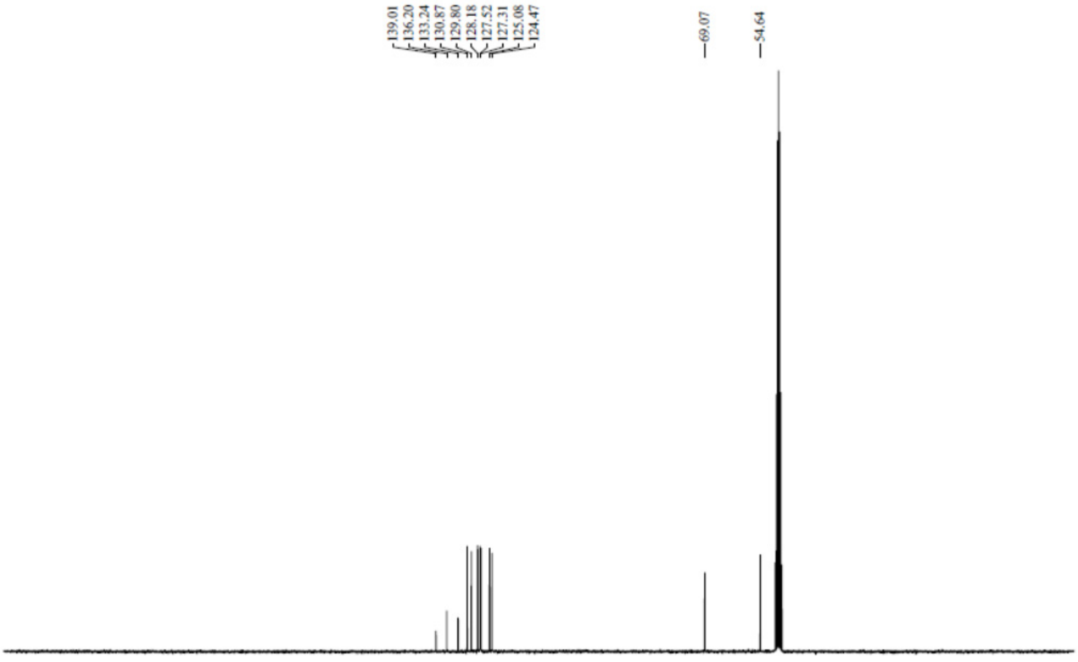
139.01
136.20
133.24
130.87
129.80
128.18
127.52
127.31
125.08
124.47
69.07
54.64
f1 (ppm)

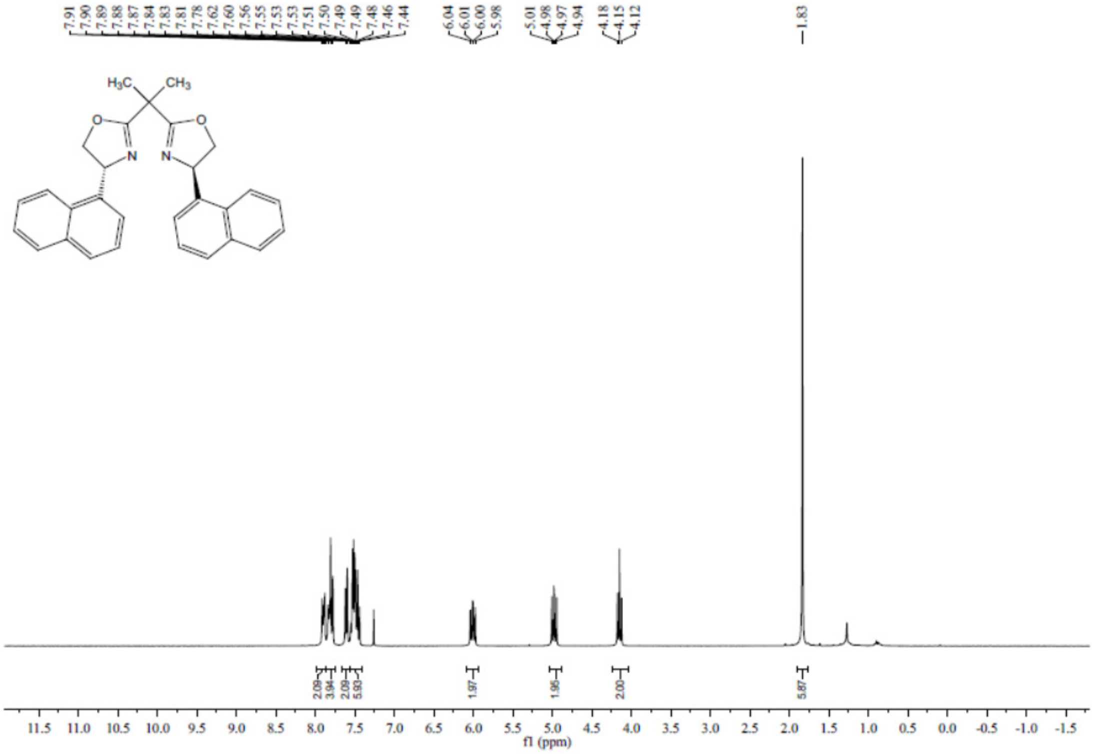
7.91
7.90
7.89
7.88
7.87
7.84
7.83
7.81
7.78
7.62
7.60
7.56
7.55
7.53
7.53
7.51
7.50
7.49
7.49
7.48
7.46
7.44
6.04
6.01
6.00
5.98
5.01
4.98
4.97
4.94
4.18
4.15
4.12
1.83
H3C
CH3
O
N
N
O
2.09
3.94
2.09
5.93
1.97
1.95
2.00
5.87
11.5 11.0 10.5 10.0 9.5 9.0 8.5 8.0 7.5 7.0 6.5 6.0 5.5 5.0 4.5 4.0 3.5 3.0 2.5 2.0 1.5 1.0 0.5 0.0 -0.5 -1.0 -1.5
f1 (ppm)

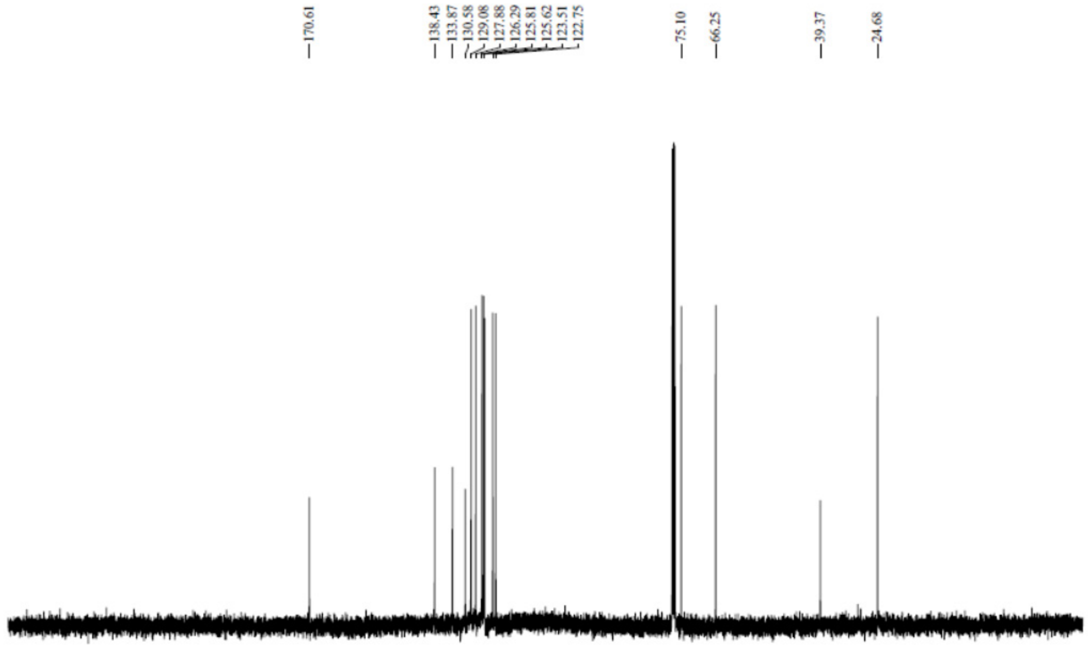
170.61
138.43
133.87
130.58
129.08
127.88
126.29
125.81
125.62
123.51
122.75
75.10
66.25
39.37
24.68
240 230 220 210 200 190 180 170 160 150 140 130 120 110 100 90 80 70 60 50 40 30 20 10 0 -10 -20
f1 (ppm)

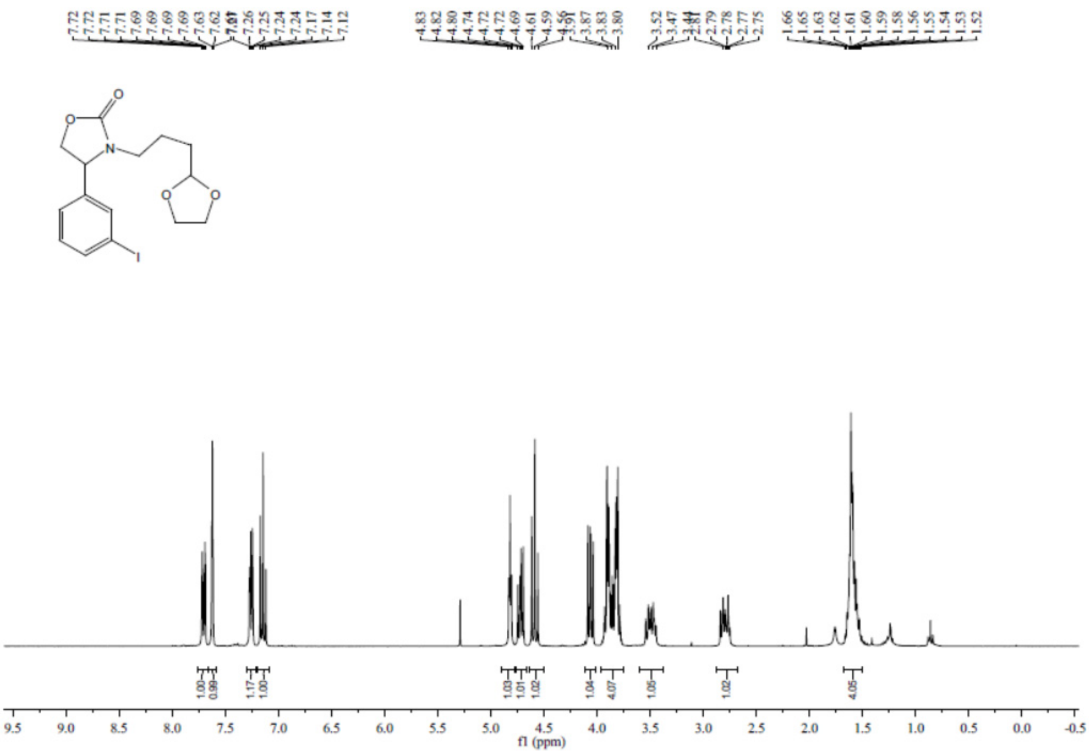

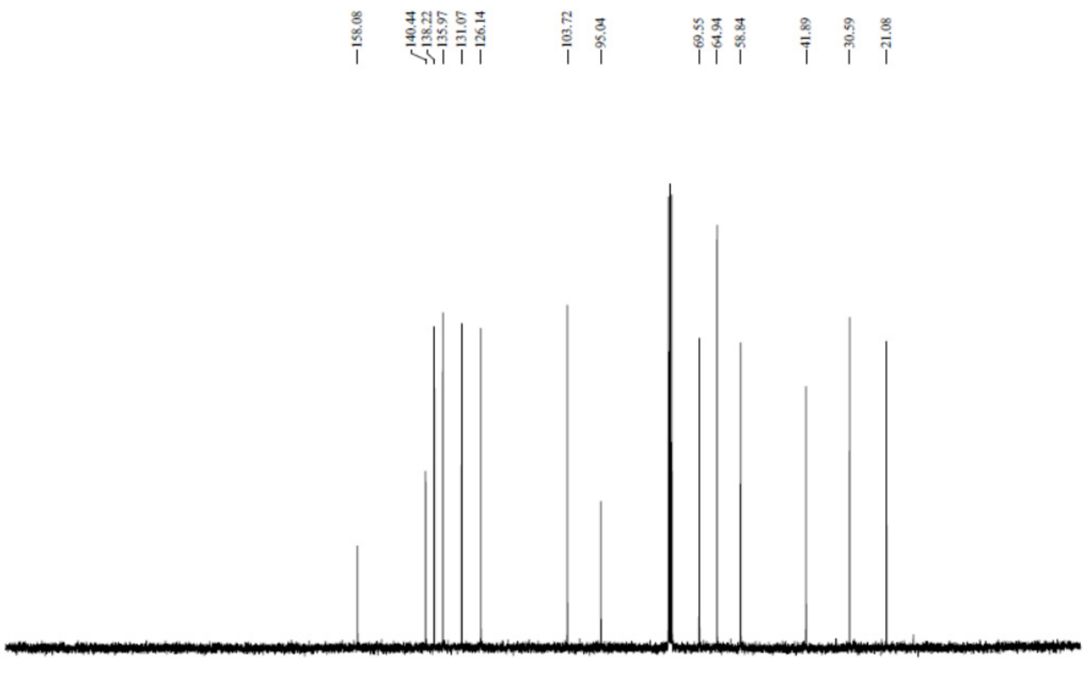

Supplement: Supplementary file 1 [file SC-009-C8SC01869C-s001.pdf]
